# Alteration of Protein Levels during Influenza Virus H1N1 Infection in Host Cells: A Proteomic Survey of Host and Virus Reveals Differential Dynamics

**DOI:** 10.1371/journal.pone.0094257

**Published:** 2014-04-09

**Authors:** Susann Kummer, Max Flöttmann, Björn Schwanhäusser, Christian Sieben, Michael Veit, Matthias Selbach, Edda Klipp, Andreas Herrmann

**Affiliations:** 1 Department of Biology, Faculty of Mathematics and Natural Sciences I, Humboldt University Berlin, Berlin, Germany; 2 Max Delbrück Center for Molecular Medicine, Berlin, Germany; 3 Institute of Virology, Department of Veterinary Medicine, Berlin, Germany; University of Edinburgh, United Kingdom

## Abstract

We studied the dynamics of the proteome of influenza virus A/PR/8/34 (H1N1) infected Madin-Darby canine kidney cells up to 12 hours post infection by mass spectrometry based quantitative proteomics using the approach of stable isotope labeling by amino acids in cell culture (SILAC). We identified 1311 cell proteins and, apart from the proton channel M2, all major virus proteins. Based on their abundance two groups of virus proteins could be distinguished being in line with the function of the proteins in genesis and formation of new virions. Further, the data indicate a correlation between the amount of proteins synthesized and their previously determined copy number inside the viral particle. We employed bioinformatic approaches such as functional clustering, gene ontology, and pathway (KEGG) enrichment tests to uncover co-regulated cellular protein sets, assigned the individual subsets to their biological function, and determined their interrelation within the progression of viral infection. For the first time we are able to describe dynamic changes of the cellular and, of note, the viral proteome in a time dependent manner simultaneously. Through cluster analysis, time dependent patterns of protein abundances revealed highly dynamic up- and/or down-regulation processes. Taken together our study provides strong evidence that virus infection has a major impact on the cell status at the protein level.

## Introduction

The evolution of viruses is accompanied by an opposing evolution through constant interaction with their host. The high risk of infection by viruses that continually adapt strategies to overcome the cellular antiviral-defense is exemplified in the case of influenza A viruses by seasonal as well as pandemic outbreaks with serious consequences for the human population. For decades, enormous efforts are going on to understand the molecular details of viral replication itself, the complex interplay between viruses and hosts, and the corollary for the host cell to combat viral infection. Virus replication is strongly dependent on the biochemical, physiological, and physical status of the infected host cell. This is due to the involvement of various distinct cellular processes, several resources, and competing cellular requirements, such as biosynthesis and transport machinery during the infection process [1].

The envelope of influenza A virus contains two major surface proteins, hemagglutinin (HA) and neuraminidase (NA), and - with a low abundance - the proton channel M2. The matrix protein 1 (M1) forms a layer beneath the viral membrane, enveloping eight different RNA segments. These segments are associated with the nucleoprotein (NP) as well as the three polymerase subunits (PA, PB1, PB2) forming viral ribonucleoprotein complexes (vRNPs). Upon binding to the host cell surface, influenza virus enters the cell via endocytic routes. After acidification of the endosome lumen a conformational change of HA triggers fusion of the envelope with the endosomal membrane releasing the segmented virus genome for transport into the nucleus. The genome is further encoding for two regulatory proteins: the nonstructural protein 1 (NS1) which is expressed in the host cell but is not a component of the virion itself, and the nuclear export protein (NEP, synonymous NS2) which is represented in the virus particle in small quantities [2]. NS1 suppresses transport of host mRNA from the nucleus, thus, favoring the export of viral mRNA, while NEP mediates the nuclear export of viral vRNPs [3]–[5].

The invasive hijacking of the host cell machinery by the virus is associated with directed influence on gene expression of host cell proteins [6]. Recently, the mimicry of cellular signal sequences by viral short linear motifs (SLiMs) was assigned to play a key role for viral hijacking of host transport and biosynthesis. So far, certain virus and host cell components interacting with each other have been identified through yeast-two-hybrid assays, computational approaches, and genome-wide RNA interference screens [7]–[12]. These partners include RNA-binding proteins, transport proteins, transcription factors, and proteins of the intra-cellular signaling pathways (NFκB, apoptosis, MAPK, and WNT). However, a comprehensive view on the complex infection process and its consequences in particular for the proteome is still lacking.

To unravel the molecular basis of virus-host-interaction on a systems level systematic approaches are required to conceive the whole replication process. Such deep investigation enables the identification of the viral Achilles heel as a potential target for effective antiviral strategies including respective drugs [13]. Former *in vitro* studies and *in silico* analyses addressing this issue found various host genes being essential for the influenza A virus infection cycle [8], [14]–[16], However, it remained open how modification of gene expression translates into dynamics of the proteome and regulatory networks. Previously, quantitative proteomic analysis of influenza A virus (H1N1) infected A549 cells [17]–[19] and primary human bronchial airway epithelial cells [19] have been performed by stable isotope labeling by amino acids in cell culture (SILAC). Coombs et al. [17] found that about 360 out of almost 4700 characterized cytosolic proteins were differentially regulated upon infection. Those are involved, for example, in cell immunity and antigen presentation, metabolism, signal transduction, and transcription pathways. This analysis was done 24 hrs post infection (p.i.), where significant titers of progeny viruses of H1N1 infected A549 cells have been observed [20]. Hence, the study provides important information on the cell proteome in the phase of intense virus budding, i.e. host cells are in a late state of infection. However, the dynamics of the cell proteome along the whole infection cycle may give important clues how virus components modulate the cell proteome in the different phases of infection and, thereby, on early cellular hubs as potential targets for inhibition of virus infection.

Here, we present a quantitative study on the influenza A /Puerto Rico/8/1934 (H1N1) H1N1 infection triggered time dependent dynamic changes of the proteome of infected Madin-Darby canine kidney (MDCK) cells monitoring for the first time viral and cellular proteins simultaneously. We assessed its impact on the host cell proteome by mass spectrometry based quantitative proteomics using the SILAC approach [21]–[23].

## Results/Discussion

**Figure 1:**
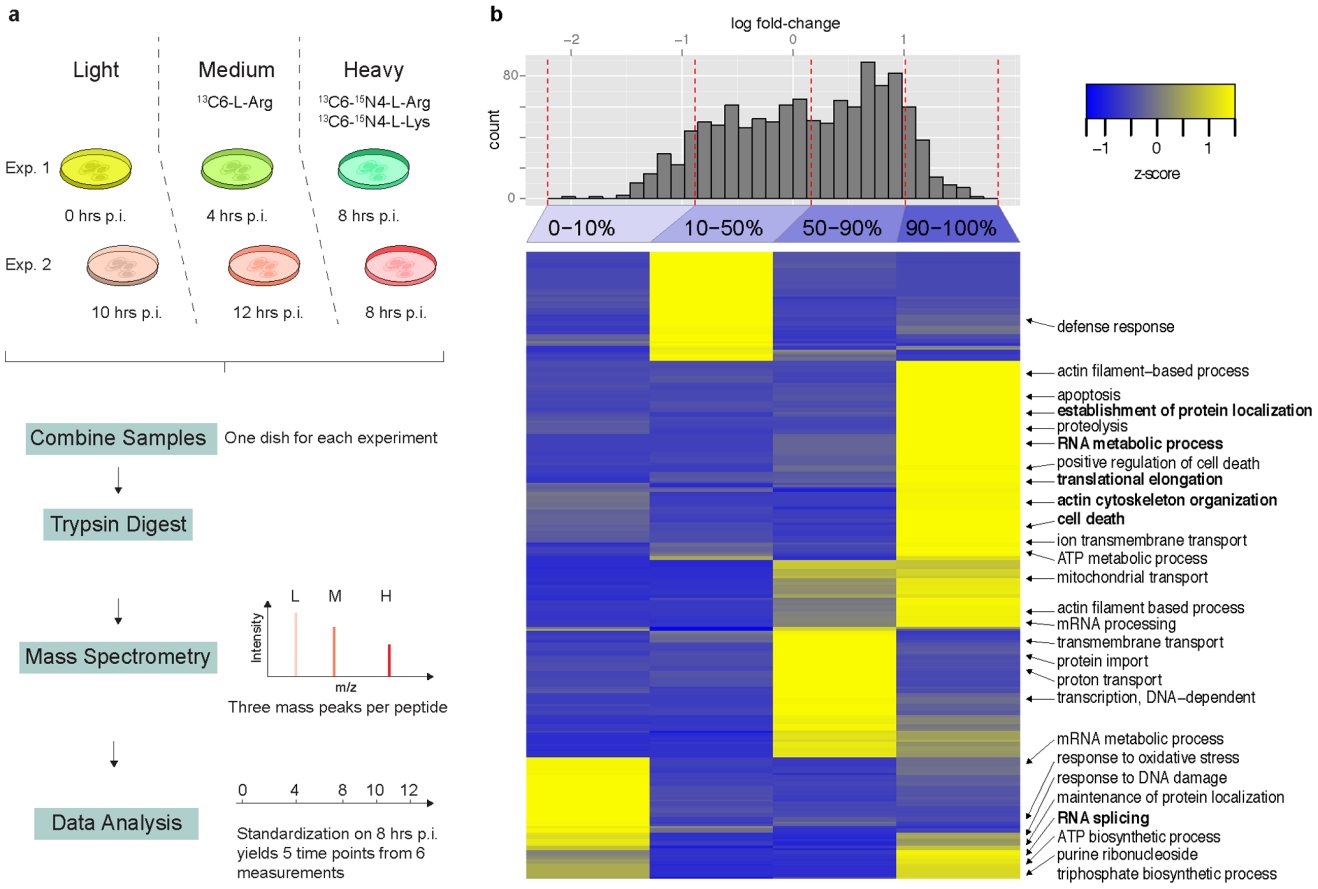
a, Outline of the experimental setup (For details see Material and Methods.). b, Proteomic phenotyping of the influenza A/PR/8 infected MDCK cell proteome using GO annotations. Quantiles of the quantification histogram are indicated at the top of the heatmap. Each quantile was separately analyzed for gene ontology pathways and clustered for the z transformed p values. The most prominent representatives of (Table S2) -represented biological processes of each quantile were selected and annotated in the right panel.

To analyze dynamic processes along the whole viral infection cycle we generated quantitative data from the viral and the host cell proteome of influenza virus infected (MDCK) cells after various time points post infection (p.i.) by mass spectrometry analyses of SILAC samples. MDCK cells are of epithelial origin and serve as a model cell system for human epithelial cells since this cell line was used extensively to study influenza A virus [24]. The experimental procedure is illustrated in Figure 1a. In brief, MDCK cells were maintained in SILAC media containing variants of stable isotope labeled essential amino acids. The incorporation of these amino acids into newly synthesized proteins results in “light”, “medium-heavy” and “heavy” cell populations [25]. Cells were infected with partially purified virus at an empirically determined amount of virus sufficient to infect nearly all cells. HA titration of the stock suggested this was equivalent to an MOI of 100. Infection efficiency was ∼98% as indicated by staining with antinucleoprotein antibodies (see SI and Figure S1). After infection the cells were harvested at various time points up to 12 hrs p.i., digested by trypsin, and analyzed by mass spectrometry. This period covers also the onset of virus budding from infected MDCK cells, which occurs already at about 10 hrs p.i. [26]–[28]. The difference in mass of the “light”, “medium-heavy” and “heavy” variants allowed the identification of the distinct fractions at once and thus, the direct comparison of the obtained data sets. The whole experiment was independently replicated showing a good correlation and demonstrating the reproducibility of our assay (Figure S2). We identified 1311 MDCK cell proteins (Figure 1b) and most of influenza A virus proteins (Table S1). In the following, we discuss the results for the virus proteins and for the MDCK cell proteins, as well as consequences for cellular regulatory and metabolic networks.

### Kinetics of virus protein expression

**Figure 2:**
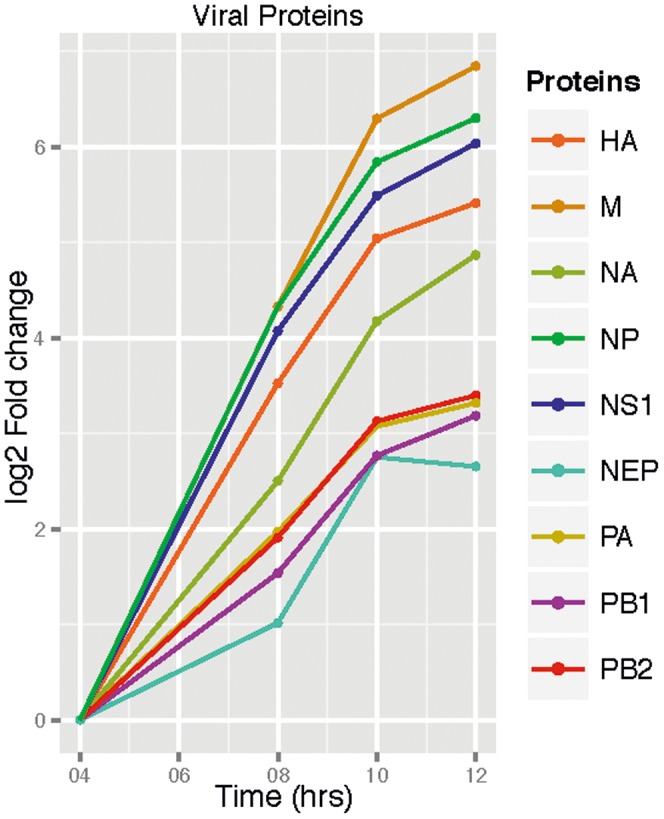
Time profile of viral proteins. Logarithmic presentation of the protein abundance fold change. For details see Material and Methods.

The dynamics of the virus proteome were recorded up to 12 hrs p.i. and are presented in Figure 2. A few of proteins were not detected, for example M2, an alternative splicing variant of segment 7 [29]. As expected, the abundances of the viral proteins showed significant alterations throughout the time course of the experiment. To provide independent evidence for the time course of virus expression measured by SILAC, we have recorded a immunofluorescence time course of NP expression (see Figure S1). NP expression becomes visible between 2 and 4 hrs p.i. with strong accumulation in the nucleus. Nuclear export was rarely observed at 6 hrs p.i., but became clearly visible at later time points. Expression reached a plateau around 10 hrs p.i. as also observed by the SILAC approach.

Protein content fold changes are plotted for ≥4 hrs p.i. setting the amount at 4 hrs p.i. arbitrarily to 0. Note that most virus proteins exhibited an apparent decline in abundance between 0 and 4 hrs p.i. which is due to our experimental set-up: The viral particles used for infection of the three differentially labeled cell populations were light. Combining samples is expected to cause a specific increase in light viral proteins with respect to the others and consequently an overestimation of viral protein abundance at the early phase of infection. Furthermore, this fraction of light virus proteins does not correspond to newly synthesized proteins. As a consequence, we have omitted plotting virus protein content for the early phase of infection. Theoretically, NS1 should not decline since this protein is not part of incoming virus particles. Consistently, we observed that the decline of the protein is considerably lower than for the most other virus proteins (Table S1). The minor decline we detected might be due to measurement errors. A similar low decline was found for NEP which is only at a very low copy number present in virions.

For >4 hrs p.i. a strong synthesis of all viral proteins was observed. Data for ≥10 hrs p.i. indicate that protein synthesis has reached a plateau. However, based on protein abundance two groups can be identified. Protein expression of HA, M1, NP, NS1, and – although somewhat lower – NA is much steeper (group 1) in comparison to NEP, PA, PB1, and PB2 (group 2). As outlined below the difference between viral protein levels is in line with the function and the abundance of proteins in genesis and formation of new virions. For example, HA, M1, NA belonging to group 1 are the most abundant protein components of a virion.

NS1 shows a strong and continuous increase in abundance in the early phase of influenza A protein translation (Figure 2). This may ensure its efficiency in antagonizing the antiviral response of the host cell. As a non-structural protein it has been shown to influence mRNA splicing [30], mRNA nuclear export [3], mRNA adenylation [31] and translation [32], [33]. In particular, suppression of human mRNA nuclear export favors export of viral mRNA which is an important early step for synthesis of viral proteins. Furthermore, previous studies surmise that NS1 impedes cellular antiviral defense mechanisms [6], [34] by binding to a wide range of cellular molecules [3]. In fact, recent findings by Davey et al. 2011 [35] report about a mechanism that allows viruses to interact with cellular proteins to hijack host resources. For NS1 a short linear motif (SLiM) was identified suggesting this viral protein to mimic cellular signal sequences and interact with cellular proteins. NS1 is known to inhibit, to bind, and to inactivate the double stranded RNA dependent protein kinase (PKR) which is an important regulator of the anti-viral innate immune response in mammalian cells [36], [37]. However, only recently it was found that NP of influenza A is also involved in inhibition of this enzyme but through a different mechanism [38].

Proteins of group 2 do not belong to components of the virus envelope. At this stage, we can only hypothesize why they are not present in high copy numbers in the initial phase of virus replication. For example, NEP together with M1 and cellular transport factors escorts newly formed vRNPs through the nuclear pore. It has been reported that NEP may shuttle back to the nucleus to catalyze export of further RNP complexes [39] which may provide an explanation for lower copy number of the protein. It is known that the polymerase complex factors PA, PB1, and PB2 are expressed at rather low levels in comparison to other virus proteins [40]. Initial transcription cycles can be performed by the polymerase molecules coming with the infecting viral particles. After nuclear import, new polymerase subunits form trimers enabling further transcription and replication in particular at later stages of the infection [41]. Furthermore, at later time points, the newly synthesized polymerases will be complexed in vRNPs.

The strong synthesis of NP and M1 could be related to resourcing of these proteins for formation of vRNPs, and, in case of M1, also for formation of the budding site. NP is required in large amounts to form the vRNP complexes in concert with the viral antigenomic vRNA molecules [42], [43]. Moreover, the level of NP is assumed to hold the key position for controlling the switch from transcription to replication mode [44]. This is supported by findings from a recent virus-host interaction screen [11] suggesting that NP might be involved in cellular signaling pathways and interfere with host cell responses, similar to the well described NS1 protein. M1 binding to those forming vRNPs is mandatory for subsequent nuclear export of vRNPs [45]. Only after formation of vRNP-M1 complexes, nuclear export becomes possible. This could rationalize the delayed steep increase of synthesis of NEP which is important for this export step [39], [46].

Similar to M1, HA is also synthesized with fast progression after infection. As HA and M1 are transported to the plasma membrane of the host cell from which new virions bud, their steep increasing synthesis strongly supports the view that both are the key players of formation of the budding site. One reasonable scenario is that HA enriches locally in raft domains providing the docking site for other viral components. Several studies have suggested that the cytoplasmic tail of HA is recognized by M1 [47], [48]. In comparison, NA synthesis is less pronounced. This could explain the lower number of copies in the envelope. It may also suggest that a high abundance of this protein is not essential for the genesis of the budding site. Nevertheless, it seems to be of relevance in apical sorting of viral proteins [49].

In general, our data hint at a correlation between the abundance increase of viral proteins during the infection progression and their previously determined copy number in the viral particle. For example, the relative proteins levels of M1, HA, and NA at 12 hrs p.i. (Figure 2) are in good agreement with the molecular composition of progeny viral particles [50].

### Analysis of host cell protein dynamics upon viral infection

**Figure 3:**
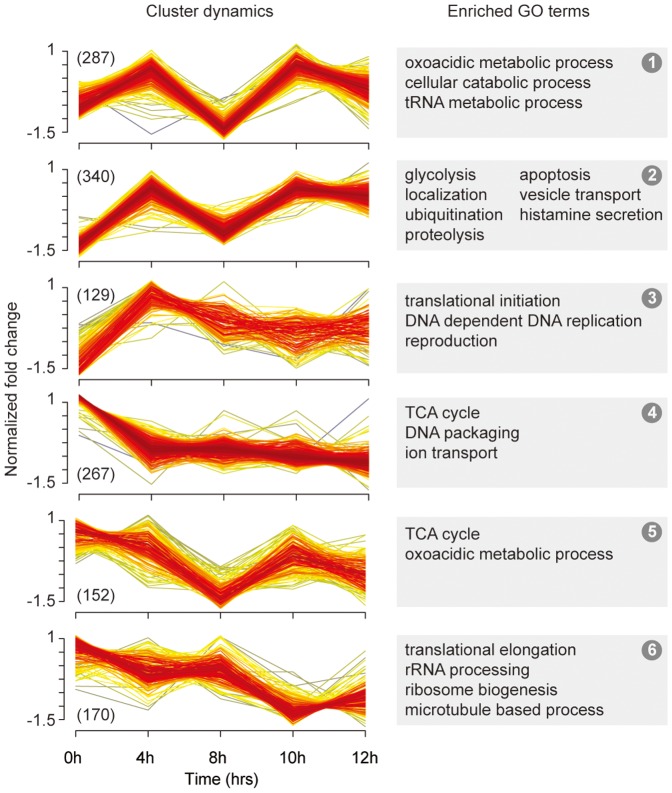
Functional clustering of all estimated cellular proteins. All normalized time profiles were clustered by a fuzzy clustering algorithm to find modules of co-regulated proteins. Enrichment tests for gene ontology terms on each cluster were performed for all proteins with a membership value >0.5 (n =  number in brackets). The most significant terms are represented on the right panel (see also Table S3).

In parallel, we measured and analyzed the host cell response up to 12 hrs p.i. to cover the dynamic changes of the host proteome composition along the time course from virus entry till release of progeny viruses. As a consequence of the integration of newly synthesized viral proteins into the data analysis which are not present at the starting point of the experiment, i.e. at very early stages of the infection, we quantified changes of the MDCK protein abundances relative to 8 hrs p.i., focusing on the identification of large-scale changes (Figure 3). To structure the huge data set (1311 identified proteins), we performed a fuzzy c-means clustering of the time profiles [51] and uncovered co-regulated sets of proteins. Gene ontology (GO) [52] and pathway (KEGG) [53] (Table S3) enrichment tests were used to relate protein sets to biological functions. Generally, proteins could be clustered according to the time dependent patterns of their abundances being either transiently or continuously up- or down-regulated. However, we did not detect an overall breakdown in protein expression as was suggested as consequence of the cap-snatching mechanism necessary for viral transcription initiation [54]. It is more likely that the virus is able to specifically control the cellular gene expression, since some proteins with antiviral function or those with functions less important for viral replication were suppressed and proteins that might support the production of progeny viral particles were increasing in abundance (see below).

Although the expression profile is affected by virus infection, the overall protein expression is not changed much in the time course of our experiments. However, this does not preclude a redistribution of energy provided for different cell processes upon infection as indicated by the differential regulation of protein synthesis [28]. It must also be noted that our experimental set-up quantifies overall changes in protein levels that are affected by both synthesis and degradation. To directly quantify changes in protein production a pulsed SILAC (pSILAC) approach would be more appropriate [25].

### Characterization of host cell protein dynamics upon viral infection

With infection progression a downward trend for abundance of many host cell proteins was observed. Very likely, although ribosomal proteins are in general highly abundant and are expressed constitutively [55], accessibility of ribosomes for translation of cellular proteins becomes restricted due to enhanced translation of virus proteins. We surmise, eventually this will push the cell into an unsustainable state until the cellular biosynthesis collapses at the end of the replication cycle leading to the activation of apoptosis pathways. However, this warrants experimental validation.

GO terms referring essentially to metabolic processes and protein synthesis in clusters 1 to 3 (Figure 3) are enriched in the early phase of infection. Anabolic and catabolic processes including glycolysis (Figure 3, clusters 1 and 2) show a strong increase up to 4 hrs p.i. followed by a temporary decrease approximately at 8 hrs p.i. in the SILAC profile (For additional information please refer to Figure S2.). A similar pattern was revealed for expression of proteins that relate to the “host anti-viral response” with GO terms such as immune response, histamine secretion and apoptosis (Figure 3, cluster 2). We observed a temporary down-regulation of this class of proteins approximately at 8 hrs p.i. This could reflect a virus induced antagonistic “host anti-viral response” activity as it is followed by the onset of production and release of progeny virions. In any case, we observed a strong reactivation of protein synthesis in clusters 1 and 2 along with the onset and increased production of progeny virions (see also above). Remarkably, this increase is accompanied by reduction of ribosome biogenesis, rRNA processing, and translational elongation (cluster 6) indicating enhanced virus protein expression at the expense of expression of cellular proteins. Additionally, among these proteins characterized by a strong and constant increase in abundance were those connected to viral functions and replication cycle: GO terms enriched in cluster 2 (Figure 3) include “localization and transport”, “protein import into nucleus”, “translational initiation”, “actin filament organization” (actin is involved in the formation of budding zones [56]) and “COPI vesicles” (required for post-translational modification of the viral surface proteins and their transport to the plasma membrane [57]). Another significant term was “response to heat” which has been found to be important in vRNA transcription [58]. Surprisingly, proteins that are constantly down regulated are to a large part related to DNA organization (Figure 3, cluster 4).

We observed examples for differential regulation also within metabolism. Expression of proteins responsible for metabolic pathways with activation of glycolysis (Figure 3, cluster 2) behaves differently to that of proteins assigned to cellular respiration (Figure 3, clusters 4 and 5). Notably, the shift from respiration to glycolysis can also be found in proliferating cells or tumor cells [59]–[62]. Whether this dynamic energy compensation mechanism is actively controlled by the virus or the host cell itself remains to be elucidated.

**Figure 4:**
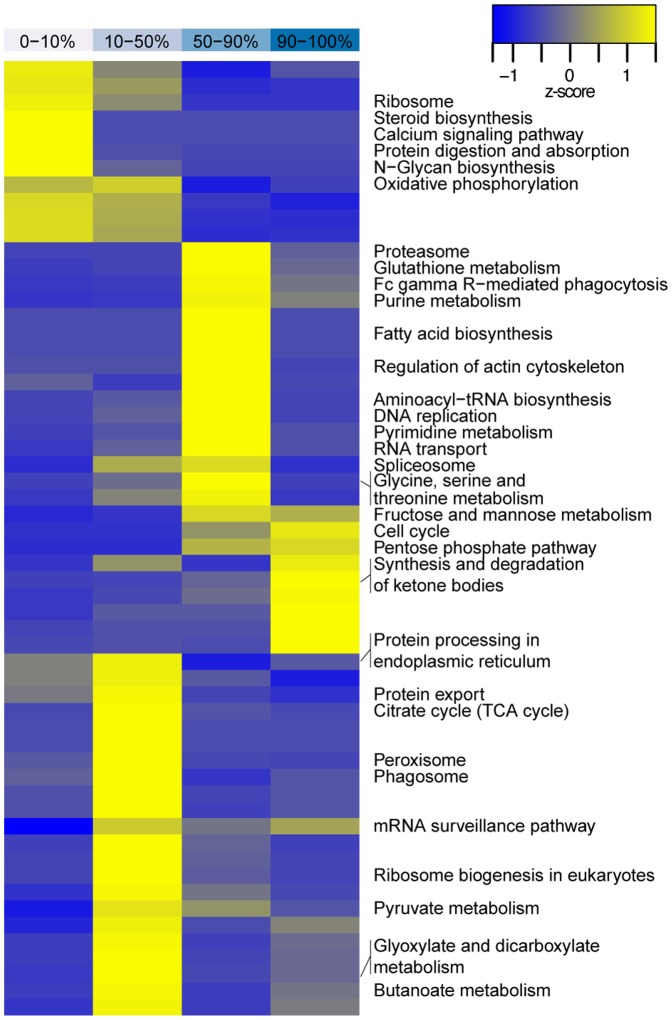
Proteomic phenotyping of the influenza A/PR/8 infected MDCK cell proteome at 10 hrs p.i. using KEGG annotations. Quantiles of the quantification histogram are indicated at the top of the heatmap. Each quantile was separately analyzed for KEGG pathways and clustered for the z transformed p values. The most prominent representatives of all over-represented biological processes of each quantile were selected and annotated in the right panel.

To get deeper insights into the diverse effects of the influenza A virus infection on the host cell proteome we employed proteomic phenotyping [63]. This method characterizes the differences between two proteome samples based on enrichment of classification terms. Profiling was performed using GO (Figure 1b) and KEGG [64] (Figure 4) pathway annotations showing coherent results for the cellular proteome at 10 hrs p.i. where infection is already established but still below the maximum production of new virions.

GO enrichment analysis revealed a strong increase of protein modules relevant (i) for viral replication itself, such as “proton transport”, “RNA metabolism”, “mRNA processing”, “ATP synthesis”, and (ii) for host cell response to infection related processes such as “cell death”. The enhanced expression of proteins assigned to “protein localization” implicated an invasive response of the host cell towards viral infection 10 hrs p.i. This was also reflected by the inverse regulation of the ATP metabolism, which was significantly enriched in the up-regulated genes, but also represented in the down-regulated quantile.

The proteomic phenotyping again revealed the switch from respiration to glycolysis. In the KEGG analysis (Figure 4) the “pentose phosphate shunt” and “ketone body formation” were enriched in the top 10% up-regulated genes, whereas for the “TCA cycle” higher z-scores were reached in the 10–50% range. On closer inspection, abundance of many of the host's glycolytic enzymes and those of the pentose phosphate shunt increased, while enzymes of the TCA cycle dropped slightly in concentration (Figure S3). This gradual shift from aerobic to anaerobic ATP production is in agreement with metabolic data gathered by Ritter et al. 2010 [28]. They reported elevated glycolysis rates and lower respiration after 8 hrs p.i. These changes are probably caused by the onset of apoptotic events in response to infection because the cellular morphology is already changing at this phase of infection (data not shown).

**Figure 5:**
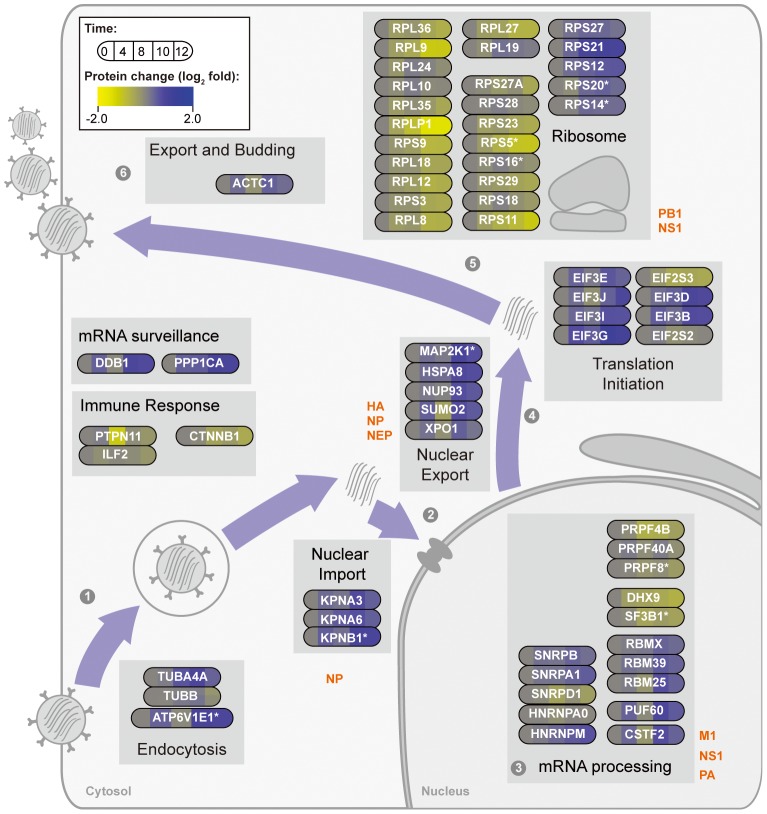
Schematic depiction of the steps of the viral life cycle and the associated host. proteins. Proteins were selected from the overlap between proteins identified by our approach and genes classified as essential for viral reproduction or interacting with viral proteins by Watanabe et al. 2010 [68]. These proteins were grouped according 453 to their function and put into context by the schematic graphics of the infection cycle.

To include previous knowledge on host virus interaction in our analysis, we used gene interaction networks compiled by Watanabe et al. 2010 [65]. They combined data from various studies [7], [8], [11], [66] to create networks of host virus interaction and to identify essential genes for virus replication. By combining these with our time-resolved data we were able to create a scheme on the viral influence on the host cell proteome. In our study we identified 26 out of 128 host proteins found in at least two previous screening studies and 80 proteins identified by interaction studies. Now, we can follow the cellular changes taking place in each step of the replication cycle (Figure 5): (i) We detected an increase of abundance of the proton transporter ATP6V1E1 as well as of three other subunits of this transporter. ATP6 is essential for viral endocytosis [67], [68] and acts in concert with HSPA8 (heat shock protein) mediating the uncoating of the clathrin coated vesicle which is important for endosome maturation and thus, the release of the viral genome from the endosome [69]. Notably, HSPA8 showed an adequate qualitative behavior as ATP6. (ii) In the subsequent step the import of vRNPs into the nucleus is facilitated by the nuclear pore complex. Karyopherin β 1 (importin) which was classified as essential for this active transport [70]–[72] and two additional subunits proved to interact with viral proteins were measured in our study and included in the combined network (Figure S4). They all showed similar activation dynamics during the 12 hrs time range (Figure 5 KPNA3, KPNA6, KPNB1) (iii) For an efficient viral mRNA processing, a fine-tuned interaction between host and virus is crucial. The influenza virus pre-mRNA is processed using the cellular splicing machinery and, in doing so, the detection by anti-viral mechanisms of the host is circumvented. We detected 14 proteins, among them RBMX, RBM39, and RBM25, that are related to mRNA processing and interact with viral proteins [65]. (iv) The translation of viral proteins is strongly dependent on the cellular biosynthesis machinery. We identified 26 of the 42 ribosomal proteins that were reported to interact with vRNP [73]. Contrary to the general trend of reduced protein abundance, seven out of these proteins increased with progression of infection. Among all activated proteins which we have identified, three ribosomal proteins have been reported to exhibit extra-ribosomal functions in p53 activation and inhibition of proliferation [74]. This is in accordance with the observed shift from respiration to glycolysis and indicates an overall change in the cellular status. Further, an activation of translation initiation factors was detected, such as EIF2, EIF3 and EIF4, which are essential for efficient viral replication [75]. The positive influence of virus infection on the expression of EIF4A2 known to enhance the cap recognition at ribosomes supports the previous observation on the enormous efforts towards viral translation [76].

In conclusion, considering the impact of the infection on the cellular protein expression profile and the consequences for various cellular networks one can hypothesize that the virus not simply hijacks existing resources – it modifies the whole cell status for its own purpose. It becomes evident that both, the response of the host cell and the emergence of newly synthesized virus components, resemble a dynamic system which would be difficult to overview and to understand by investigating its numerous parts separately. As we have shown here, our and complementary approaches enable to follow the dynamics of the viral and cellular proteomes quantitatively and the consequences on cellular networks along the entire virus infection cycle. This proteomic approach on a systems level paves the way for new strategies to combat viral infection. For example, extending this holistic approach by comparison of the proteome pattern of infected host cells between different influenza virus strains and correlation with the respective infectivity should highlight main checkpoints of the host cell essential for a permissive infection. Subsequent studies will unravel whether those checkpoints may serve as targets for antiviral drugs.

## Material and Methods

### Cell Culture, virus, and sample preparation

Mammalian MDCK cells were cultivated in DMEM supplemented with 10% FCS in a humid incubator at 37°C and 5% CO_2_. Due to the metabolic incorporation of stable isotopic variants of amino acids, the applied FCS had to be dialysed to eliminate the natural amino acids. SILAC media were prepared as described previously [77] “Heavy” and “medium-heavy” SILAC media were prepared by adding 84 mg/l ^13^C_6_^15^N_4_ L-arginine plus 146 mg/l ^13^C_6_^15^N_2_ L-lysine and by adding 84 mg/l ^13^C_6_-L-arginine plus 146 mg/l D_4_-L-lysine, respectively. Labeled amino acids were obtained from Sigma Isotec (^13^C_6_-L-arginine, ^13^C_6_^15^N_4_ L-arginine and ^13^C_6_^15^N_2_ L-lysine) and Cambridge Isotope Laboratories (D_4_-L-lysine). To prepare “light” SILAC medium, the corresponding non-labeled amino acids (Sigma) were added. MDCK cells were split every 3rd to 4th day in a 1∶10 ratio. Resulting from cultivation in the respective SILAC medium over a period of at least eight passages MDCK cells incorporated uniformly the isotopic amino acids in their proteome.

Cells subjected to virus infection were seeded in 6 well plates at 80% confluency in DMEM overnight. Influenza A virus /Puerto Rico/ 8/1934 (H1N1) was produced in 10-day-old chicken embryos and purified as described previously [78]. The allantoic fluid was collected, and cell debris was removed by a low speed spin (880×*g*, 30 min). The virus was pelleted by spinning the allantoic fluid at 95,000×*g* for 90 min. The pellet was resuspended in PBS and homogenized with a Teflon-coated homogenizer. The HA titer was determined by hemagglutination of red blood cells (RBCs). One hemagglutinating unit corresponds to a titer of 1,6×10 6 plaque forming units.

In all indicated experiments infection with influenza A virus/ Puerto Rico/ 8/1934 (H1N1) was performed at a multiplicity of infection (M.O.I.) of 100. To this end, the virus solution was added directly into 1 ml DMEM and added over the cells. After one hour incubation at 37°C to allow the virus to attach to the cell surface, the supernatant was replaced by 1 ml fresh DMEM and incubation was continued. At various time points post infection (indicated in table 1) cells were harvested by trypsination and pooled according to the scheme in table 2. After cell pellets were stored at −20°C for further procedure.

**Table 1:** Scheme of the influenza A/PR/8 infected MDCK cell sample preparation.

Variant	Harvesting time point (hrs)
MDCK cells in light SILAC medium	0, 10 post infection
MDCK cells in medium heavy SILAC medium	4, 12 post infection
MDCK cells in heavy SILAC medium	8 post infection

**Table 2:** Scheme of samples subjected to mass spectrometry.

Sample ID	Composition
Sample I:	0 p.i. (light) +4 hrs p.i. (medium heavy) +8 hrs p.i. (heavy)
Sample II:	10 hrs p.i. (light) +12 hrs p.i. (medium heavy) +8 hrs p.i. (heavy)

### Cell lysis and preparation for in-solution digestion

Cells were lysed in appropriate amounts of denaturation buffer (6 M urea/2 M thiourea in 10 mM HEPES, pH 8.0) for 20 min on ice. The lysates were cleared by centrifugation for 10 min (14,000 rpm at 4°C) and transferred to fresh tubes. Protein samples were reduced for 30 min at RT in 10 mM dithiothreitol solution followed by alkylation for 20 min by 55 mM iodacetamide in the dark at RT. The endoproteinase LysC (Wako) was added (protein:enzyme ratio of 50∶1) and incubated for 4 hrs at room temperature. After dilution of the sample with four times digestion buffer (50 mM ammonium bi-carbonate (NH_4_HCO_3_) in water, pH 8.0), sequence grade modified trypsin (Promega; protein:enzyme ratio of 50∶1) was used for overnight digestion. Trypsin and Lys-C activity was quenched by adding trifluoroacetic acid to adjust the pH to <2, and peptides were extracted and desalted using StageTips [79].

### HPLC and mass spectrometry

Reversed-phase liquid chromatography (rpHPLC) was done on an Eksigent NanoLC – 1D Plus system using self-made fritless C18 microcolumns [80] (75 μm ID packed with ReproSil-Pur C18-AQ 3-μm resin, Dr. Maisch GmbH) connected on-line to the electrospray ion source (Proxeon) of a LTQ-Orbitrap Velos mass spectrometer (Thermo Fisher). Peptide samples were picked up by the autosampler and loaded onto the column with a flow rate of 250 nl/min. Subsequent sample elution was performed at a flow rate of 200 nl/min with a 10 to 60% acetonitrile gradient over 6 hrs in 0.5% acetic acid for online MS analysis. The LTQ-Orbitrap Velos instrument was operated in the data dependent mode (DDA) with a full scan in the Orbitrap followed by up to 20 consecutive MS/MS scans in the LTQ. Precursor ion scans (m/z 300–1700) were acquired in the Orbitrap part of the instrument (resolution R = 60,000; target value of 1×10^6^), while in parallel the 20 most intense ions were isolated (target value of 3,000; monoisotopic precursor selection enabled) and fragmented in the LTQ part of the instrument by collision induced dissociation (CID; normalized collision energy 35%; wideband activation enabled). Ions with an unassigned charge state and singly charged ions were rejected. Former target ions selected for MS/MS were dynamically excluded for 60 s. Total cycle time for one full scan plus up to 20 MS/MS scans was approximately 2 s.

### Processing of mass spectrometry data

Identification and quantification of proteins were performed with the MaxQuant software package [81]. The software automatically extracts, re-calibrates and quantifies isotope clusters and SILAC triplets in the raw data files (heavy labels: Arg10 and Lys8; medium-heavy labels: Arg6 and Lys4; maximum of three labeled amino acids per peptide; polymer detection enabled; top 6 MS/MS peaks per 100 Da). The generated peak lists were searched against a target-decoy database of forward and reversed proteins [82] by the built-in search engine Andromeda [83]. The search was done using an Influenza A virus /Puerto Rico/8/1934 (H1N1) database (obtained from www.biomart.org in July, 2010) and a domestic doc (*Canis familaris*) [84] database. Full tryptic specificity was required and a maximum of two missed cleavages as well as a mass tolerance of 0.5 Da for fragment ions was allowed. The initial mass accuracy cut-off on the precursor ion was 7 ppm but subsequently narrowed down by filtering based on hits to reversed peptides in the target-decoy database. Oxidation of methionine and acetylation of the protein N-terminus were set as variable modifications, carbamidomethylation of cysteines used as a fixed modification. For filtering of peptide identifications, assembly of proteins and re-quantification the following parameters were used in MaxQuant: A minimum peptide length of 6 amino acids was required and the false discovery rates (FDR) were estimated based on matches to reversed sequences in the concatenated target-decoy database. A maximum false discovery rate of 1% at both the peptide and the protein level was permitted. Peptides were assigned to protein groups (that is, a cluster of a base protein plus additional proteins matching to a subset of the same peptides). Protein groups containing matches to proteins from the reversed database or contaminants were discarded. Protein ratios (M/L, H/L, H/M) were calculated as the median of all peptide ratios for a specific protein. Only proteins with at least three peptide ratios at each measuring time point were considered.

### Data analysis

For the clustering we standardized the fold-changes of the time course to mean zero and standard deviation of one. A soft clustering algorithm called fuzzy-c-means from the Mfuzz [51] package in Bioconductor [85] was used. We chose this method, because it is very robust against noise and provides an easy way for posterior filtering of important genes in the different clusters. The method appoints a membership value between zero and one for each protein to each cluster. The parameter m determines the influence of noise on the establishment of cluster centers. Higher values of m decrease the influence of poorly classified data points on the cluster centers. Applying an iterative approach the number of cluster centers c = 6 and the parameter m = 1.8 were defined.

Identified proteins were assigned to their biological process using gene ontology (GO) [52] enrichment analysis. Enrichment for biological process (BP) was tested using a hypergeometric test from the GoStats [86] package in Bioconductor for the single clusters with the complete set of measured genes as a background distribution. This test automatically corrects for the bias resulting from the tree structure of the ontology. The analysis was as well performed for the KEGG [53] pathway annotations in a similar fashion. To characterize the proteomic changes provoked by the influenza A virus infection a proteomic phenotyping for GO terms at the time point 10 hrs p.i. was performed as previously described [87]. We divided the skewed distribution of measured log2 fold changes into four quantiles (10%, 50%, 90%, and 100%) and did a GO enrichment test for each of the quantiles with all measured genes as background. For all GO terms that appeared in one of the quantiles with a p-value <0.05 we did a transformation by -log10(p-value) and computed a z-score by (x-mean(x))/sd(x). Colors in the heatmaps encode the value of these z-values.

## Supporting Information

Figure S1 **Nucleoprotein expression in MDCK cells after infection with influenza A/PR8.** MDCK cells were infected at MOI 100 and incubated at 37°C for the designated time points. The cells were fixed and immunostained using monoclonal anti-NP antibodies (millipore) followed by secondary antibody staining (anti-mouse, Alexa568, life technologies). The cellular DNA was stained with DAPI. NP expression starts between 2 and 4 hrs p.i. with a strong accumulation in the nucleus. Beginning nuclear export was rarely detected at 6 hrs p.i. (see inset), but is clearly visible at later time points.
(PDF)

Figure S2 **Scatterplots of the two independent replicates for all SILAC ratios over the entire time range of the experiment (0–12 hrs p.i.).** The number of data points (counts) in each hexagon is color coded as indicated. Despite negligible outliners the replicates show a good correlation.
(PDF)

Figure S3 **Scheme of metabolic pathways influenced by the viral infection.** Metabolic pathways that are significantly overrepresented in our data set are depicted. In general, glycolytic enzymes increase in abundance, whereas those of the TCA cycle stay constant or decline after viral infection.
(PDF)

Figure S4 **Network of viral-host interaction partners.** This network is a combination of different interaction networks presented by Watanabe et al. [65] and represents the interactions between viral proteins and host proteins as well as protein interactions with the vRNPs. Colored bars for each cellular protein show the respective expression profile during 12 hrs of infection.
(PDF)

Table S1 **List of viral (green) and cellular (blue) proteins with their changes over time.**
(PDF)

Table S2 **Z-scores for all significant GO terms in the four quartiles.**
(PDF)

Table S3 **Links to enriched GO terms for clusters and lists of genes in these clusters.**
(PDF)

Methods S1 **Supporting methods.**
(DOC)

## References

[1] Snijder B, Sacher R, Ramo P, Damm EM, Liberali P, et al (2009) Population context determines cell-to-cell variability in endocytosis and virus infection. Nature 461: 520–523. 19710653 10.1038/nature08282

[2] Portela A, Digard P (2002) The influenza virus nucleoprotein: a multifunctional RNA-binding protein pivotal to virus replication. J Gen Virol 83: 723–734. 11907320 10.1099/0022-1317-83-4-723

[3] Qiu Y, Krug RM (1994) The influenza virus NS1 protein is a poly(A)-binding protein that inhibits nuclear export of mRNAs containing poly(A). J Virol 68: 2425–2432. 7908060 10.1128/jvi.68.4.2425-2432.1994 PMC236720

[4] Alonso-Caplen FV, Krug RM (1991) Regulation of the extent of splicing of influenza virus NS1 mRNA: role of the rates of splicing and of the nucleocytoplasmic transport of NS1 mRNA. Mol Cell Biol 11: 1092–1098. 1824958 10.1128/mcb.11.2.1092-1098.1991 PMC359785

[5] Shapiro GI, Gurney T Jr, Krug RM (1987) Influenza virus gene expression: control mechanisms at early and late times of infection and nuclear-cytoplasmic transport of virus-specific RNAs. J Virol 61: 764–773. 3806797 10.1128/jvi.61.3.764-773.1987 PMC254018

[6] Marazzi I, Ho JS, Kim J, Manicassamy B, Dewell S, et al (2012) Suppression of the antiviral response by an influenza histone mimic. Nature 483: 428–433. 22419161 10.1038/nature10892 PMC3598589

[7] Konig R, Stertz S, Zhou Y, Inoue A, Hoffmann HH, et al (2010) Human host factors required for influenza virus replication. Nature 463: 813–817. 20027183 10.1038/nature08699 PMC2862546

[8] Karlas A, Machuy N, Shin Y, Pleissner KP, Artarini A, et al (2010) Genome-wide RNAi screen identifies human host factors crucial for influenza virus replication. Nature 463: 818–822. 20081832 10.1038/nature08760

[9] Krishnan MN, Ng A, Sukumaran B, Gilfoy FD, Uchil PD, et al (2008) RNA interference screen for human genes associated with West Nile virus infection. Nature 455: 242–245. 18690214 10.1038/nature07207 PMC3136529

[10] Brass AL, Huang IC, Benita Y, John SP, Krishnan MN, et al (2009) The IFITM proteins mediate cellular resistance to influenza A H1N1 virus, West Nile virus, and dengue virus. Cell 139: 1243–1254. 20064371 10.1016/j.cell.2009.12.017 PMC2824905

[11] Shapira SD, Gat-Viks I, Shum BO, Dricot A, de Grace MM, et al (2009) A physical and regulatory map of host-influenza interactions reveals pathways in H1N1 infection. Cell 139: 1255–1267. 20064372 10.1016/j.cell.2009.12.018 PMC2892837

[12] Meyniel-Schicklin L, de Chassey B, Andre P, Lotteau V (2012) Viruses and interactomes in translation. Mol Cell Proteomics 11 : M111 014738. 10.1074/mcp.M111.014738 PMC3394946 22371486

[13] Law GL, Korth MJ, Benecke AG, Katze MG (2013) Systems virology: host-directed approaches to viral pathogenesis and drug targeting. Nat Rev Microbiol 11: 455–466. 23728212 10.1038/nrmicro3036 PMC4028060

[14] Emmott E, Wise H, Loucaides EM, Matthews DA, Digard P, et al (2010) Quantitative proteomics using SILAC coupled to LC-MS/MS reveals changes in the nucleolar proteome in influenza A virus-infected cells. J Proteome Res 9: 5335–5345. 20701360 10.1021/pr100593g

[15] Bortz E, Garcia-Sastre A (2011) Predicting the pathogenesis of influenza from genomic response: a step toward early diagnosis. Genome Med 3: 67. 22023877 10.1186/gm283 PMC3239229

[16] Lietzen N, Ohman T, Rintahaka J, Julkunen I, Aittokallio T, et al (2011) Quantitative subcellular proteome and secretome profiling of influenza A virus-infected human primary macrophages. PLoS Pathog 7: e1001340. 21589892 10.1371/journal.ppat.1001340 PMC3093355

[17] Coombs KM, Berard A, Xu W, Krokhin O, Meng X, et al (2010) Quantitative proteomic analyses of influenza virus-infected cultured human lung cells. J Virol 84: 10888–10906. 20702633 10.1128/JVI.00431-10 PMC2950599

[18] Kroeker AL, Ezzati P, Coombs KM, Halayko AJ (2013) Influenza A Infection of Primary Human Airway Epithelial Cells Up-Regulates Proteins Related to Purine Metabolism and Ubiquitin-Related Signaling. Journal of Proteome Research 12: 3139–3151. 23750822 10.1021/pr400464p

[19] Dove BK, Surtees R, Bean TJH, Munday D, Wise HM, et al (2012) A quantitative proteomic analysis of lung epithelial (A549) cells infected with 2009 pandemic influenza A virus using stable isotope labelling with amino acids in cell culture. Proteomics 12: 1431–1436. 22585751 10.1002/pmic.201100470

[20] Hui EK, Smee DF, Wong MH, Nayak DP (2006) Mutations in influenza virus M1 CCHH, the putative zinc finger motif, cause attenuation in mice and protect mice against lethal influenza virus infection. J Virol 80: 5697–5707. 16731908 10.1128/JVI.02729-05 PMC1472591

[21] Mann S-EOM (2006) A practical recipe for stable isotope labeling by amino acids in cell culture (SILAC). Nature Protocols 1: 2650. 17406521 10.1038/nprot.2006.427

[22] Ong SE, Blagoev B, Kratchmarova I, Kristensen DB, Steen H, et al (2002) Stable isotope labeling by amino acids in cell culture, SILAC, as a simple and accurate approach to expression proteomics. Mol Cell Proteomics 1: 376–386. 12118079 10.1074/mcp.m200025-mcp200

[23] Amanchy R, Kalume DE, Pandey A (2005) Stable isotope labeling with amino acids in cell culture (SILAC) for studying dynamics of protein abundance and posttranslational modifications. Sci STKE 2005: pl2. 15657263 10.1126/stke.2672005pl2

[24] Rott R, Orlich M, Klenk HD, Wang ML, Skehel JJ, et al (1984) Studies on the adaptation of influenza viruses to MDCK cells. EMBO J 3: 3329–3332. 6526017 10.1002/j.1460-2075.1984.tb02299.x PMC557858

[25] Schwanhausser B, Gossen M, Dittmar G, Selbach M (2009) Global analysis of cellular protein translation by pulsed SILAC. Proteomics 9: 205–209. 19053139 10.1002/pmic.200800275

[26] Barman S, Nayak DP (2007) Lipid raft disruption by cholesterol depletion enhances influenza A virus budding from MDCK cells. J Virol 81: 12169–12178. 17855515 10.1128/JVI.00835-07 PMC2169012

[27] Barman S, Adhikary L, Kawaoka Y, Nayak DP (2003) Influenza A virus hemagglutinin containing basolateral localization signal does not alter the apical budding of a recombinant influenza A virus in polarized MDCK cells. Virology 305: 138–152. 12504548 10.1006/viro.2002.1731

[28] Ritter JB, Wahl AS, Freund S, Genzel Y, Reichl U (2010) Metabolic effects of influenza virus infection in cultured animal cells: Intra- and extracellular metabolite profiling. BMC Syst Biol 4: 61. 20465796 10.1186/1752-0509-4-61 PMC2890500

[29] Roberts PC, Lamb RA, Compans RW (1998) The M1 and M2 proteins of influenza A virus are important determinants in filamentous particle formation. Virology 240: 127–137. 9448697 10.1006/viro.1997.8916

[30] Marion RM, Zurcher T, de la Luna S, Ortin J (1997) Influenza virus NS1 protein interacts with viral transcription-replication complexes in vivo. J Gen Virol 78 (Pt 10): 2447–2451. 10.1099/0022-1317-78-10-2447 9349463

[31] Nemeroff ME, Barabino SM, Li Y, Keller W, Krug RM (1998) Influenza virus NS1 protein interacts with the cellular 30 kDa subunit of CPSF and inhibits 3′end formation of cellular pre-mRNAs. Mol Cell 1: 991–1000. 9651582 10.1016/s1097-2765(00)80099-4

[32] Chen Z, Li Y, Krug RM (1999) Influenza A virus NS1 protein targets poly(A)-binding protein II of the cellular 3′-end processing machinery. EMBO J 18: 2273–2283. 10205180 10.1093/emboj/18.8.2273 PMC1171310

[33] de la Luna S, Fortes P, Beloso A, Ortin J (1995) Influenza virus NS1 protein enhances the rate of translation initiation of viral mRNAs. J Virol 69: 2427–2433. 7884890 10.1128/jvi.69.4.2427-2433.1995 PMC188917

[34] Cheung TK, Poon LL (2007) Biology of influenza a virus. Ann N Y Acad Sci 1102: 1–25. 17470908 10.1196/annals.1408.001

[35] Davey NE, Trave G, Gibson TJ (2011) How viruses hijack cell regulation. Trends Biochem Sci 36: 159–169. 21146412 10.1016/j.tibs.2010.10.002

[36] Bergmann M, Garcia-Sastre A, Carnero E, Pehamberger H, Wolff K, et al (2000) Influenza virus NS1 protein counteracts PKR-mediated inhibition of replication. J Virol 74: 6203–6206. 10846107 10.1128/jvi.74.13.6203-6206.2000 PMC112122

[37] Lu Y, Wambach M, Katze MG, Krug RM (1995) Binding of the influenza virus NS1 protein to double-stranded RNA inhibits the activation of the protein kinase that phosphorylates the elF-2 translation initiation factor. Virology 214: 222–228. 8525619 10.1006/viro.1995.9937

[38] Sharma K, Tripathi S, Ranjan P, Kumar P, Garten R, et al (2011) Influenza A virus nucleoprotein exploits Hsp40 to inhibit PKR activation. PLoS One 6: e20215. 21698289 10.1371/journal.pone.0020215 PMC3115951

[39] O'Neill RE, Talon J, Palese P (1998) The influenza virus NEP (NS2 protein) mediates the nuclear export of viral ribonucleoproteins. EMBO J 17: 288–296. 9427762 10.1093/emboj/17.1.288 PMC1170379

[40] Privalsky ML, Penhoet EE (1978) Influenza virus proteins: identity, synthesis, and modification analyzed by two-dimensional gel electrophoresis. Proc Natl Acad Sci U S A 75: 3625–3629. 278978 10.1073/pnas.75.8.3625 PMC392838

[41] Boivin S, Cusack S, Ruigrok RW, Hart DJ (2010) Influenza A virus polymerase: structural insights into replication and host adaptation mechanisms. J Biol Chem 285: 28411–28417. 20538599 10.1074/jbc.R110.117531 PMC2937865

[42] Albo C, Valencia A, Portela A (1995) Identification of an RNA binding region within the N-terminal third of the influenza A virus nucleoprotein. J Virol 69: 3799–3806. 7745727 10.1128/jvi.69.6.3799-3806.1995 PMC189097

[43] Baudin F, Bach C, Cusack S, Ruigrok RW (1994) Structure of influenza virus RNP. I. Influenza virus nucleoprotein melts secondary structure in panhandle RNA and exposes the bases to the solvent. EMBO J 13: 3158–3165. 8039508 10.1002/j.1460-2075.1994.tb06614.x PMC395207

[44] Shapiro GI, Krug RM (1988) Influenza virus RNA replication in vitro: synthesis of viral template RNAs and virion RNAs in the absence of an added primer. J Virol 62: 2285–2290. 2453679 10.1128/jvi.62.7.2285-2290.1988 PMC253375

[45] Huang X, Liu T, Muller J, Levandowski RA, Ye Z (2001) Effect of influenza virus matrix protein and viral RNA on ribonucleoprotein formation and nuclear export. Virology 287: 405–416. 11531417 10.1006/viro.2001.1067

[46] Neumann G, Hughes MT, Kawaoka Y (2000) Influenza A virus NS2 protein mediates vRNP nuclear export through NES-independent interaction with hCRM1. EMBO J 19: 6751–6758. 11118210 10.1093/emboj/19.24.6751 PMC305902

[47] Jin H, Leser GP, Zhang J, Lamb RA (1997) Influenza virus hemagglutinin and neuraminidase cytoplasmic tails control particle shape. EMBO J 16: 1236–1247. 9135140 10.1093/emboj/16.6.1236 PMC1169722

[48] Chen BJ, Leser GP, Morita E, Lamb RA (2007) Influenza virus hemagglutinin and neuraminidase, but not the matrix protein, are required for assembly and budding of plasmid-derived virus-like particles. J Virol 81: 7111–7123. 17475660 10.1128/JVI.00361-07 PMC1933269

[49] Nayak DP, Hui EK, Barman S (2004) Assembly and budding of influenza virus. Virus Res 106: 147–165. 15567494 10.1016/j.virusres.2004.08.012 PMC7172797

[50] Lamb RAK, Krug RM (1996) Orthomyxoviridae: The viruses and their replication. In Fields Virology 3: 1353–1445.

[51] Futschik ME, Carlisle B (2005) Noise-robust soft clustering of gene expression time-course data. Journal of bioinformatics and computational biology 3: 965–988. 16078370 10.1142/s0219720005001375

[52] Ashburner M, Ball CA, Blake JA, Botstein D, Butler H, et al (2000) Gene ontology: tool for the unification of biology. The Gene Ontology Consortium. Nature genetics 25: 25–29. 10802651 10.1038/75556 PMC3037419

[53] Kanehisa M (2000) KEGG: Kyoto Encyclopedia of Genes and Genomes. Nucleic Acids Research 28: 27–30. 10592173 10.1093/nar/28.1.27 PMC102409

[54] Shih SR, Krug RM (1996) Surprising function of the three influenza viral polymerase proteins: selective protection of viral mRNAs against the cap-snatching reaction catalyzed by the same polymerase proteins. Virology 226: 430–435. 8955065 10.1006/viro.1996.0673

[55] Schwanhausser B, Busse D, Li N, Dittmar G, Schuchhardt J, et al (2011) Global quantification of mammalian gene expression control. Nature 473: 337–342. 21593866 10.1038/nature10098

[56] Simpson-Holley M, Ellis D, Fisher D, Elton D, McCauley J, et al (2002) A functional link between the actin cytoskeleton and lipid rafts during budding of filamentous influenza virions. Virology 301: 212–225. 12359424 10.1006/viro.2002.1595

[57] Ruigrok RW, Barge A, Durrer P, Brunner J, Ma K, et al (2000) Membrane interaction of influenza virus M1 protein. Virology 267: 289–298. 10662624 10.1006/viro.1999.0134

[58] Momose F, Handa H, Nagata K (1996) Identification of host factors that regulate the influenza virus RNA polymerase activity. Biochimie 78: 1103–1108. 9150891 10.1016/s0300-9084(97)86736-3

[59] Fiume L, Manerba M, Vettraino M, Di Stefano G (2011) Effect of sorafenib on the energy metabolism of hepatocellular carcinoma cells. Eur J Pharmacol 670: 39–43. 21924262 10.1016/j.ejphar.2011.08.038

[60] Salminen A, Kaarniranta K (2010) Glycolysis links p53 function with NF-kappaB signaling: impact on cancer and aging process. J Cell Physiol 224: 1–6. 20301205 10.1002/jcp.22119

[61] Nass N, Kukat A, Seibel P, Bromme HJ, Schinzel R, et al (2009) Advanced glycation end product accumulation in rho(0) cells without a functional respiratory chain. Biol Chem 390: 915–919. 19453272 10.1515/BC.2009.083

[62] Ueno M, Seferynska I, Beckman B, Brookins J, Nakashima J, et al (1989) Enhanced erythropoietin secretion in hepatoblastoma cells in response to hypoxia. Am J Physiol 257: C743–749. 2552819 10.1152/ajpcell.1989.257.4.C743

[63] Pan C, Kumar C, Bohl S, Klingmueller U, Mann M (2009) Comparative proteomic phenotyping of cell lines and primary cells to assess preservation of cell type-specific functions. Mol Cell Proteomics 8: 443–450. 18952599 10.1074/mcp.M800258-MCP200 PMC2649808

[64] Tarca AL, Draghici S, Khatri P, Hassan SS, Mittal P, et al (2009) A novel signaling pathway impact analysis. Bioinformatics 25: 75–82. 18990722 10.1093/bioinformatics/btn577 PMC2732297

[65] Watanabe T, Watanabe S, Kawaoka Y (2010) Cellular networks involved in the influenza virus life cycle. Cell Host Microbe 7: 427–439. 20542247 10.1016/j.chom.2010.05.008 PMC3167038

[66] Dent JE, Kao RR, Kiss IZ, Hyder K, Arnold M (2008) Contact structures in the poultry industry in Great Britain: exploring transmission routes for a potential avian influenza virus epidemic. BMC Vet Res 4: 27. 18651959 10.1186/1746-6148-4-27 PMC2526082

[67] König R, Stertz S, Zhou Y, Inoue A, Hoffmann HH, et al (2009) Human host factors required for influenza virus replication. Nature 463: 813–817. 10.1038/nature08699 PMC2862546 20027183

[68] Karlas A, Machuy N, Shin Y, Pleissner K-P, Artarini A, et al (2010) Genome-wide RNAi screen identifies human host factors crucial for influenza virus replication. Nature 463: 818–822. 20081832 10.1038/nature08760

[69] DeLuca-Flaherty C, McKay DB, Parham P, Hill BL (1990) Uncoating protein (hsc70) binds a conformationally labile domain of clathrin light chain LCa to stimulate ATP hydrolysis. Cell 62: 875–887. 1975516 10.1016/0092-8674(90)90263-e

[70] Mosammaparast N, Pemberton LF (2004) Karyopherins: from nuclear-transport mediators to nuclear-function regulators. Trends Cell Biol 14: 547–556. 15450977 10.1016/j.tcb.2004.09.004

[71] Merle E, Rose RC, LeRoux L, Moroianu J (1999) Nuclear import of HPV11 L1 capsid protein is mediated by karyopherin alpha2beta1 heterodimers. J Cell Biochem 74: 628–637. 10440932

[72] Chook YM, Blobel G (2001) Karyopherins and nuclear import. Curr Opin Struct Biol 11: 703–715. 11751052 10.1016/s0959-440x(01)00264-0

[73] Watanabe T, Watanabe S, Kawaoka Y (2010) Cellular networks involved in the influenza virus life cycle. Cell host & microbe 7: 427–439. 20542247 10.1016/j.chom.2010.05.008 PMC3167038

[74] Warner JR, McIntosh KB (2009) How common are extraribosomal functions of ribosomal proteins? Molecular cell 34: 3–11. 19362532 10.1016/j.molcel.2009.03.006 PMC2679180

[75] Jivotovskaya AV, Valasek L, Hinnebusch AG, Nielsen KH (2006) Eukaryotic translation initiation factor 3 (eIF3) and eIF2 can promote mRNA binding to 40S subunits independently of eIF4G in yeast. Mol Cell Biol 26: 1355–1372. 16449648 10.1128/MCB.26.4.1355-1372.2006 PMC1367198

[76] Hellen CU, Sarnow P (2001) Internal ribosome entry sites in eukaryotic mRNA molecules. Genes Dev 15: 1593–1612. 11445534 10.1101/gad.891101

[77] Ong SE, Mann M (2006) A practical recipe for stable isotope labeling by amino acids in cell culture (SILAC). Nat Protoc 1: 2650–2660. 17406521 10.1038/nprot.2006.427

[78] Korte T, Ludwig K, Krumbiegel M, Zirwer D, Damaschun G, et al (1997) Transient changes of the conformation of hemagglutinin of influenza virus at low pH detected by time-resolved circular dichroism spectroscopy. J Biol Chem 272: 9764–9770. 9092509 10.1074/jbc.272.15.9764

[79] Rappsilber J, Mann M, Ishihama Y (2007) Protocol for micro-purification, enrichment, pre-fractionation and storage of peptides for proteomics using StageTips. Nat Protoc 2: 1896–1906. 17703201 10.1038/nprot.2007.261

[80] Ishihama Y, Rappsilber J, Andersen JS, Mann M (2002) Microcolumns with self-assembled particle frits for proteomics. J Chromatogr A 979: 233–239. 12498253 10.1016/s0021-9673(02)01402-4

[81] Cox J, Mann M (2008) MaxQuant enables high peptide identification rates, individualized p.p.b.-range mass accuracies and proteome-wide protein quantification. Nat Biotechnol 26: 1367–1372. 19029910 10.1038/nbt.1511

[82] Elias JE, Gygi SP (2007) Target-decoy search strategy for increased confidence in large-scale protein identifications by mass spectrometry. Nat Methods 4: 207–214. 17327847 10.1038/nmeth1019

[83] Cox J, Neuhauser N, Michalski A, Scheltema RA, Olsen JV, et al (2011) Andromeda: a peptide search engine integrated into the MaxQuant environment. J Proteome Res 10: 1794–1805. 21254760 10.1021/pr101065j

[84] Lindblad-Toh K, Wade CM, Mikkelsen TS, Karlsson EK, Jaffe DB, et al (2005) Genome sequence, comparative analysis and haplotype structure of the domestic dog. Nature 438: 803–819. 16341006 10.1038/nature04338

[85] Gentleman RC, Carey VJ, Bates DM, Bolstad B, Dettling M, et al (2004) Bioconductor: open software development for computational biology and bioinformatics. Genome biology 5: R80. 15461798 10.1186/gb-2004-5-10-r80 PMC545600

[86] Falcon S, Gentleman R (2007) Using GOstats to test gene lists for GO term association. Bioinformatics (Oxford, England) 23: 257–258. 10.1093/bioinformatics/btl567 17098774

[87] Pan C, Kumar C, Bohl S, Klingmueller U, Mann M (2009) Comparative proteomic phenotyping of cell lines and primary cells to assess preservation of cell type-specific functions. Molecular & cellular proteomics: MCP 8: 443–450. 18952599 10.1074/mcp.M800258-MCP200 PMC2649808

