# Supplementary figures and images for: Alteration of Protein Levels during Influenza Virus H1N1 Infection in Host Cells: A Proteomic Survey of Host and Virus Reveals Differential Dynamics
Source: PLoS One. 2014 Apr 9;9(4):e94257. doi: 10.1371/journal.pone.0094257 (PMC3981805; doi:10.1371/journal.pone.0094257)

2h

4h

6h

8h

10h

12h

DAPI

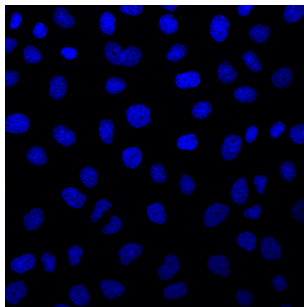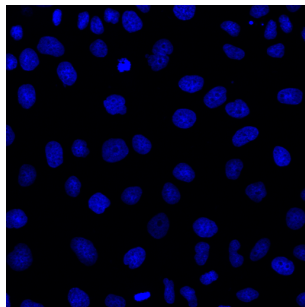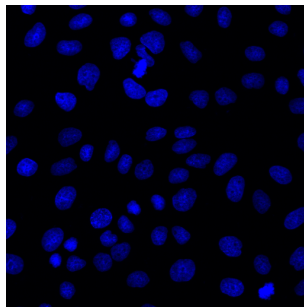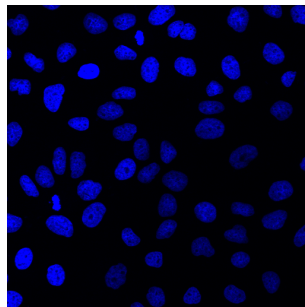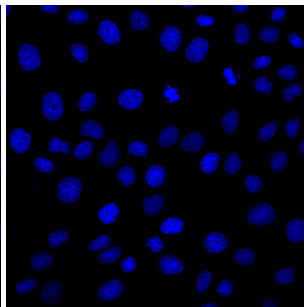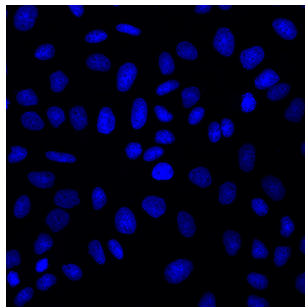

NP

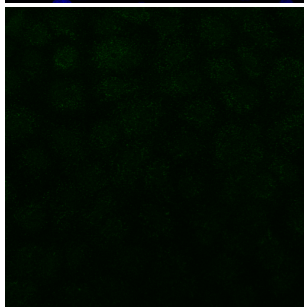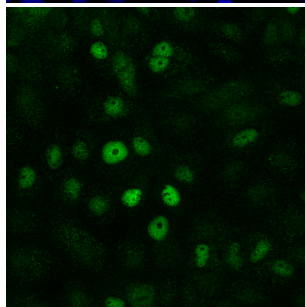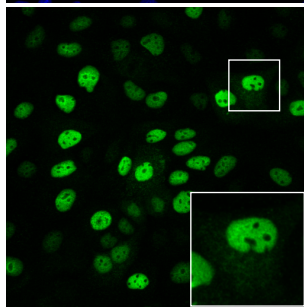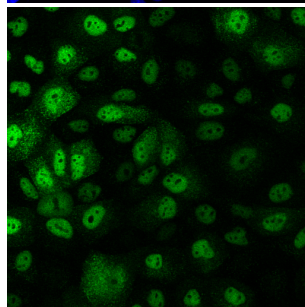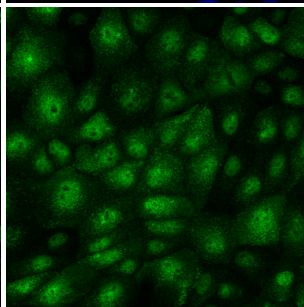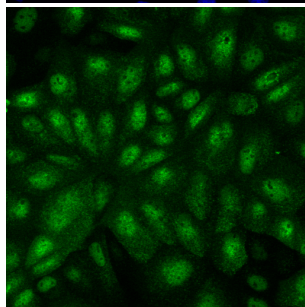

Supplement: Figure S1 — Nucleoprotein expression in MDCK cells after infection with influenza A/PR8. MDCK cells were infected at MOI 100 and incubated at 37°C for the designated time points. The cells were fixed and immunostained using monoclonal anti-NP antibodies (millipore) followed by secondary antibody staining (anti-mouse, Alexa568, life technologies). The cellular DNA was stained with DAPI. NP expression starts between 2 and 4 hrs p.i. with a strong accumulation in the nucleus. Beginning nuclear export was rarely detected at 6 hrs p.i. (see inset), but is clearly visible at later time points. (PDF) [file pone.0094257.s001.pdf]

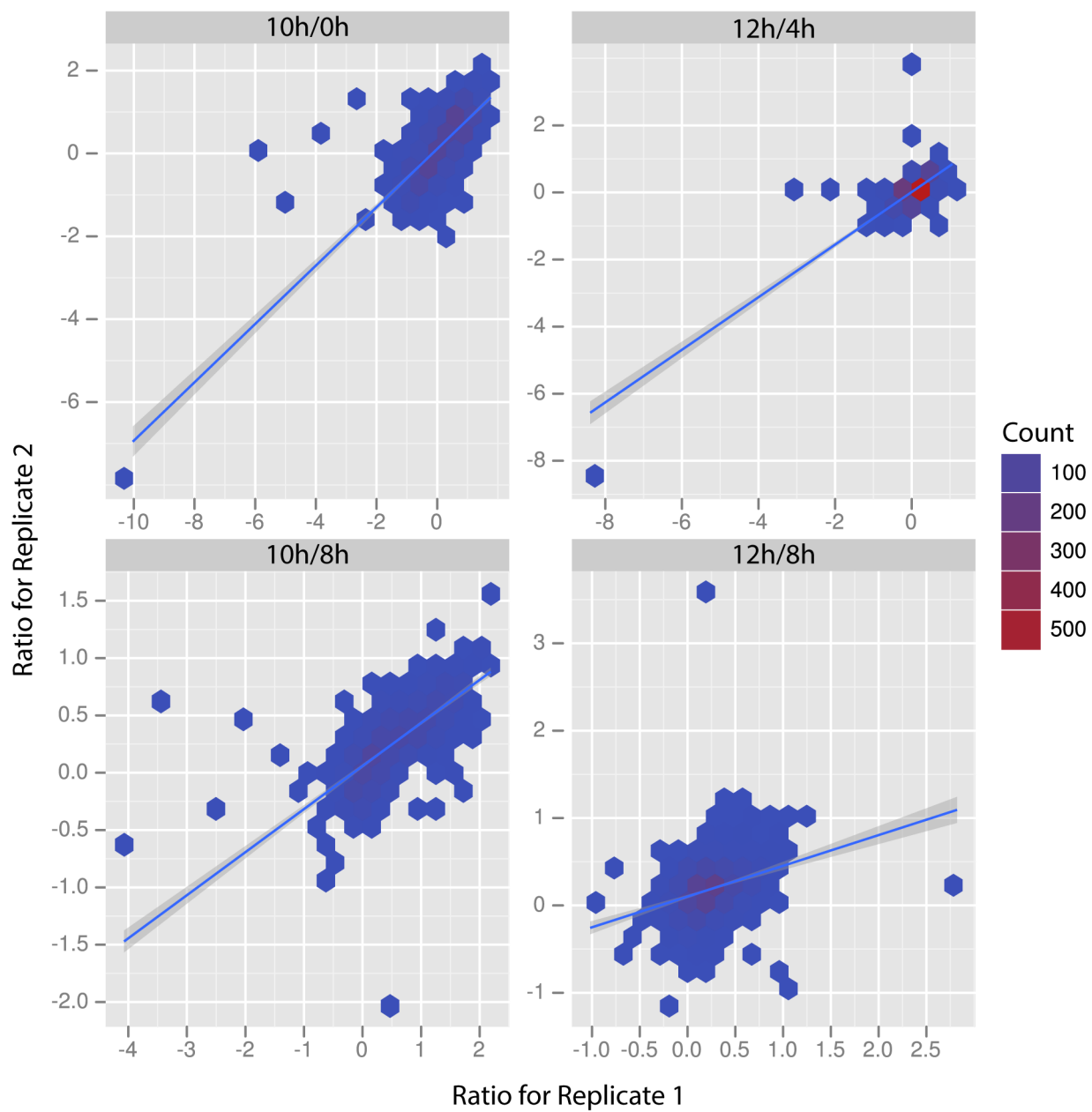

Supplement: Figure S2 — Scatterplots of the two independent replicates for all SILAC ratios over the entire time range of the experiment (0–12 hrs p.i.). The number of data points (counts) in each hexagon is color coded as indicated. Despite negligible outliners the replicates show a good correlation. (PDF) [file pone.0094257.s002.pdf]

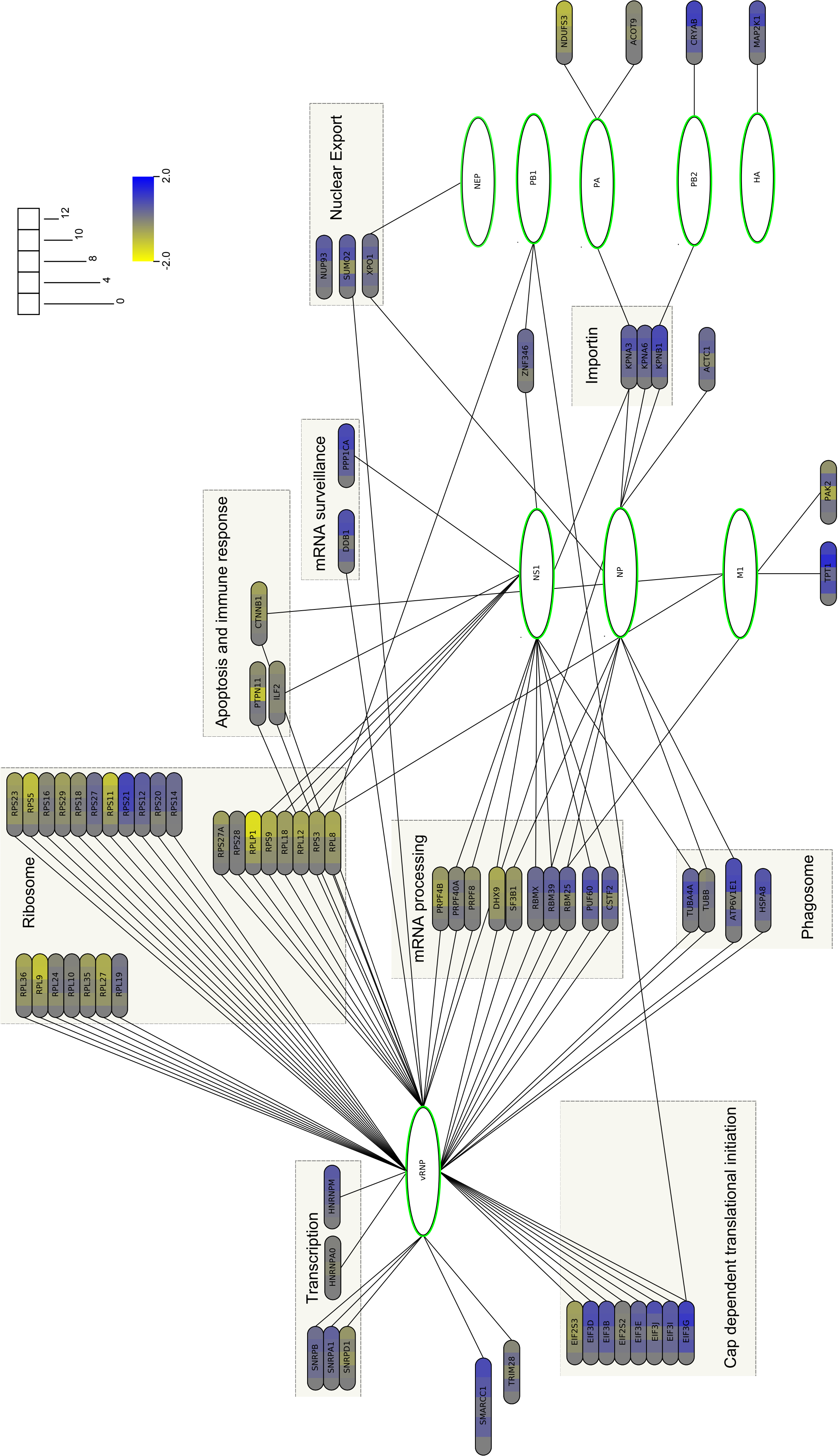

Supplement: Figure S4 — Network of viral-host interaction partners. This network is a combination of different interaction networks presented by Watanabe et al. [65] and represents the interactions between viral proteins and host proteins as well as protein interactions with the vRNPs. Colored bars for each cellular protein show the respective expression profile during 12 hrs of infection. (PDF) [file pone.0094257.s004.pdf]
