# Supplementary material for: Alteration of Protein Levels during Influenza Virus H1N1 Infection in Host Cells: A Proteomic Survey of Host and Virus Reveals Differential Dynamics
Source: PLoS One. 2014 Apr 9;9(4):e94257. doi: 10.1371/journal.pone.0094257 (PMC3981805; doi:10.1371/journal.pone.0094257)
Supplement: Figure S3 — Scheme of metabolic pathways influenced by the viral infection. Metabolic pathways that are significantly overrepresented in our data set are depicted. In general, glycolytic enzymes increase in abundance, whereas those of the TCA cycle stay constant or decline after viral infection. (PDF) [file pone.0094257.s003.pdf]

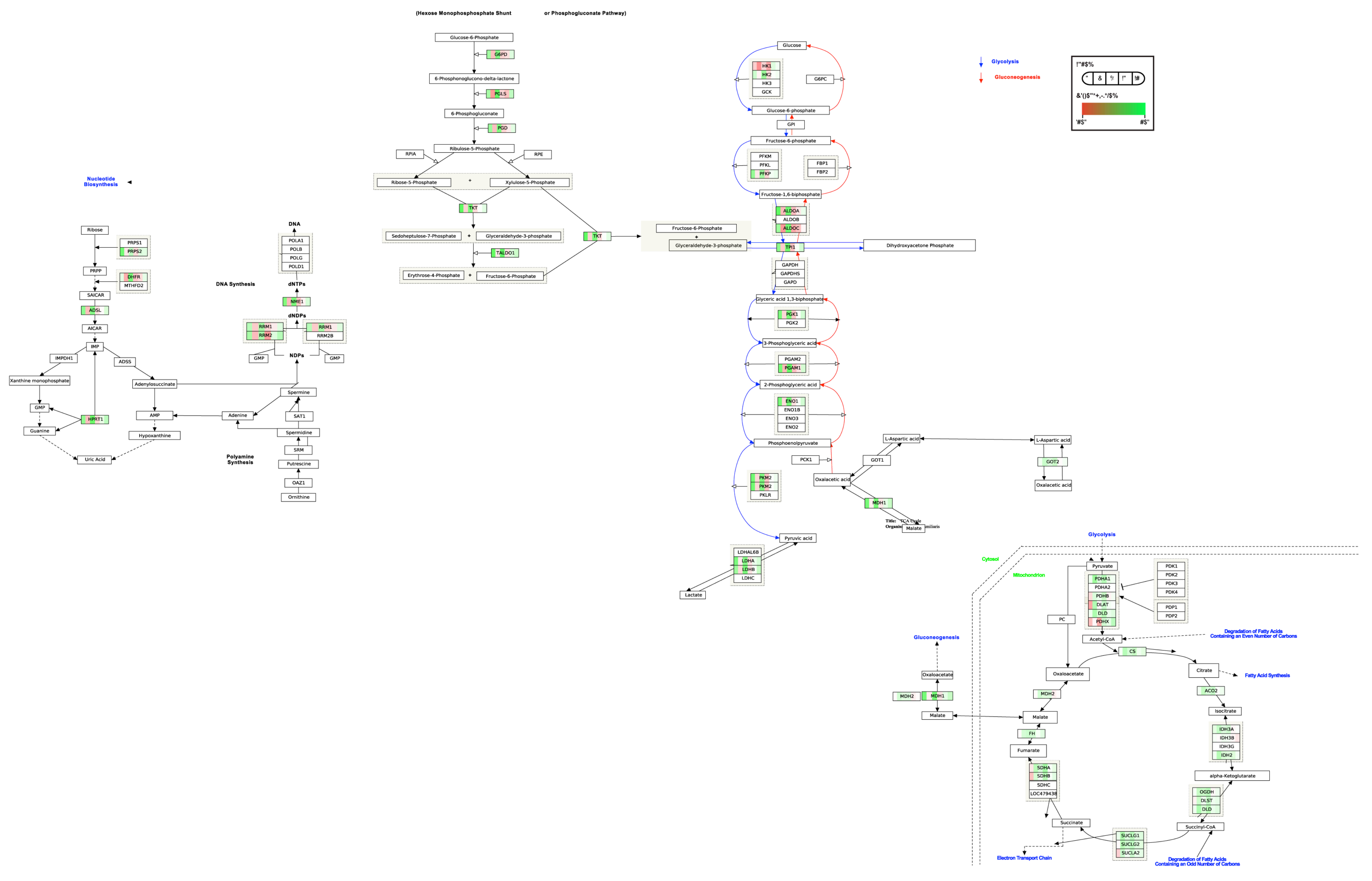
(Hexose Monophosphophate Shunt
or Phosphogluconate Pathway)
Glucose-6-Phosphate
G6PD
6-Phosphonoglucono-delta-lactone
PGLS
6-Phosphogluconate
PGD
Ribulose-5-Phosphate
RPIA
RPE
Ribose-5-Phosphate
Xylulose-5-Phosphate
TKT
Sedoheptulose-7-Phosphate
Glyceraldehyde-3-phosphate
TALDO1
Erythrose-4-Phosphate
Fructose-6-Phosphate
TKT
Fructose-6-Phosphate
Glyceraldehyde-3-phosphate
Glucose
HK1
HK2
HK3
GCK
G6PC
Glucose-6-phosphate
GPI
Fructose-6-phosphate
PFKM
PFKL
PFKP
FBP1
FBP2
Fructose-1,6-biphosphate
ALDOA
ALDOB
ALDOC
TPI1
Dihydroxyacetone Phosphate
GAPDH
GAPDHS
GAPD
Glyceric acid 1,3-biphosphate
PGK1
PGK2
3-Phosphoglyceric acid
PGAM2
PGAM1
2-Phosphoglyceric acid
ENO1
ENO1B
ENO3
ENO2
Phosphoenolpyruvate
PCK1
Oxalacetic acid
PKM2
PKM2
PKLR
Pyruvic acid
LDHAL6B
LDHA
LDHB
LDHC
Lactate
L-Aspartic acid
GOT1
L-Aspartic acid
GOT2
Oxalacetic acid
MDH1
Malate
Glycolysis
Gluconeogenesis
Nucleotide Biosynthesis
Ribose
PRPS1
PRPS2
PRPP
DHFR
MTHFD2
SAICAR
ADSL
AICAR
IMP
IMPDH1
ADSS
Xanthine monophosphate
Adenylosuccinate
GMP
HPRT1
AMP
Adenine
Guanine
Hypoxanthine
Uric Acid
DNA Synthesis
DNA
POLA1
POLB
POLG
POLD1
dNTPs
NME1
dNDPs
RRM1
RRM2
RRM1
RRM2B
GMP
GMP
NDPs
Spermine
SAT1
Spermidine
SRM
Putrescine
OAZ1
Ornithine
Polyamine Synthesis
Glycolysis
Cytosol
Mitochondrion
Pyruvate
PDHA1
PDHA2
PDHB
DLAT
DLD
PDHX
PDK1
PDK2
PDK3
PDK4
PDP1
PDP2
PC
Acetyl-CoA
Degradation of Fatty Acids Containing an Even Number of Carbons
CS
Citrate
Fatty Acid Synthesis
ACO2
Isocitrate
IDH3A
IDH3B
IDH3G
IDH2
alpha-Ketoglutarate
OGDH
DLST
DLD
Succinyl-CoA
Degradation of Fatty Acids Containing an Odd Number of Carbons
SUCLG1
SUCLG2
SUCLA2
Succinate
Electron Transport Chain
SDHA
SDHB
SDHC
LOC479438
Fumarate
FH
Malate
MDH2
Oxaloacetate
Gluconeogenesis
Oxaloacetate
MDH2
MDH1
Malate
