# Supplementary material for: Alteration of Protein Levels during Influenza Virus H1N1 Infection in Host Cells: A Proteomic Survey of Host and Virus Reveals Differential Dynamics
Source: PLoS One. 2014 Apr 9;9(4):e94257. doi: 10.1371/journal.pone.0094257 (PMC3981805; doi:10.1371/journal.pone.0094257)
Supplement: Table S1 — List of viral (green) and cellular (blue) proteins with their changes over time. (PDF) [file pone.0094257.s005.pdf]

Table S1: List of viral (green) and cellular (blue) proteins with their changes over time.

	log2 fold change				
**Symbol**	4hrs p.i.	8 hrs p.i.	10 hrs p.i.	12 hrs p.i.	Name
**P03428**	-1,39	0,52	1,74	2,01	PB2
**P03431**	-0,86	0,68	1,91	2,33	PB1
**P03433**	-0,60	1,37	2,48	2,72	PA
**P03452**	-1,32	2,21	3,73	4,10	HA
**P03466**	-1,47	2,86	4,37	4,83	NP
**P03468**	-1,80	0,70	2,38	3,07	NA
**P03485**	-1,96	2,37	4,33	4,88	M
**P03496**	-0,45	3,63	5,04	5,59	NS1
**P03508**	-0,27	0,75	2,48	2,38	NS2

		log2 fold change				
**Symbol**	**Entrez Id**	**4hrs p.i.**	**8 hrs p.i.**	**10 hrs p.i.**	**12 hrs p.i.**	**Name**
**LOC611024**	611024	-2,14	-1,87	-1,99	-2,40	similar to 3-beta-hydroxysteroid-delta(8),delta(7)-isomerase (Cholestenol delta-isomerase) (Delta8-delta7 sterol isomerase) (D8-D7 sterol isomerase) (Emopamil-binding protein)
**RPLP1**	478356	-1,25	-0,90	-1,74	-1,50	ribosomal protein, large, P1
**SFXN1**	489109	-1,47	-1,40	-1,55	-1,67	sideroflexin 1
**ELOVL1**	482533	-1,29	-1,15	-1,49	-1,49	ELOVL fatty acid elongase 1
**LOC478576**	478576	-0,79	-0,71	-1,45	-1,22	similar to ribosomal protein S15a
**PGRMC2**	476084	-1,09	-0,93	-1,44	-2,05	progesterone receptor membrane component 2
**RPL9**	479109	-0,83	-0,53	-1,44	-1,06	ribosomal protein L9
**LOC477944**	477944	-0,81	-0,74	-1,44	-1,09	similar to ubiquinol-cytochrome c reductase binding protein
**RPLP2**	475991	-0,90	-0,59	-1,42	-0,99	ribosomal protein, large, P2
**TIMM23**	486776	-1,17	-0,87	-1,42	-1,40	translocase of inner mitochondrial membrane 23 homolog (yeast)
**SSR1**	403951	-1,22	-1,24	-1,42	-1,33	signal sequence receptor, alpha
**LOC481543**	481543	-0,82	-0,68	-1,40	-1,05	similar to ribosomal protein L31
**RPS2**	479886	-0,81	-0,65	-1,39	-1,17	ribosomal protein S2
**ATP1B1**	403966	-1,34	-1,17	-1,35	-1,55	ATPase, Na+/K+ transporting, beta 1 polypeptide
**SQLE**	608021	-0,95	-0,99	-1,35	-1,74	squalene epoxidase
**FDFT1**	477362	-1,29	-1,04	-1,33	-1,74	farnesyl-diphosphate farnesyltransferase 1
**TMX1**	610791	-1,17	-1,13	-1,33	-1,41	thioredoxin-related transmembrane protein 1
**RPL30**	480751	-0,62	-0,51	-1,29	-0,90	ribosomal protein L30
**LOC479623**	479623	-1,19	-1,14	-1,29	-1,42	similar to Cytochrome c oxidase subunit IV isoform 1, mitochondrial precursor (COX IV-1) (Cytochrome c oxidase polypeptide IV)
**SPTLC1**	484192	-1,12	-1,23	-1,28	-1,56	serine palmitoyltransferase, long chain base subunit 1
**TRPV4**	486308	-1,01	-0,77	-1,27	-1,64	transient receptor potential cation channel, subfamily V, member 4
**LOC611134**	611134	-1,12	-1,09	-1,25	-1,49	similar to cytochrome c oxidase, subunit 7a 3
**LOC488258**	488258	-1,14	-1,20	-1,25	-1,63	similar to Histone H1.2 (H1d)

		log2 fold change				
**Symbol**	**Entrez Id**	**4hrs p.i.**	**8 hrs p.i.**	**10 hrs p.i.**	**12 hrs p.i.**	**Name**
**LOC476758**	476758	-1,37	-1,04	-1,25	-1,51	similar to basigin isoform 1
**ATP5F1**	479901	-1,04	-0,89	-1,24	-1,18	ATP synthase, H+ transporting, mitochondrial Fo complex, subunit B1
**VDAC1**	474681	-1,03	-0,90	-1,24	-1,20	voltage-dependent anion channel 1
**SLC25A4**	475630	-0,91	-1,00	-1,24	-1,20	solute carrier family 25 (mitochondrial carrier; adenine nucleotide translocator), member 4
**GNAI2**	442957	-0,92	-0,75	-1,24	-1,18	guanine nucleotide binding protein (G protein), alpha inhibiting activity polypeptide 2
**NOP2**	611645	-0,98	-0,60	-1,23	-1,21	NOP2 nucleolar protein homolog (yeast)
**VDAC2**	479255	-1,05	-0,87	-1,23	-1,14	voltage-dependent anion channel 2
**NDUFA5**	475214	-0,94	-1,09	-1,22	-1,43	NADH dehydrogenase (ubiquinone) 1 alpha subcomplex, 5, 13kDa
**LOC610942**	610942	-0,90	-0,87	-1,22	-1,23	similar to cytochrome b5 outer mitochondrial membrane precursor
**FAF2**	489102	-1,25	-1,05	-1,21	-1,14	Fas associated factor family member 2
**RPS11**	403684	-0,62	-0,64	-1,20	-1,06	ribosomal protein S11
**STT3A**	489300	-0,87	-0,53	-1,19	-1,09	STT3, subunit of the oligosaccharyltransferase complex, homolog A (S. cerevisiae)
**LOC477031**	477031	-0,77	-0,58	-1,19	-1,06	similar to ribosomal protein L14
**CANX**	403908	-1,07	-0,99	-1,19	-1,30	calnexin
**RPS5**	476366	-0,83	-0,66	-1,19	-1,00	ribosomal protein S5
**LARP1**	612929	-0,68	-0,74	-1,18	-1,09	La ribonucleoprotein domain family, member 1
**AGK**	482764	-1,20	-1,05	-1,17	-1,36	acylglycerol kinase
**TOMM40**	611055	-1,13	-1,05	-1,17	-1,33	translocase of outer mitochondrial membrane 40 homolog (yeast)
**RPS25**	479412	-0,62	-0,53	-1,16	-0,86	ribosomal protein S25
**SSR3**	477124	-1,10	-0,77	-1,16	-1,32	signal sequence receptor, gamma (translocon-associated protein gamma)
**LOC476637**	476637	-0,55	-0,39	-1,16	-0,79	similar to ribosomal protein L18a
**RPL6**	403691	-0,65	-0,46	-1,16	-0,91	Ribosomal protein L6
**CPT1A**	403583	-1,16	-0,90	-1,15	-1,33	carnitine palmitoyltransferase 1A (liver)
**AGPS**	488421	-0,92	-0,92	-1,15	-1,01	alkylglycerone phosphate synthase
**RPL15**	477046	-0,60	-0,37	-1,14	-0,82	ribosomal protein L15
**YBX1**	479011	-0,56	-0,48	-1,14	-0,93	Y box binding protein 1
**RPS8**	475381	-0,63	-0,47	-1,14	-0,80	ribosomal protein S8
**CISD2**	610084	-1,02	-1,04	-1,14	-1,22	CDGSH iron sulfur domain 2
**RPN1**	476516	-1,07	-0,85	-1,13	-1,13	ribophorin I
**RRP12**	608725	-1,14	-0,57	-1,13	-1,27	ribosomal RNA processing 12 homolog (S. cerevisiae)
**SLC3A2**	483777	-1,03	-0,78	-1,12	-1,21	solute carrier family 3 (activators of dibasic and neutral amino acid transport), member 2
**UBTF**	480503	-0,52	-0,34	-1,12	-1,34	upstream binding transcription factor, RNA polymerase I
**LOC488273**	488273	-0,81	-0,82	-1,12	-1,28	similar to Histone H1.5 (Histone H1a)
**LOC489372**	489372	-1,22	-1,07	-1,11	-1,20	similar to Histone H2A.x

		log2 fold change				
**Symbol**	**Entrez Id**	**4hrs p.i.**	**8 hrs p.i.**	**10 hrs p.i.**	**12 hrs p.i.**	**Name**
						(H2a/x)
**GPD2**	478755	-0,97	-0,86	-1,11	-1,22	glycerol-3-phosphate dehydrogenase 2 (mitochondrial)
**RPN2**	477223	-1,10	-0,78	-1,11	-1,05	ribophorin II
**SPTLC2**	480403	-0,67	-0,86	-1,10	-1,18	serine palmitoyltransferase, long chain base subunit 2
**RPS9**	476387	-0,49	-0,35	-1,10	-0,74	ribosomal protein S9
**RPL35A**	478597	-0,57	-0,45	-1,09	-0,79	ribosomal protein L35a
**RPL7**	477912	-0,55	-0,40	-1,09	-0,80	ribosomal protein L7
**CAV1**	403980	-0,80	-1,05	-1,06	-1,35	caveolin 1, caveolae protein, 22kDa
**TMEM43**	484637	-0,75	-0,82	-1,06	-1,02	transmembrane protein 43
**NDUFA10**	608244	-1,08	-1,01	-1,06	-1,17	NADH dehydrogenase (ubiquinone) 1 alpha subcomplex, 10, 42kDa
**RPL7A**	403678	-0,57	-0,44	-1,05	-0,88	Ribosomal protein L7a
**TIMM50**	476461	-1,09	-1,04	-1,05	-1,25	translocase of inner mitochondrial membrane 50 homolog (S. cerevisiae)
**RPS4Y1**	403893	-0,62	-0,47	-1,05	-0,82	ribosomal protein S4, Y-linked 1
**IMMT**	475764	-0,91	-0,78	-1,04	-1,10	inner membrane protein, mitochondrial
**VDAC3**	475462	-0,70	-0,70	-1,02	-0,94	voltage-dependent anion channel 3
**CYP1B1**	483038	-0,49	-0,60	-1,02	-1,30	cytochrome P450, family 1, subfamily B, polypeptide 1
**LOC474504**	474504	-0,47	-0,31	-1,02	-0,66	similar to 60S ribosomal protein L3 (L4)
**ERLIN2**	607518	-0,89	-0,81	-1,01	-1,05	ER lipid raft associated 2
**FLT3LG**	442938	-0,47	-0,38	-1,01	-0,65	fms-related tyrosine kinase 3 ligand
**LOC475225**	475225	-0,82	-0,81	-1,00	-1,15	similar to Cytochrome P450 51A1 (CYPLI) (P450LI) (Sterol 14-alpha demethylase) (Lanosterol 14-alpha demethylase) (LDM) (P450-14DM) (P45014DM)
**RPS15**	476753	-0,57	-0,44	-1,00	-0,89	ribosomal protein S15
**OCIAD1**	475140	-1,02	-1,03	-0,99	-1,26	OCIA domain containing 1
**RPL8**	475130	-0,51	-0,25	-0,99	-0,64	ribosomal protein L8
**ITGB1**	477956	-0,69	-0,62	-0,99	-0,99	integrin, beta 1 (fibronectin receptor, beta polypeptide, antigen CD29 includes MDF2, MSK12)
**SLC25A6**	480830	-0,65	-0,73	-0,99	-0,88	solute carrier family 25 (mitochondrial carrier; adenine nucleotide translocator), member 6
**LETM1**	488807	-0,85	-0,86	-0,98	-0,83	leucine zipper-EF-hand containing transmembrane protein 1
**ETFDH**	475480	-0,81	-0,84	-0,98	-1,19	electron-transferring-flavoprotein dehydrogenase
**SLC4A7**	485637	-1,15	-0,80	-0,97	-1,07	solute carrier family 4, sodium bicarbonate cotransporter, member 7
**PODXL**	482252	-0,80	-0,89	-0,97	-1,21	podocalyxin-like
**LOC474656**	474656	-0,38	-0,25	-0,97	-0,65	similar to ribosomal protein L10a
**LOC480235**	480235	-0,47	-0,30	-0,97	-0,67	similar to ribosomal protein S13
**LOC479261**	479261	-0,64	-0,90	-0,97	-1,15	similar to R53.5
**DPM1**	477264	-0,69	-0,86	-0,97	-1,02	dolichyl-phosphate mannosyltransferase polypeptide 1, catalytic subunit
**ATP1A1**	403992	-0,87	-0,70	-0,97	-0,95	ATPase, Na+/K+

		log2 fold change				
Symbol	Entrez Id	4hrs p.i.	8 hrs p.i.	10 hrs p.i.	12 hrs p.i.	Name
						transporting, alpha 1 polypeptide
**DBT**	479929	-0,48	-0,52	-0,96	-0,96	dihydrolipoamide branched chain transacylase E2
**LOC479350**	479350	-0,38	-0,50	-0,96	-0,85	similar to ribosomal protein L37
**TMED5**	479947	-0,66	-0,47	-0,96	-0,71	transmembrane emp24 protein transport domain containing 5
**NDUFS3**	475978	-0,73	-0,53	-0,96	-0,92	NADH dehydrogenase (ubiquinone) Fe-S protein 3, 30kDa (NADH-coenzyme Q reductase)
**RPL22**	479587	-0,46	-0,28	-0,96	-0,60	ribosomal protein L22
**NDUFA9**	477718	-0,57	-0,81	-0,96	-0,92	NADH dehydrogenase (ubiquinone) 1 alpha subcomplex, 9, 39kDa
**ATP5C1**	478009	-0,73	-0,78	-0,95	-0,92	ATP synthase, H+ transporting, mitochondrial F1 complex, gamma polypeptide 1
**RAB14**	474818	-0,79	-0,88	-0,95	-0,86	RAB14, member RAS oncogene family
**RPL27**	403688	-0,57	-0,41	-0,95	-0,70	ribosomal protein L27
**SLC1A5**	484425	-1,91	-1,20	-0,94	-1,46	solute carrier family 1 (neutral amino acid transporter), member 5
**ITGB3**	403788	-0,54	-0,52	-0,93	-0,91	integrin, beta 3 (platelet glycoprotein IIIa, antigen CD61)
**RPL12**	476346	-0,49	-0,34	-0,93	-0,60	ribosomal protein L12
**SLC25A3**	475436	-0,71	-0,64	-0,93	-0,84	solute carrier family 25 (mitochondrial carrier; phosphate carrier), member 3
**SLC25A24**	611922	-0,74	-0,79	-0,93	-1,00	solute carrier family 25 (mitochondrial carrier; phosphate carrier), member 24
**YTHDF2**	478161	-0,33	-0,08	-0,93	-0,72	YTH domain family, member 2
**LOC476372**	476372	-0,87	-0,84	-0,93	-0,98	similar to ATP synthase, H+ transporting, mitochondrial F0 complex, subunit f, isoform 2
**NOL10**	482966	-0,93	-0,79	-0,92	-1,11	nucleolar protein 10
**F11R**	478974	-1,15	-1,23	-0,92	-1,27	F11 receptor
**SLC25A1**	608348	-0,61	-0,77	-0,91	-0,94	solute carrier family 25 (mitochondrial carrier; citrate transporter), member 1
**RPL36**	476738	-0,37	-0,23	-0,90	-0,62	ribosomal protein L36
**MCU**	489042	-0,86	-0,78	-0,90	-1,02	mitochondrial calcium uniporter
**LOC474433**	474433	-0,88	-0,79	-0,90	-1,01	similar to ATP synthase, H+ transporting, mitochondrial F0 complex, subunit d isoform a
**LOC476525**	476525	-0,42	-0,25	-0,90	-0,59	similar to 60S ribosomal protein L26 (Silica-induced gene 20 protein) (SIG-20)
**SEC61G**	404017	-0,69	-0,64	-0,89	-0,98	Sec61 gamma subunit
**SLC25A13**	610138	-0,93	-0,73	-0,89	-1,03	solute carrier family 25, member 13 (citrin)
**ATP2A2**	403878	-0,69	-0,77	-0,89	-0,89	ATPase, Ca++ transporting, cardiac muscle, slow twitch 2
**PON2**	403855	-0,67	0,06	-0,88	-0,63	paraoxonase 2
**COX5B**	474567	-0,74	-0,82	-0,87	-1,16	cytochrome c oxidase polypeptide Vb
**MRPS35**	477660	-0,59	-0,67	-0,87	-0,82	mitochondrial ribosomal protein S35
**TRIM25**	491103	-0,57	-0,76	-0,87	-0,95	tripartite motif containing 25

Symbol	Entrez Id	log2 fold change 4hrs p.i.	8 hrs p.i.	10 hrs p.i.	12 hrs p.i.	Name
**HSD17B12**	475939	-0,72	-0,72	-0,86	-0,94	hydroxysteroid (17-beta) dehydrogenase 12
**IGF2BP1**	491064	-0,46	-0,48	-0,86	-0,75	insulin-like growth factor 2 mRNA binding protein 1
**MRPL49**	483747	-0,53	-0,71	-0,86	-1,02	mitochondrial ribosomal protein L49
**RPS23**	479159	-0,31	-0,27	-0,86	-0,56	ribosomal protein S23
**LOC479257**	479257	-0,28	-0,36	-0,86	-0,60	similar to ribosomal protein S24 isoform 3
**SSR4**	481082	-0,63	-0,48	-0,86	-0,63	signal sequence receptor, delta
**RPS3A**	403677	-0,25	-0,18	-0,86	-0,50	ribosomal protein S3A
**STOML2**	474755	-1,07	-0,89	-0,86	-1,12	stomatin (EPB72)-like 2
**NDUFS1**	478880	-0,79	-0,72	-0,85	-0,83	NADH dehydrogenase (ubiquinone) Fe-S protein 1, 75kDa (NADH-coenzyme Q reductase)
**LOC488264**	488264	-0,44	-0,74	-0,85	-0,87	similar to Histone H1.2 (H1d)
**ITGAV**	488437	-0,72	-0,77	-0,85	-1,00	integrin, alpha V (vitronectin receptor, alpha polypeptide, antigen CD51)
**TSPAN8**	474448	-0,53	-0,74	-0,84	-0,76	tetraspanin 8
**AIFM1**	481048	-0,86	-0,80	-0,84	-0,91	apoptosis-inducing factor, mitochondrion-associated, 1
**ATL3**	476044	-0,75	-0,54	-0,84	-0,84	atlastin GTPase 3
**LOC474877**	474877	-0,37	-0,36	-0,84	-0,58	similar to 40S ribosomal protein S10
**CEACAM28**	484473	-0,46	-0,92	-0,84	-0,90	carcinoembryonic antigen-related cell adhesion molecule 28
**CYB5R3**	474479	-0,75	-0,68	-0,84	-0,76	cytochrome b5 reductase 3
**LOC475410**	475410	-0,24	-0,22	-0,83	-0,53	similar to 60S ribosomal protein L32
**ITGB4**	483318	-0,55	-0,87	-0,83	-1,37	integrin, beta 4
**RPS29**	610596	-0,24	-0,31	-0,83	-0,52	ribosomal protein S29
**MPDU1**	489477	-0,87	-0,65	-0,83	-1,17	mannose-P-dolichol utilization defect 1
**TMCO1**	478992	-1,05	-1,01	-0,82	-1,07	transmembrane and coiled-coil domains 1
**BCAP31**	481080	-0,75	-0,69	-0,82	-0,98	B-cell receptor-associated protein 31
**TSPO**	474475	-1,09	-0,74	-0,82	-0,99	translocator protein (18kDa)
**FKBP11**	608951	-0,73	-0,56	-0,81	-0,66	FK506 binding protein 11, 19 kDa
**NDUFA13**	476659	-0,62	-0,60	-0,81	-0,89	NADH dehydrogenase (ubiquinone) 1 alpha subcomplex, 13
**ACSL4**	481018	-1,08	-0,94	-0,81	-1,18	acyl-CoA synthetase long-chain family member 4
**HMGB3**	481073	-0,47	-1,03	-0,80	-1,08	high mobility group box 3
**NOMO2**	489992	-0,73	-0,89	-0,80	-1,05	NODAL modulator 2
**PABPC4**	482464	-0,55	-0,50	-0,80	-0,70	poly(A) binding protein, cytoplasmic 4 (inducible form)
**PHB**	480547	-0,74	-0,68	-0,80	-0,90	prohibitin
**SYNCRIP**	474986	-0,48	-0,40	-0,80	-0,72	synaptotagmin binding, cytoplasmic RNA interacting protein
**RALY**	485845	-0,62	-0,34	-0,79	-0,41	RNA binding protein, autoantigenic (hnRNP-associated with lethal yellow homolog (mouse))
**LOC483166**	483166	-0,48	-0,56	-0,78	-0,72	similar to germinal histone H4 gene
**DDOST**	404012	-0,71	-0,65	-0,78	-0,76	dolichyl-diphosphooligosaccharide--protein glycosyltransferase
**PTGS2**	442942	-0,27	-0,37	-0,77	-0,52	prostaglandin-endoperoxide synthase 2 (prostaglandin G/H synthase and

		log2 fold change				
Symbol	Entrez Id	4hrs p.i.	8 hrs p.i.	10 hrs p.i.	12 hrs p.i.	Name
						cyclooxygenase)
**RPL18**	476422	-0,31	-0,18	-0,77	-0,49	ribosomal protein L18
**HSD17B4**	474630	-0,61	-0,55	-0,77	-0,82	hydroxysteroid (17-beta) dehydrogenase 4
**CIRBP**	476755	-0,27	-0,55	-0,77	-0,65	cold inducible RNA binding protein
**H2AFY**	481514	-0,66	-0,64	-0,77	-0,84	H2A histone family, member Y
**NCL**	608445	-0,40	-0,14	-0,77	-0,49	nucleolin
**TMED10**	610559	-0,77	-0,64	-0,76	-0,76	transmembrane emp24-like trafficking protein 10 (yeast)
**LOC608089**	608089	-1,14	0,33	-0,76	-1,08	similar to Adipocyte plasma membrane-associated protein (BSCv protein)
**ATP5D**	476756	-0,79	-0,82	-0,76	-0,85	ATP synthase, H+ transporting, mitochondrial F1 complex, delta subunit
**LOC491074**	491074	-0,73	-0,88	-0,76	-0,67	similar to integrin alpha 3 isoform b, precursor
**DLAT**	489406	-0,68	-0,68	-0,76	-0,69	dihydrolipoamide S-acetyltransferase
**RPS3**	476804	-0,32	-0,21	-0,75	-0,57	ribosomal protein S3
**ITGA2**	489208	-0,28	-0,70	-0,75	-0,73	integrin, alpha 2 (CD49B, alpha 2 subunit of VLA-2 receptor)
**TOMM22**	610117	-1,08	-0,90	-0,75	-1,23	translocase of outer mitochondrial membrane 22 homolog (yeast)
**DDX21**	476891	-0,61	-0,38	-0,74	-0,70	DEAD (Asp-Glu-Ala-Asp) box polypeptide 21
**LOC476842**	476842	-0,12	-0,02	-0,74	-0,38	similar to ribosomal protein L27a
**TFRC**	403703	-0,35	-0,73	-0,74	-1,02	transferrin receptor (p90, CD71)
**FIS1**	479728	-0,66	-0,72	-0,74	-0,85	fission 1 (mitochondrial outer membrane) homolog (S. cerevisiae)
**HNRNPR**	478192	-0,57	-0,49	-0,74	-0,75	heterogeneous nuclear ribonucleoprotein R
**LOC483168**	483168	-0,61	-0,77	-0,74	-0,82	similar to Histone H2A.o (H2A/o) (H2A.2) (H2a-615)
**PGRMC1**	481029	-0,64	-0,73	-0,73	-0,88	progesterone receptor membrane component 1
**RPS17**	403681	-0,19	-0,22	-0,73	-0,42	ribosomal protein S17
**RAP1B**	608981	-0,65	-0,67	-0,73	-0,68	RAP1B, member of RAS oncogene family
**TMEM33**	612937	-0,71	-0,58	-0,73	-0,68	transmembrane protein 33
**GTPBP4**	478021	-0,57	-0,23	-0,73	-0,66	GTP binding protein 4
**LOC478370**	478370	-0,68	-0,61	-0,73	-0,77	similar to Cytochrome c oxidase polypeptide Va, mitochondrial precursor
**SLC25A5**	492093	-0,27	-0,47	-0,73	-0,56	solute carrier family 25 (mitochondrial carrier; adenine nucleotide translocator), member 5
**SUPT16H**	612874	-0,67	-0,59	-0,72	-0,91	suppressor of Ty 16 homolog (S. cerevisiae)
**LOC611089**	611089	-0,51	-0,60	-0,72	-0,75	similar to H2B histone family, member F
**LOC479778**	479778	-0,42	-0,51	-0,71	-0,87	similar to fusion (involved in t(12;16) in malignant liposarcoma) (predicted)
**ATP1B3**	477098	-0,76	-0,69	-0,71	-0,81	ATPase, Na+/K+ transporting, beta 3 polypeptide
**LOC476824**	476824	-0,49	-0,40	-0,71	-0,55	similar to NADH-ubiquinone oxidoreductase 23 kDa subunit, mitochondrial precursor (Complex I-23KD) (CI-23KD) (TYKY subunit)
**L1CAM**	492244	-0,41	-0,59	-0,70	-0,83	L1 cell adhesion molecule

		log2 fold change				
**Symbol**	**Entrez Id**	**4hrs p.i.**	**8 hrs p.i.**	**10 hrs p.i.**	**12 hrs p.i.**	**Name**
**NDUFV2**	480204	-0,69	-0,60	-0,70	-0,82	NADH dehydrogenase (ubiquinone) flavoprotein 2, 24kDa
**LOC483167**	483167	-0,41	-0,50	-0,69	-0,71	similar to H3 histone, family 2 isoform 2
**EIF2S3**	480866	-0,37	-0,52	-0,69	-0,54	eukaryotic translation initiation factor 2, subunit 3 gamma, 52kDa
**LRPPRC**	474572	-0,58	-0,69	-0,69	-0,77	leucine-rich PPR-motif containing
**CISD1**	479212	-0,86	-0,85	-0,68	-0,66	CDGSH iron sulfur domain 1
**PFDN2**	488648	-0,47	-1,04	-0,68	-0,54	prefoldin subunit 2
**UPF1**	476664	-0,30	-0,49	-0,68	-1,00	UPF1 regulator of nonsense transcripts homolog (yeast)
**PABPC1**	612751	-0,25	-0,41	-0,67	-0,58	poly(A) binding protein, cytoplasmic 1
**POR**	489816	-1,00	-0,59	-0,67	-0,85	P450 (cytochrome) oxidoreductase
**NDUFB10**	479887	-0,43	-0,35	-0,67	-0,54	NADH dehydrogenase (ubiquinone) 1 beta subcomplex, 10, 22kDa
**CTNND1**	483489	-0,36	-0,53	-0,66	-0,72	catenin (cadherin-associated protein), delta 1
**LOC475121**	475121	-0,73	-0,55	-0,66	-0,69	similar to cytochrome c-1
**LOC484627**	484627	-0,25	-0,21	-0,66	-0,45	similar to H1 histone family, member X
**REEP5**	479137	-0,41	-0,76	-0,66	-0,69	receptor accessory protein 5
**RPL35**	480729	-0,15	-0,14	-0,66	-0,49	ribosomal protein L35
**MYO1D**	491153	-0,27	-0,47	-0,66	-0,81	myosin ID
**DHX9**	480032	-0,50	-0,44	-0,66	-0,67	DEAH (Asp-Glu-Ala-His) box polypeptide 9
**ZMPSTE24**	482460	-0,76	-0,86	-0,66	-0,70	zinc metallopeptidase (STE24 homolog, S. cerevisiae)
**EPCAM**	481360	-0,60	-0,31	-0,65	-0,45	epithelial cell adhesion molecule
**PICALM**	476780	-0,22	-0,37	-0,65	-0,58	phosphatidylinositol binding clathrin assembly protein
**LOC478410**	478410	-0,49	-0,51	-0,65	-0,69	similar to ATP synthase O subunit, mitochondrial precursor (Oligomycin sensitivity conferral protein) (OSCP)
**CNP**	607694	-0,49	-0,76	-0,65	-0,82	2',3'-cyclic nucleotide 3' phosphodiesterase
**RTN4**	474598	-0,78	-0,63	-0,65	-0,90	reticulon 4
**LOC488289**	488289	-0,53	-0,51	-0,63	-0,69	similar to Histone H2A.1
**SYPL1**	475889	-0,24	-0,51	-0,63	-0,72	synaptophysin-like 1
**PDHX**	475942	-0,49	0,16	-0,63	-0,98	pyruvate dehydrogenase complex, component X
**STT3B**	485628	-0,50	-0,26	-0,63	-0,55	STT3, subunit of the oligosaccharyltransferase complex, homolog B (S. cerevisiae)
**MRTO4**	487406	-0,52	-0,49	-0,62	-0,65	mRNA turnover 4 homolog (S. cerevisiae)
**YES1**	490532	-0,51	-0,44	-0,62	-0,94	v-yes-1 Yamaguchi sarcoma viral oncogene homolog 1
**MRPL12**	608030	-0,44	-0,52	-0,62	-0,60	mitochondrial ribosomal protein L12
**ARL6IP5**	476559	-0,62	-0,73	-0,62	-0,77	ADP-ribosylation-like factor 6 interacting protein 5
**SLC2A1**	482437	-0,06	-0,18	-0,62	-0,43	solute carrier family 2 (facilitated glucose transporter), member 1
**LOC612644**	612644	-0,57	-0,79	-0,62	-0,90	similar to cytochrome c oxidase, subunit VIb polypeptide 1
**STOM**	612719	-0,51	-0,33	-0,61	-0,61	stomatin
**ESYT1**	474397	-0,66	-0,78	-0,61	-0,96	extended synaptotagmin-like protein 1

		log2 fold change				
**Symbol**	**Entrez Id**	**4hrs p.i.**	**8 hrs p.i.**	**10 hrs p.i.**	**12 hrs p.i.**	**Name**
**SRPK1**	474887	-0,22	0,07	-0,61	-0,33	SRSF protein kinase 1
**RAB2A**	404009	-0,42	-0,56	-0,61	-0,63	RAB2A, member RAS oncogene family
**ABCD3**	479939	-0,49	-0,36	-0,61	-0,59	ATP-binding cassette, sub-family D (ALD), member 3
**BMS1**	477760	-0,25	-0,24	-0,61	-0,58	BMS1 homolog, ribosome assembly protein (yeast)
**SRSF9**	477510	-0,26	-0,42	-0,60	-0,66	serine/arginine-rich splicing factor 9
**VAPA**	480208	-0,43	-0,38	-0,60	-0,66	VAMP (vesicle-associated membrane protein)-associated protein A, 33kDa
**PARP1**	490385	-0,50	-0,61	-0,60	-0,60	poly (ADP-ribose) polymerase 1
**ARL8B**	612944	-0,44	-0,40	-0,59	-0,04	ADP-ribosylation factor-like 8B
**PRPF4B**	488199	-0,29	-0,65	-0,59	-0,39	PRP4 pre-mRNA processing factor 4 homolog B (yeast)
**DDX55**	486246	-0,28	-0,16	-0,59	-0,29	DEAD (Asp-Glu-Ala-Asp) box polypeptide 55
**LOC475524**	475524	-0,73	-0,60	-0,59	-0,58	similar to single-stranded DNA binding protein 1 isoform 1
**CSNK2A1**	477185	-0,68	-0,88	-0,59	-0,68	casein kinase 2, alpha 1 polypeptide
**PCYOX1**	481418	-0,61	-0,64	-0,59	-0,65	prenylcysteine oxidase 1
**ITPR3**	481746	-0,43	-0,81	-0,58	-0,90	inositol 1,4,5-trisphosphate receptor, type 3
**RPS27A**	474599	0,05	-0,27	-0,58	-0,49	ribosomal protein S27a
**LAMP2**	481037	-0,28	-0,77	-0,58	-0,40	lysosomal-associated membrane protein 2
**NPM3**	477801	0,46	0,29	-0,57	-0,78	nucleophosmin/nucleoplasmin 3
**LOC478509**	478509	0,00	-0,12	-0,57	-0,33	similar to ribosomal protein L34
**INTS2**	610121	0,24	0,29	-0,57	0,14	integrator complex subunit 2
**LOC475501**	475501	-0,42	-0,52	-0,57	-0,41	similar to H2A histone family, member V isoform 5
**PTBP1**	485097	-0,24	-0,24	-0,56	-0,36	polypyrimidine tract binding protein 1
**RAB1B**	610762	-0,29	-0,27	-0,56	-0,34	RAB1B, member RAS oncogene family
**RALA**	475875	-0,25	-0,45	-0,56	-0,46	v-ral simian leukemia viral oncogene homolog A (ras related)
**RPS18**	403685	-0,01	0,04	-0,56	-0,30	ribosomal protein S18
**MYBBP1A**	489442	-0,52	-0,23	-0,56	-0,49	MYB binding protein (P160) 1a
**MRPS26**	477175	-1,02	-0,92	-0,56	-0,98	mitochondrial ribosomal protein S26
**GNB1**	403912	-0,41	-0,28	-0,55	-0,44	guanine nucleotide binding protein (G protein), beta polypeptide 1
**RPL37A**	478904	0,25	0,14	-0,55	-0,12	ribosomal protein L37a
**RAB10**	403958	-0,39	-0,32	-0,55	-0,42	RAB10, member RAS oncogene family
**RAB7A**	404007	-0,35	-0,51	-0,54	-0,59	RAB7A, member RAS oncogene family
**WDR75**	488443	-0,33	-0,30	-0,54	-0,66	WD repeat domain 75
**HEATR1**	479192	-0,46	-0,32	-0,54	-0,48	HEAT repeat containing 1
**CLU**	442971	-0,23	-0,21	-0,54	-0,52	clusterin
**RSL1D1**	479847	-0,45	-0,20	-0,53	-0,59	ribosomal L1 domain containing 1
**CHCHD3**	607574	-0,16	0,03	-0,53	-0,21	coiled-coil-helix-coiled-coil-helix domain containing 3
**RBM4**	610648	-0,33	-0,07	-0,53	-0,56	RNA binding motif protein 4
**LOC490458**	490458	-0,37	-0,64	-0,53	-0,81	similar to S100 calcium binding protein A16
**LOC483175**	483175	-0,31	-0,55	-0,53	-0,61	similar to histone H2A
**NDUFV1**	476004	-0,60	-0,58	-0,53	-0,62	NADH dehydrogenase (ubiquinone) flavoprotein 1,

Symbol	Entrez Id	log2 fold change 4hrs p.i.	8 hrs p.i.	10 hrs p.i.	12 hrs p.i.	Name
						51kDa
**HMGB2**	486068	-0,04	-0,52	-0,52	-0,70	high mobility group box 2
**UQCRC2**	479815	-0,47	-0,49	-0,52	-0,68	ubiquinol-cytochrome c reductase core protein II
**ENTPD3**	485604	-0,31	-0,74	-0,52	-0,76	ectonucleoside triphosphate diphosphohydrolase 3
**FKBP10**	490975	-0,59	-1,11	-0,52	-0,96	FK506 binding protein 10, 65 kDa
**CSDE1**	475807	-0,11	-0,24	-0,52	-0,42	cold shock domain containing E1, RNA-binding
**MYO1C**	480646	-0,53	-0,51	-0,52	-0,63	myosin IC
**WDR43**	475713	-0,42	-0,95	-0,52	-0,75	WD repeat domain 43
**SEC61B**	404018	-0,32	-0,61	-0,52	-0,21	Sec61 beta subunit
**ATP5B**	403669	-0,58	-0,59	-0,52	-0,57	ATP synthase, H+ transporting, mitochondrial F1 complex, beta polypeptide
**LOC481774**	481774	-0,12	-0,25	-0,52	-0,49	similar to U1 small nuclear ribonucleoprotein C (U1 snRNP protein C) (U1C protein) (U1-C)
**COPA**	478973	-0,19	-0,36	-0,51	-0,52	coatomer protein complex, subunit alpha
**ATAD3B**	479568	-0,46	-0,35	-0,51	-0,43	ATPase family, AAA domain containing 3B
**RAB1A**	403774	-0,43	-0,36	-0,51	-0,48	RAB1A, member RAS oncogene family
**ILF3**	484950	-0,28	-0,30	-0,51	-0,52	interleukin enhancer binding factor 3, 90kDa
**EPHA2**	487421	-0,28	-0,74	-0,51	-0,36	EPH receptor A2
**RRS1**	486978	-0,60	-0,39	-0,50	-0,62	RRS1 ribosome biogenesis regulator homolog (S. cerevisiae)
**BCKDHB**	474978	-0,40	-0,54	-0,50	-0,71	branched chain keto acid dehydrogenase E1, beta polypeptide
**TRA2B**	478663	-0,29	-0,37	-0,50	-0,56	transformer 2 beta homolog (Drosophila)
**RAB6A**	608330	-0,35	-0,34	-0,50	-0,46	RAB6A, member RAS oncogene family
**AFG3L2**	490560	-0,40	0,37	-0,50	-0,37	AFG3 ATPase family gene 3-like 2 (S. cerevisiae)
**SDHB**	478217	-0,47	-0,55	-0,50	-0,67	succinate dehydrogenase complex, subunit B, iron sulfur (Ip)
**CELF1**	475979	-0,13	-0,52	-0,49	-0,80	CUGBP, Elav-like family member 1
**LOC478348**	478348	-0,06	-0,02	-0,49	-0,20	similar to ribosomal protein L4
**ERP29**	477482	-0,45	-0,83	-0,48	-0,79	endoplasmic reticulum protein 29
**LOC474698**	474698	-0,21	-0,27	-0,47	-0,43	similar to catenin (cadherin-associated protein), alpha 1, 102kDa
**SUCLA2**	485448	-0,35	-0,43	-0,47	-0,50	succinate-CoA ligase, ADP-forming, beta subunit
**NDUFS2**	478981	-0,26	-0,14	-0,47	-0,60	NADH dehydrogenase (ubiquinone) Fe-S protein 2, 49kDa (NADH-coenzyme Q reductase)
**RPS19**	476450	0,11	-0,03	-0,47	-0,22	ribosomal protein S19
**GNA11**	403914	-0,36	-0,23	-0,47	-0,19	guanine nucleotide binding protein (G protein), alpha 11 (Gq class)
**CTNNB1**	477032	-0,28	-0,43	-0,47	-0,56	catenin (cadherin-associated protein), beta 1, 88kDa
**HNRNPH1**	481455	-0,32	-0,32	-0,46	-0,55	heterogeneous nuclear ribonucleoprotein H1 (H)
**RPS16**	476463	0,11	0,07	-0,46	-0,18	ribosomal protein S16
**UBXN4**	476130	-0,39	-0,52	-0,45	-0,61	UBX domain protein 4
**LOC491080**	491080	-0,42	-0,41	-0,45	-0,47	similar to CG3408-PA

		log2 fold change				
**Symbol**	**Entrez Id**	**4hrs p.i.**	**8 hrs p.i.**	**10 hrs p.i.**	**12 hrs p.i.**	**Name**
**SRPR**	489293	-0,26	-0,78	-0,45	-0,58	signal recognition particle receptor (docking protein)
**RBM28**	475203	-0,33	0,06	-0,45	-0,51	RNA binding motif protein 28
**CAT**	403474	-0,51	-0,70	-0,45	-0,68	catalase
**ZONAB**	403728	-0,46	-0,55	-0,45	-0,71	Y-box protein ZONAB-A
**HNRNPD**	478450	-0,06	-0,23	-0,45	-0,32	heterogeneous nuclear ribonucleoprotein D (AU-rich element RNA binding protein 1, 37kDa)
**RTN3**	476043	-0,42	-0,47	-0,44	-0,43	reticulon 3
**UQCRC1**	608455	-0,09	-0,36	-0,44	-0,38	ubiquinol-cytochrome c reductase core protein I
**RAB8A**	403773	-0,37	-0,59	-0,44	-0,52	RAB8A, member RAS oncogene family
**PPIB**	478337	-0,25	-0,37	-0,44	-0,35	peptidylprolyl isomerase B (cyclophilin B)
**DRAP1**	476018	-0,05	-0,48	-0,43	-0,34	DR1-associated protein 1 (negative cofactor 2 alpha)
**POGLUT1**	487993	-0,17	-0,57	-0,43	-0,71	protein O-glucosyltransferase 1
**LOC480971**	480971	-0,44	-0,85	-0,43	-0,29	similar to Ewing sarcoma breakpoint region 1 isoform EWS
**RPL10**	481085	0,09	0,09	-0,42	-0,05	ribosomal protein L10
**HK1**	479234	-0,36	-0,19	-0,42	-0,17	hexokinase 1
**PRRC1**	481489	-0,34	-0,78	-0,41	-0,86	proline-rich coiled-coil 1
**DDX18**	476120	-0,20	-0,10	-0,41	-0,38	DEAD (Asp-Glu-Ala-Asp) box polypeptide 18
**NOP10**	607299	-0,37	-0,32	-0,41	-0,58	NOP10 ribonucleoprotein homolog (yeast)
**ACSS1**	477002	-0,34	-0,53	-0,41	-0,22	acyl-CoA synthetase short-chain family member 1
**EIF6**	477210	-0,40	-0,42	-0,41	-0,49	eukaryotic translation initiation factor 6
**NMT1**	480494	-0,03	-0,14	-0,41	-0,29	N-myristoyltransferase 1
**PRDX4**	491776	-0,15	-0,48	-0,41	-0,45	peroxiredoxin 4
**IGF2BP2**	478662	0,01	-0,18	-0,41	-0,64	insulin-like growth factor 2 mRNA binding protein 2
**NLE1**	491142	-0,31	-0,61	-0,40	-0,37	notchless homolog 1 (Drosophila)
**HNRNPA3**	608074	-0,26	-0,23	-0,40	-0,34	heterogeneous nuclear ribonucleoprotein A3
**BAX**	403523	-0,24	-0,68	-0,40	0,02	BCL2-associated X protein
**RPL17**	475077	0,14	0,10	-0,40	-0,11	ribosomal protein L17
**UGGT1**	476101	-0,37	-0,61	-0,39	-0,57	UDP-glucose glycoprotein glucosyltransferase 1
**TM9SF3**	612786	-0,50	-0,31	-0,39	-0,38	transmembrane 9 superfamily member 3
**FAU**	483748	0,06	-0,22	-0,39	-0,17	Finkel-Biskis-Reilly murine sarcoma virus (FBR-MuSV) ubiquitously expressed
**EIF2S1**	480361	0,04	-0,09	-0,39	-0,18	eukaryotic translation initiation factor 2, subunit 1 alpha, 35kDa
**COPB2**	477088	-0,20	-0,42	-0,38	-0,40	coatomer protein complex, subunit beta 2 (beta prime)
**HADHA**	475687	-0,38	-0,48	-0,38	-0,47	hydroxyacyl-CoA dehydrogenase/3-ketoacyl-CoA thiolase/enoyl-CoA hydratase (trifunctional protein), alpha subunit
**LOC608656**	608656	-0,47	-0,48	-0,37	-0,58	similar to oxoglutarate (alpha-ketoglutarate) dehydrogenase (lipoamide) isoform 1 precursor
**MTX1**	480130	-0,19	-0,44	-0,37	-0,31	metaxin 1
**NDUFAB1**	479805	-0,21	-0,48	-0,37	-0,51	NADH dehydrogenase (ubiquinone) 1, alpha/beta subcomplex, 1, 8kDa
**ACAD9**	476519	-0,15	-0,36	-0,36	-0,38	acyl-CoA dehydrogenase

Symbol	Entrez Id	log2 fold change				Name
		4hrs p.i.	8 hrs p.i.	10 hrs p.i.	12 hrs p.i.	
						family, member 9
**DAO**	486317	-0,19	-0,41	-0,36	-0,33	D-amino-acid oxidase
**ACADVL**	489463	-0,37	-0,50	-0,36	-0,47	acyl-CoA dehydrogenase, very long chain
**SRSF1**	609693	-0,27	-0,36	-0,36	-0,53	serine/arginine-rich splicing factor 1
**MANF**	608421	0,00	-0,40	-0,36	-0,34	mesencephalic astrocyte-derived neurotrophic factor
**LOC477839**	477839	-0,29	-0,63	-0,36	-0,47	similar to Thioredoxin-dependent peroxide reductase, mitochondrial precursor (Peroxiredoxin 3) (Antioxidant protein 1) (AOP-1) (MER5 protein homolog) (HBC189) (PRX III)
**EIF4A3**	475922	-0,12	-0,21	-0,36	-0,29	eukaryotic translation initiation factor 4A3
**LOC479283**	479283	0,01	-0,19	-0,36	-0,32	similar to Activated RNA polymerase II transcriptional coactivator p15 (Positive cofactor 4) (PC4) (p14)
**NCAPD2**	477715	-0,06	-0,49	-0,35	-0,45	non-SMC condensin I complex, subunit D2
**RPS28**	485010	0,17	0,08	-0,35	-0,12	ribosomal protein S28
**HIBADH**	479610	-0,28	-0,65	-0,35	-0,31	3-hydroxyisobutyrate dehydrogenase
**RBM47**	403423	-0,50	-0,34	-0,35	-0,58	RNA binding motif protein 47
**RPL24**	478547	0,14	0,10	-0,35	-0,14	ribosomal protein L24
**P4HB**	483369	-0,19	-0,61	-0,34	-0,42	prolyl 4-hydroxylase, beta polypeptide
**LOC477695**	477695	-0,18	-0,40	-0,34	-0,34	similar to mago-nashi homolog
**F3**	490153	-0,18	-0,32	-0,34	-0,50	coagulation factor III (thromboplastin, tissue factor)
**SERPINH1**	485187	-0,24	-0,37	-0,34	-0,43	serpin peptidase inhibitor, clade H (heat shock protein 47), member 1, (collagen binding protein 1)
**HMGB1**	403170	0,13	-0,48	-0,33	-0,52	high mobility group box 1
**LMNB2**	485070	-0,01	-0,26	-0,33	-0,69	lamin B2
**ATP5A1**	480149	-0,27	-0,38	-0,33	-0,34	ATP synthase, H+ transporting, mitochondrial F1 complex, alpha subunit 1, cardiac muscle
**DNAJB11**	478664	-0,19	-0,10	-0,33	-0,25	DnaJ (Hsp40) homolog, subfamily B, member 11
**SF3B1**	488456	-0,29	-0,45	-0,31	-0,43	splicing factor 3b, subunit 1, 155kDa
**SHMT2**	607458	-0,23	-0,33	-0,31	-0,42	serine hydroxymethyltransferase 2 (mitochondrial)
**MAK16**	475593	-0,37	-0,21	-0,31	-0,14	MAK16 homolog (S. cerevisiae)
**GANAB**	483784	-0,19	-0,58	-0,31	-0,48	glucosidase, alpha; neutral AB
**ACOT9**	480864	0,05	-0,28	-0,31	-0,17	acyl-CoA thioesterase 9
**PNN**	403907	-0,01	-0,41	-0,31	-0,37	pinin, desmosome associated protein
**AP1M2**	611211	0,01	-0,37	-0,31	-0,36	adaptor-related protein complex 1, mu 2 subunit
**SRSF2**	612817	-0,21	-0,19	-0,29	-0,38	serine/arginine-rich splicing factor 2
**HYOU1**	479410	-0,24	-0,44	-0,29	-0,32	hypoxia up-regulated 1
**NOP16**	479282	0,11	0,14	-0,29	0,44	NOP16 nucleolar protein homolog (yeast)
**MYO6**	481884	-0,01	-0,40	-0,29	-0,36	myosin VI
**HNRPDL**	476976	0,03	-0,17	-0,28	-0,12	heterogeneous nuclear ribonucleoprotein D-like
**LOC480466**	480466	-0,02	-0,17	-0,28	-0,21	similar to ELAV-like protein 1 (Hu-antigen R) (HuR)

		log2 fold change				
**Symbol**	**Entrez Id**	**4hrs p.i.**	**8 hrs p.i.**	**10 hrs p.i.**	**12 hrs p.i.**	**Name**
**HSPA9**	474697	-0,24	-0,34	-0,28	-0,30	heat shock 70kDa protein 9 (mortalin)
**LOC476328**	476328	0,20	0,00	-0,28	-0,22	similar to 60S ribosomal protein L29 (Cell surface heparin binding protein HIP)
**BLMH**	480635	-0,21	-1,33	-0,27	-0,96	bleomycin hydrolase
**BANF1**	611961	-0,31	-0,45	-0,27	-0,65	barrier to autointegration factor 1
**RPL13**	479612	0,20	0,08	-0,27	0,03	ribosomal protein L13
**SOD2**	476258	-0,09	-0,50	-0,26	-0,30	superoxide dismutase 2, mitochondrial
**IARS2**	478963	-0,42	-0,75	-0,26	-0,52	isoleucyl-tRNA synthetase 2, mitochondrial
**HSPA5**	480726	-0,21	-0,55	-0,26	-0,28	heat shock 70kDa protein 5 (glucose-regulated protein, 78kDa)
**SRSF3**	403687	-0,29	-0,35	-0,26	-0,50	serine/arginine-rich splicing factor 3
**SUCLG2**	476562	-0,02	-0,37	-0,25	-0,25	succinate-CoA ligase, GDP-forming, beta subunit
**AIMP2**	479752	-0,03	-0,41	-0,25	-0,08	aminoacyl tRNA synthetase complex-interacting multifunctional protein 2
**LOC490572**	490572	0,10	-0,09	-0,25	-0,15	similar to myosin, light polypeptide 6, alkali, smooth muscle and non-muscle
**HNRNPL**	476466	-0,09	-0,04	-0,25	-0,19	heterogeneous nuclear ribonucleoprotein L
**EIF2S2**	477197	0,09	-0,17	-0,25	-0,02	eukaryotic translation initiation factor 2, subunit 2 beta, 38kDa
**LOC490810**	490810	-0,27	-0,57	-0,25	-0,60	similar to CG33071-PC, isoform C
**UTRN**	442965	-0,25	-0,72	-0,25	-0,52	utrophin
**PYCR2**	480112	-0,13	-0,70	-0,25	-0,44	pyrroline-5-carboxylate reductase family, member 2
**ILF2**	480139	-0,03	-0,05	-0,24	-0,25	interleukin enhancer binding factor 2, 45kDa
**P4HA2**	474672	0,00	-0,20	-0,23	-0,23	prolyl 4-hydroxylase, alpha polypeptide II
**RPL28**	476378	0,32	0,22	-0,23	0,05	ribosomal protein L28
**SDHA**	478634	-0,18	-0,44	-0,23	-0,37	succinate dehydrogenase complex, subunit A, flavoprotein (Fp)
**NOP56**	612871	-0,16	-0,11	-0,22	-0,27	NOP56 ribonucleoprotein homolog (yeast)
**PNPT1**	481376	-0,21	-0,52	-0,22	-0,89	polyribonucleotide nucleotidyltransferase 1
**NUP155**	479360	-0,12	-0,10	-0,22	-0,31	nucleoporin 155kDa
**PITRM1**	478018	-0,15	-0,10	-0,22	-0,29	pitrilysin metallopeptidase 1
**HSP90B1**	404019	-0,10	-0,52	-0,21	-0,31	heat shock protein 90kDa beta (Grp94), member 1
**OXCT1**	479347	0,17	-0,15	-0,21	-0,17	3-oxoacid CoA transferase 1
**HADH**	478506	-0,20	-0,56	-0,21	-0,35	hydroxyacyl-CoA dehydrogenase
**TAS1R2**	478213	0,07	-0,11	-0,21	0,05	taste receptor, type 1, member 2
**ETFA**	610134	-0,17	-0,24	-0,21	-0,15	electron-transfer-flavoprotein, alpha polypeptide
**ESRP2**	489745	0,19	0,01	-0,20	-0,24	epithelial splicing regulatory protein 2
**LOC488888**	488888	0,25	-0,38	-0,20	-0,06	hypothetical LOC488888
**DLST**	480388	-0,08	-0,17	-0,20	-0,25	dihydrolipoamide S-succinyltransferase (E2 component of 2-oxo-glutarate complex)
**ERO1L**	490691	-0,11	-0,51	-0,20	-0,33	ERO1-like (S. cerevisiae)
**LOC478864**	478864	0,21	0,12	-0,20	0,01	similar to 60S ribosomal protein L21
**RPL19**	403682	0,31	0,23	-0,20	0,09	ribosomal protein L19

		log2 fold change				
**Symbol**	**Entrez Id**	**4hrs p.i.**	**8 hrs p.i.**	**10 hrs p.i.**	**12 hrs p.i.**	**Name**
**STAT3**	490967	0,37	-0,18	-0,19	-0,45	signal transducer and activator of transcription 3 (acute-phase response factor)
**CALR**	476694	-0,09	-0,58	-0,19	-0,29	calreticulin
**CNPY2**	607115	-0,21	-0,58	-0,19	-0,26	canopy 2 homolog (zebrafish)
**RAB11B**	611619	-0,06	-0,15	-0,19	-0,20	RAB11B, member RAS oncogene family
**ASNA1**	476699	-0,24	-0,86	-0,19	-0,57	arsA arsenite transporter, ATP-binding, homolog 1 (bacterial)
**PDIA3**	478279	-0,06	-0,36	-0,19	-0,12	protein disulfide isomerase family A, member 3
**PRKCSH**	484941	-0,23	-0,53	-0,19	-0,52	protein kinase C substrate 80K-H
**ACAA1**	477023	-0,25	-0,72	-0,19	-0,29	acetyl-CoA acyltransferase 1
**SNRPF**	482609	0,01	-0,33	-0,18	-0,38	small nuclear ribonucleoprotein polypeptide F
**SCP2**	479564	0,02	-0,40	-0,18	-0,45	sterol carrier protein 2
**IDH2**	479043	0,08	-0,22	-0,18	-0,01	isocitrate dehydrogenase 2 (NADP+), mitochondrial
**MTHFD1L**	476245	-0,16	-0,47	-0,18	-0,36	methylenetetrahydrofolate dehydrogenase (NADP+ dependent) 1-like
**CAPRIN1**	607420	0,17	0,11	-0,18	-0,05	cell cycle associated protein 1
**TBL3**	490064	0,03	0,04	-0,17	-0,10	transducin (beta)-like 3
**CDC42**	403934	-0,40	-0,64	-0,17	-0,85	cell division cycle 42 (GTP binding protein, 25kDa)
**TUBA1C**	608138	0,23	0,07	-0,17	-0,24	tubulin, alpha 1c
**PCK2**	480255	0,02	-0,18	-0,16	-0,28	phosphoenolpyruvate carboxykinase 2 (mitochondrial)
**PDHB**	476574	-0,25	-0,41	-0,16	-0,32	pyruvate dehydrogenase (lipoamide) beta
**BCAS2**	475805	0,11	0,15	-0,16	-0,07	breast carcinoma amplified sequence 2
**LOC474524**	474524	0,09	0,02	-0,16	-0,11	similar to CG9987-PA
**ME2**	476198	-0,17	-0,57	-0,15	-0,44	malic enzyme 2, NAD(+)-dependent, mitochondrial
**HNRNPA0**	481524	0,11	-0,16	-0,15	-0,04	heterogeneous nuclear ribonucleoprotein A0
**LOC475044**	475044	0,03	-0,07	-0,15	-0,01	similar to Heterogeneous nuclear ribonucleoprotein A1 (Helix-destabilizing protein) (Single-strand binding protein) (hnRNP core protein A1) (HDP)
**STXBP2**	403880	-0,35	-0,61	-0,14	-0,65	syntaxin binding protein 2
**ALDH18A1**	477781	-0,10	-0,29	-0,14	-0,28	aldehyde dehydrogenase 18 family, member A1
**PA2G4**	474396	0,25	0,08	-0,14	0,05	proliferation-associated 2G4, 38kDa
**PTPN11**	477488	-0,03	-1,09	-0,14	-0,24	protein tyrosine phosphatase, non-receptor type 11
**PRPF8**	480651	-0,15	-0,21	-0,14	-0,34	PRP8 pre-mRNA processing factor 8 homolog (S. cerevisiae)
**TUBB6**	480213	0,25	0,07	-0,13	-0,08	tubulin, beta 6
**GNB2**	479733	0,41	0,33	-0,13	0,24	guanine nucleotide binding protein (G protein), beta polypeptide 2
**TIMM44**	485005	-0,18	-0,51	-0,13	-0,50	translocase of inner mitochondrial membrane 44 homolog (yeast)
**LOC607081**	607081	-0,03	0,00	-0,12	-0,16	similar to non-POU domain containing, octamer-binding
**ATP6V0D1**	479685	-0,33	-0,35	-0,12	-0,05	ATPase, H+ transporting, lysosomal 38kDa, V0 subunit

		log2 fold change				
Symbol	Entrez Id	4hrs p.i.	8 hrs p.i.	10 hrs p.i.	12 hrs p.i.	Name
						d1
**LOC478855**	478855	-0,03	-0,29	-0,12	-0,19	similar to heat shock 10kDa protein 1 (chaperonin 10)
**M6PR**	477700	-0,10	-0,16	-0,12	-0,11	mannose-6-phosphate receptor (cation dependent)
**ZNF326**	612406	-0,15	-0,10	-0,12	-0,13	zinc finger protein 326
**ANXA13**	403935	-0,08	-0,12	-0,11	-0,04	annexin A13
**AP1G1**	479666	0,20	-0,07	-0,11	-0,14	adaptor-related protein complex 1, gamma 1 subunit
**HSPD1**	478854	-0,11	-0,29	-0,11	-0,14	heat shock 60kDa protein 1 (chaperonin)
**EIF1**	403674	0,17	-0,24	-0,11	-0,14	eukaryotic translation initiation factor 1
**PSPC1**	477347	-0,07	-0,09	-0,11	-0,27	paraspeckle component 1
**RNPS1**	479880	-0,13	-0,32	-0,11	-0,23	RNA binding protein S1, serine-rich domain
**HNRNPCL1**	474964	0,14	-0,05	-0,10	0,00	heterogeneous nuclear ribonucleoprotein C-like 1
**TUBB**	474830	0,19	0,03	-0,10	-0,24	tubulin, beta
**PRKCDBP**	476831	0,35	-0,24	-0,10	-0,10	protein kinase C, delta binding protein
**NOP58**	607667	0,01	0,02	-0,10	-0,11	NOP58 ribonucleoprotein homolog (yeast)
**TIAL1**	477841	-0,04	-0,12	-0,10	-0,32	TIA1 cytotoxic granule-associated RNA binding protein-like 1
**RDX**	479446	0,03	-0,30	-0,10	-0,06	radixin
**RPL23**	403683	0,26	0,12	-0,09	0,06	ribosomal protein L23
**PDIA6**	475668	0,05	-0,37	-0,09	-0,14	protein disulfide isomerase family A, member 6
**GLS**	488448	0,00	-0,04	-0,08	0,08	glutaminase
**SNRNP70**	484388	-0,08	-0,28	-0,08	-0,27	small nuclear ribonucleoprotein 70kDa (U1)
**HSD17B10**	480930	-0,24	-0,37	-0,08	-0,21	hydroxysteroid (17-beta) dehydrogenase 10
**DHX15**	488856	0,25	0,36	-0,07	0,14	DEAH (Asp-Glu-Ala-His) box polypeptide 15
**AKR7A2**	478210	0,32	-0,13	-0,07	-0,41	aldo-keto reductase family 7, member A2 (aflatoxin aldehyde reductase)
**MRPS31**	477294	-0,04	-0,30	-0,07	-0,31	mitochondrial ribosomal protein S31
**TOM1**	608746	-0,38	-0,73	-0,07	-0,36	target of myb1 (chicken)
**SNRPD1**	480178	-0,29	-0,41	-0,07	-0,35	small nuclear ribonucleoprotein D1 polypeptide 16kDa
**SNRNP200**	475742	0,09	-0,05	-0,07	-0,14	small nuclear ribonucleoprotein 200kDa (U5)
**GPNMB**	482355	0,02	-0,18	-0,07	-0,01	glycoprotein (transmembrane) nmb
**LOC474696**	474696	0,31	0,07	-0,06	0,03	similar to eukaryotic translation termination factor 1 (predicted)
**CS**	474403	0,05	-0,25	-0,06	-0,17	citrate synthase
**ABCF1**	474826	0,28	0,02	-0,06	-0,19	ATP-binding cassette, sub-family F (GCN20), member 1
**STX7**	483987	-0,25	0,10	-0,06	-0,06	syntaxin 7
**LOC610135**	610135	0,09	-0,17	-0,06	-0,12	similar to small nuclear ribonucleoprotein polypeptide G
**LONP1**	611425	0,16	-0,08	-0,06	-0,07	lon peptidase 1, mitochondrial
**JUP**	480522	0,22	-0,01	-0,05	-0,03	junction plakoglobin
**TUBB2A**	478701	0,10	0,03	-0,05	-0,19	tubulin, beta 2A
**CALU**	475201	-0,03	-0,41	-0,05	-0,08	calumenin
**SUCLG1**	475775	0,15	-0,10	-0,05	-0,09	succinate-CoA ligase, alpha subunit
**LOC477554**	477554	0,03	-0,20	-0,05	-0,11	similar to small nuclear

		log2 fold change				
**Symbol**	**Entrez Id**	**4hrs p.i.**	**8 hrs p.i.**	**10 hrs p.i.**	**12 hrs p.i.**	**Name**
						ribonucleoprotein D3
**PFDN5**	477597	-0,08	-0,85	-0,05	-0,27	prefoldin subunit 5
**MYOF**	607472	-0,46	-0,20	-0,04	-0,53	myoferlin
**FECH**	610360	-0,19	-0,66	-0,04	-0,15	ferrochelatase
**FH**	480092	-0,01	-0,40	-0,03	-0,22	fumarate hydratase
**CAPZA2**	503863	0,09	-0,47	-0,03	-0,10	capping protein (actin filament) muscle Z-line, alpha 2
**PDHA1**	480858	0,12	-0,15	-0,03	0,06	pyruvate dehydrogenase (lipoamide) alpha 1
**RAB5C**	403941	0,02	-0,32	-0,03	-0,03	RAB5C, member RAS oncogene family
**ACAT1**	489421	0,07	-0,14	-0,03	0,00	acetyl-CoA acetyltransferase 1
**DLD**	403978	0,10	-0,19	-0,03	-0,12	dihydrolipoamide dehydrogenase
**MYH9**	481280	0,46	0,26	-0,03	0,10	myosin, heavy chain 9, non-muscle
**SF3B3**	479659	0,02	-0,16	-0,02	-0,17	splicing factor 3b, subunit 3, 130kDa
**EIF5A**	489468	0,45	0,19	-0,02	0,01	eukaryotic translation initiation factor 5A
**DSG2**	490500	-0,28	-0,61	-0,02	-0,07	desmoglein 2
**MYL12B**	480193	0,43	0,31	-0,02	0,19	myosin, light chain 12B, regulatory
**RPL23A**	476799	0,52	0,35	-0,01	0,17	ribosomal protein L23a
**NAPA**	612960	-0,03	-0,29	-0,01	-0,20	N-ethylmaleimide-sensitive factor attachment protein, alpha
**ACO1**	481576	0,09	-0,53	-0,01	-0,27	aconitase 1, soluble
**AP2B1**	480605	0,15	-0,24	-0,01	-0,11	adaptor-related protein complex 2, beta 1 subunit
**POLR2E**	611859	0,02	-0,43	0,00	-0,17	polymerase (RNA) II (DNA directed) polypeptide E, 25kDa
**C1QBP**	489450	0,12	0,14	0,00	0,18	complement component 1, q subcomponent binding protein
**LMNB1**	474663	0,27	0,19	0,00	-0,03	lamin B1
**SRSF5**	490754	0,16	0,07	0,00	0,08	serine/arginine-rich splicing factor 5
**ALDH5A1**	488246	0,16	-0,25	0,01	-0,34	aldehyde dehydrogenase 5 family, member A1
**EPS8**	477686	0,06	-0,47	0,01	-0,20	epidermal growth factor receptor pathway substrate 8
**TUFM**	479796	-0,11	-0,33	0,01	-0,19	Tu translation elongation factor, mitochondrial
**SRSF7**	475730	-0,05	-0,26	0,01	-0,22	serine/arginine-rich splicing factor 7
**NUCB1**	484391	-0,16	-0,13	0,01	-0,17	nucleobindin 1
**MATR3**	474700	0,03	-0,05	0,02	-0,08	matrin 3
**MCM4**	477871	0,52	-0,03	0,02	0,40	minichromosome maintenance complex component 4
**MKI67IP**	476111	0,06	0,05	0,03	0,01	MKI67 (FHA domain) interacting nucleolar phosphoprotein
**NOL6**	481582	-0,30	-0,25	0,03	-0,25	nucleolar protein family 6 (RNA-associated)
**NOB1**	489733	0,28	0,33	0,03	0,25	NIN1/RPN12 binding protein 1 homolog (S. cerevisiae)
**EFTUD2**	490935	0,00	-0,11	0,03	-0,29	elongation factor Tu GTP binding domain containing 2
**TUBB2C**	491231	0,25	0,14	0,04	-0,11	tubulin, beta 2C
**CCAR1**	479230	0,19	-0,04	0,04	-0,06	cell division cycle and apoptosis regulator 1
**RBM3**	609457	0,04	-0,01	0,04	-0,01	RNA binding motif (RNP1, RRM) protein 3
**SNRPE**	478934	0,06	-0,25	0,04	-0,15	small nuclear ribonucleoprotein

		log2 fold change				
Symbol	Entrez Id	4hrs p.i.	8 hrs p.i.	10 hrs p.i.	12 hrs p.i.	Name
						polypeptide E
**PMPCA**	480677	-0,11	-0,37	0,04	-0,19	peptidase (mitochondrial processing) alpha
**NXF1**	483780	0,09	-0,22	0,04	-0,15	nuclear RNA export factor 1
**UBAP2L**	480135	0,20	-0,28	0,04	-0,02	ubiquitin associated protein 2-like
**ACO2**	474487	0,07	-0,20	0,04	-0,05	aconitase 2, mitochondrial
**TRIM28**	403689	0,29	-0,21	0,05	-0,13	tripartite motif containing 28
**LOC479969**	479969	0,30	0,25	0,05	0,19	similar to Protein C14orf166
**SAR1A**	479237	0,31	0,05	0,05	0,11	SAR1 homolog A (S. cerevisiae)
**VAPB**	477274	0,16	0,01	0,05	0,00	VAMP (vesicle-associated membrane protein)-associated protein B and C
**CPSF7**	476066	0,01	-0,17	0,06	-0,05	cleavage and polyadenylation specific factor 7, 59kDa
**CHD4**	477714	0,29	0,11	0,06	0,20	chromodomain helicase DNA binding protein 4
**ALB**	403550	-8,44	-7,87	0,06	-7,76	albumin
**TMPO**	482618	-0,11	-0,10	0,06	-0,19	thymopoietin
**PRKAG1**	486559	0,34	-0,28	0,06	0,07	protein kinase, AMP-activated, gamma 1 non-catalytic subunit
**TOP2A**	480525	0,19	-0,37	0,07	-0,04	topoisomerase (DNA) II alpha 170kDa
**SND1**	475204	0,42	0,18	0,07	0,04	staphylococcal nuclease and tudor domain containing 1
**PTRF**	490966	0,41	0,01	0,07	0,06	polymerase I and transcript release factor
**LOC474576**	474576	0,12	0,12	0,07	-0,04	similar to DnaJ homolog subfamily A member 1 (Heat shock 40 kDa protein 4) (DnaJ protein homolog 2) (HSJ-2) (HSDJ)
**RPS14**	403690	0,41	0,31	0,08	0,28	ribosomal protein S14
**GOT2**	478103	0,08	-0,16	0,08	-0,01	glutamic-oxaloacetic transaminase 2, mitochondrial (aspartate aminotransferase 2)
**ASAH1**	482897	0,03	-0,54	0,08	0,04	N-acylsphingosine amidohydrolase (acid ceramidase) 1
**LOC482601**	482601	-0,02	-0,25	0,08	0,00	similar to malate dehydrogenase 2, NAD (mitochondrial)
**MSN**	491924	0,29	-0,34	0,09	-0,15	moesin
**TRAP1**	490032	0,04	-0,29	0,09	-0,07	TNF receptor-associated protein 1
**SMU1**	474740	0,35	0,18	0,09	-0,13	smu-1 suppressor of mec-8 and unc-52 homolog (C. elegans)
**CAMK2D**	610764	0,51	-0,10	0,09	-0,02	calcium/calmodulin-dependent protein kinase II delta
**METAP2**	475429	0,33	0,05	0,09	0,09	methionyl aminopeptidase 2
**NAA15**	483817	0,20	-0,19	0,09	0,04	N(alpha)-acetyltransferase 15, NatA auxiliary subunit
**OGDH**	475499	0,05	-0,33	0,09	-0,04	oxoglutarate (alpha-ketoglutarate) dehydrogenase (lipoamide)
**LOC609251**	609251	0,24	-0,36	0,09	-0,05	similar to niban protein
**SNX4**	478595	0,22	-0,60	0,10	-0,12	sorting nexin 4
**GRSF1**	475170	0,13	-0,05	0,10	-0,01	G-rich RNA sequence binding factor 1
**OAT**	477858	-0,11	-0,14	0,10	0,09	ornithine aminotransferase
**ARF6**	490679	0,21	-0,43	0,10	-0,29	ADP-ribosylation factor 6
**GRPEL1**	488784	0,20	-0,15	0,10	0,03	GrpE-like 1, mitochondrial (E. coli)
**YARS**	478148	0,18	-0,28	0,11	0,22	tyrosyl-tRNA synthetase

		log2 fold change				
**Symbol**	**Entrez Id**	**4hrs p.i.**	**8 hrs p.i.**	**10 hrs p.i.**	**12 hrs p.i.**	**Name**
**COL17A1**	403739	0,39	-0,39	0,11	-0,38	collagen, type XVII, alpha 1
**NARS**	607631	0,41	0,02	0,11	0,32	asparaginyl-tRNA synthetase
**MNDA**	488622	-0,07	-0,49	0,11	0,34	myeloid cell nuclear differentiation antigen
**SRP68**	403952	0,30	0,26	0,11	0,01	signal recognition particle 68kDa
**LOC479071**	479071	0,35	0,33	0,11	0,20	similar to Probable ATP-dependent RNA helicase DDX6 (DEAD-box protein 6) (ATP-dependent RNA helicase p54) (Oncogene RCK)
**PRPF40A**	476152	0,07	-0,12	0,11	-0,16	PRP40 pre-mRNA processing factor 40 homolog A (S. cerevisiae)
**HNRNPF**	477757	0,14	0,11	0,11	0,03	heterogeneous nuclear ribonucleoprotein F
**SEC22B**	475816	0,24	0,08	0,11	0,09	SEC22 vesicle trafficking protein homolog B (S. cerevisiae) (gene/pseudogene)
**IQGAP1**	479050	0,14	-0,19	0,12	0,29	IQ motif containing GTPase activating protein 1
**HNRNPH3**	479227	0,09	0,00	0,12	0,02	heterogeneous nuclear ribonucleoprotein H3 (2H9)
**HIBCH**	607040	-0,11	-0,42	0,12	-0,21	3-hydroxyisobutyryl-CoA hydrolase
**LOC478254**	478254	0,24	0,20	0,12	0,25	similar to CG5913-PA
**IDH3B**	477177	0,06	-0,05	0,12	0,23	isocitrate dehydrogenase 3 (NAD+) beta
**LOC475374**	475374	0,22	-1,01	0,13	-0,48	similar to Alcohol dehydrogenase [NADP+] (Aldehyde reductase) (Aldo-keto reductase family 1 member A1)
**RPS20**	477887	0,45	0,33	0,13	0,30	ribosomal protein S20
**PSIP1**	474712	0,15	-0,35	0,13	-0,05	PC4 and SFRS1 interacting protein 1
**VAT1**	490953	0,27	-0,33	0,13	-0,04	vesicle amine transport protein 1 homolog (T. californica)
**PRMT5**	480242	0,31	-0,18	0,13	0,32	protein arginine methyltransferase 5
**LOC607058**	607058	0,00	-0,30	0,14	-0,05	similar to SVH protein
**LOC477550**	477550	0,28	-0,22	0,14	0,00	similar to poly(rC) binding protein 2 (predicted)
**ATPIF1**	478165	0,36	0,20	0,14	0,34	ATPase inhibitory factor 1
**RPS7**	475649	0,66	0,53	0,14	0,39	ribosomal protein S7
**ALDH6A1**	490779	0,14	-0,26	0,14	0,07	aldehyde dehydrogenase 6 family, member A1
**DDX17**	474508	0,36	0,17	0,14	0,13	DEAD (Asp-Glu-Ala-Asp) box polypeptide 17
**LOC476337**	476337	0,19	-0,66	0,15	0,05	similar to D19Bwg1357e protein
**RPL36AL**	480311	0,70	0,42	0,15	0,45	ribosomal protein L36a-like
**GOLPH3L**	475838	0,32	0,00	0,15	-0,10	golgi phosphoprotein 3-like
**POLR1C**	474912	0,17	-0,08	0,15	0,16	polymerase (RNA) I polypeptide C, 30kDa
**HNRNPUL2**	476053	0,33	0,07	0,15	0,09	heterogeneous nuclear ribonucleoprotein U-like 2
**HNRNPA2B1**	475260	0,20	0,10	0,15	0,21	heterogeneous nuclear ribonucleoprotein A2/B1
**FARSA**	476695	0,38	-0,09	0,15	0,07	phenylalanyl-tRNA synthetase, alpha subunit
**ECHS1**	480828	0,06	-0,25	0,16	-0,10	enoyl CoA hydratase, short chain, 1, mitochondrial
**LMNA**	480124	0,42	0,36	0,16	0,21	lamin A/C
**EIF1AX**	611715	0,73	0,39	0,17	0,22	eukaryotic translation initiation factor 1A, X-linked
**BUB3**	477857	0,51	0,13	0,17	0,30	budding uninhibited by

		log2 fold change				
Symbol	Entrez Id	4hrs p.i.	8 hrs p.i.	10 hrs p.i.	12 hrs p.i.	Name
						benzimidazoles 3 homolog (yeast)
**COPE**	476663	0,29	0,12	0,17	0,07	coatomer protein complex, subunit epsilon
**RPS27**	490449	0,47	0,34	0,17	0,23	ribosomal protein S27
**DDX1**	475671	0,33	0,43	0,18	0,22	DEAD (Asp-Glu-Ala-Asp) box polypeptide 1
**SEC13**	607653	0,90	0,41	0,18	0,69	SEC13 homolog (S. cerevisiae)
**RPS6**	474722	0,65	0,59	0,19	0,47	ribosomal protein S6
**CHADL**	481234	0,68	-0,17	0,20	-0,50	chondroadherin-like
**SNRPB**	485806	0,51	0,18	0,20	0,24	small nuclear ribonucleoprotein polypeptides B and B1
**VAMP8**	609784	0,36	0,10	0,20	0,18	vesicle-associated membrane protein 8 (endobrevin)
**RAE1**	485942	0,11	-0,03	0,21	0,05	RAE1 RNA export 1 homolog (S. pombe)
**WDR61**	479068	0,21	-0,08	0,21	0,47	WD repeat domain 61
**CARS**	475998	0,32	-0,33	0,21	0,05	cysteinyl-tRNA synthetase
**IFIT5**	486788	0,67	0,02	0,21	0,62	interferon-induced protein with tetratricopeptide repeats 5
**GRWD1**	612058	0,35	0,21	0,21	0,16	glutamate-rich WD repeat containing 1
**ABCE1**	475454	0,37	-0,01	0,22	0,13	ATP-binding cassette, sub-family E (OABP), member 1
**CDK1**	488997	0,27	-0,17	0,22	-0,20	cyclin-dependent kinase 1
**LOC475682**	475682	0,46	0,49	0,22	0,40	similar to splicing factor 3B, 14 kDa subunit
**NACA**	474408	0,62	0,13	0,22	0,12	nascent polypeptide-associated complex alpha subunit
**IVD**	478259	-0,15	-0,34	0,22	-0,13	isovaleryl-CoA dehydrogenase
**MCM5**	610519	0,47	0,15	0,22	0,10	minichromosome maintenance complex component 5
**ALDH7A1**	481486	0,26	-0,44	0,23	-0,17	aldehyde dehydrogenase 7 family, member A1
**RAB5B**	474394	0,30	-0,05	0,23	0,17	RAB5B, member RAS oncogene family
**PRPF19**	611552	0,22	0,08	0,23	0,11	PRP19/PSO4 pre-mRNA processing factor 19 homolog (S. cerevisiae)
**KTN1**	480332	0,32	0,17	0,23	0,25	kinectin 1 (kinesin receptor)
**PSAP**	479240	0,38	-0,21	0,24	0,23	prosaposin
**PPL**	490021	0,38	-0,10	0,24	-0,01	periplakin
**PRKCI**	478686	0,25	-0,37	0,24	-0,03	protein kinase C, iota
**CDK11A**	489591	0,57	0,08	0,24	0,08	cyclin-dependent kinase 11A
**TARS**	479370	0,48	-0,35	0,24	0,16	threonyl-tRNA synthetase
**LOC487948**	487948	0,41	-0,06	0,24	0,22	similar to coproporphyrinogen oxidase
**IDH3A**	479066	0,19	-0,06	0,25	0,16	isocitrate dehydrogenase 3 (NAD+) alpha
**RHOA**	403954	0,29	-0,26	0,25	-0,03	ras homolog gene family, member A
**TUBA3C**	610636	0,51	0,32	0,25	0,14	tubulin, alpha 3c
**USP39**	475767	0,12	0,14	0,25	0,18	ubiquitin specific peptidase 39
**PSMA8**	490508	0,50	-0,23	0,26	0,35	proteasome (prosome, macropain) subunit, alpha type, 8
**KIF5B**	477968	0,44	0,29	0,26	0,32	kinesin family member 5B
**SRRT**	479731	0,25	-0,14	0,26	0,20	serrate RNA effector molecule homolog (Arabidopsis)
**AARS**	479656	0,38	-0,56	0,26	0,03	alanyl-tRNA synthetase

		log2 fold change				
**Symbol**	**Entrez Id**	**4hrs p.i.**	**8 hrs p.i.**	**10 hrs p.i.**	**12 hrs p.i.**	**Name**
**FBL**	476460	0,46	0,46	0,26	0,34	fibrillarin
**SF3B4**	483177	0,18	0,00	0,26	-0,06	splicing factor 3b, subunit 4, 49kDa
**FKBP3**	480306	0,44	0,13	0,26	0,10	FK506 binding protein 3, 25kDa
**CLTA**	474765	0,43	0,05	0,26	-0,06	clathrin, light chain A
**DDX5**	480472	0,38	0,21	0,26	0,20	DEAD (Asp-Glu-Ala-Asp) box polypeptide 5
**TROVE2**	478957	0,47	0,02	0,27	0,25	TROVE domain family, member 2
**EIF5B**	474556	0,48	0,32	0,27	0,33	eukaryotic translation initiation factor 5B
**DSP**	488207	0,49	0,23	0,28	0,18	desmoplakin
**LOC474513**	474513	0,42	-0,04	0,29	0,35	similar to eukaryotic translation initiation factor 3 subunit 6 interacting protein
**PIP5K1A**	475846	0,24	-0,28	0,29	0,16	phosphatidylinositol-4-phosphate 5-kinase, type I, alpha
**NSUN2**	478625	0,19	-0,13	0,29	0,02	NOP2/Sun domain family, member 2
**ACADM**	490207	0,16	-0,07	0,30	-0,28	acyl-CoA dehydrogenase, C-4 to C-12 straight chain
**LOC610939**	610939	0,03	-0,61	0,30	-0,09	similar to LSM5 homolog, U6 small nuclear RNA associated
**RARS**	609803	0,33	-0,38	0,30	0,47	arginyl-tRNA synthetase
**NUDT21**	478123	0,28	-0,19	0,30	0,16	nudix (nucleoside diphosphate linked moiety X)-type motif 21
**TJP2**	403854	0,00	-1,01	0,31	-0,17	tight junction protein 2 (zona occludens 2)
**WARS**	480435	0,37	-0,44	0,31	0,15	tryptophanyl-tRNA synthetase
**LOC488269**	488269	-0,06	-0,42	0,31	-0,52	similar to Histone H1.4 (Histone H1b)
**RRBP1**	403809	0,37	0,05	0,31	0,30	ribosome binding protein 1 homolog 180kDa (dog)
**STRAP**	486663	0,54	0,31	0,31	0,37	serine/threonine kinase receptor associated protein
**LYPLA2**	487374	0,42	-0,30	0,31	-0,24	lysophospholipase II
**LGALS3BP**	483345	0,70	-0,24	0,31	0,20	lectin, galactoside-binding, soluble, 3 binding protein
**EIF3H**	475081	0,31	-0,19	0,31	0,33	eukaryotic translation initiation factor 3, subunit H
**HDLBP**	477431	0,63	0,25	0,31	0,30	high density lipoprotein binding protein
**POLR2H**	478656	0,35	-0,22	0,32	-0,16	polymerase (RNA) II (DNA directed) polypeptide H
**CTSD**	483662	0,22	0,00	0,32	0,25	cathepsin D
**LOC474484**	474484	0,42	-0,04	0,32	0,12	similar to NHP2-like protein 1 (High mobility group-like nuclear protein 2 homolog 1) ([U4/U6.U5] tri-snRNP 15.5 kDa protein) (Sperm specific antigen 1) (Fertilization antigen 1) (FA-1)
**PSMB6**	607466	-0,15	-0,65	0,32	0,00	proteasome (prosome, macropain) subunit, beta type, 6
**GNB2L1**	480818	0,85	0,55	0,33	0,35	guanine nucleotide binding protein (G protein), beta polypeptide 2-like 1
**LOC480437**	480437	0,40	0,08	0,33	0,31	similar to dynein, cytoplasmic, heavy polypeptide 1
**SNRPA1**	607102	0,42	0,21	0,33	0,45	small nuclear ribonucleoprotein polypeptide A'
**ACLY**	607852	0,70	0,28	0,35	0,15	ATP citrate lyase
**LOC489167**	489167	0,55	0,16	0,35	0,44	similar to CG9590-PA

		log2 fold change				
**Symbol**	**Entrez Id**	**4hrs p.i.**	**8 hrs p.i.**	**10 hrs p.i.**	**12 hrs p.i.**	**Name**
**MCM3**	481839	0,58	0,16	0,35	0,31	minichromosome maintenance complex component 3
**LOC607948**	607948	0,55	0,44	0,36	0,46	similar to THO complex subunit 4 (Tho4) (Ally of AML-1 and LEF-1) (Transcriptional coactivator Aly/REF) (bZIP enhancing factor BEF)
**CTNNBL1**	477224	0,66	0,19	0,36	0,37	catenin, beta like 1
**TIMM10**	475968	0,69	0,17	0,36	0,66	translocase of inner mitochondrial membrane 10 homolog (yeast)
**SEPT2**	487886	0,56	-0,02	0,36	0,39	septin 2
**HDGF**	612089	0,30	-0,36	0,36	0,19	hepatoma-derived growth factor
**SCPEP1**	480566	0,27	-0,41	0,36	0,08	serine carboxypeptidase 1
**KPNA4**	478680	0,50	0,13	0,37	0,33	karyopherin alpha 4 (importin alpha 3)
**ACTR1A**	403791	0,56	0,04	0,37	0,33	ARP1 actin-related protein 1 homolog A, centractin alpha (yeast)
**DPY30**	475715	0,20	0,08	0,37	0,31	dpy-30 homolog (C. elegans)
**S100A10**	475851	0,60	0,24	0,37	0,16	S100 calcium binding protein A10
**ETFB**	476400	0,55	0,13	0,37	0,32	electron-transfer-flavoprotein, beta polypeptide
**KARS**	479644	0,43	0,10	0,38	0,57	lysyl-tRNA synthetase
**SSB**	478787	0,47	0,07	0,38	0,44	Sjogren syndrome antigen B (autoantigen La)
**ARG2**	480364	0,15	-0,33	0,38	0,22	arginase, type II
**HK2**	475781	0,38	-0,35	0,38	0,32	hexokinase 2
**TMOD3**	487554	0,55	0,26	0,38	0,39	tropomodulin 3 (ubiquitous)
**RBMX**	481063	0,38	0,15	0,38	0,29	RNA binding motif protein, X-linked
**RCC2**	487412	0,39	0,00	0,38	0,03	regulator of chromosome condensation 2
**OXSR1**	607809	0,47	-0,24	0,38	0,12	oxidative-stress responsive 1
**MSH2**	494002	0,21	-0,13	0,38	0,11	mutS homolog 2, colon cancer, nonpolyposis type 1 (E. coli)
**LOC607254**	607254	0,53	-0,16	0,38	0,15	similar to EF hand domain containing 2
**PAK2**	612814	0,25	-0,73	0,39	-0,30	p21 protein (Cdc42/Rac)-activated kinase 2
**RPSA**	477029	0,79	0,55	0,39	0,57	ribosomal protein SA
**TK1**	483343	0,60	-0,56	0,39	0,23	thymidine kinase 1, soluble
**LOC476840**	476840	0,51	0,02	0,40	0,39	similar to eukaryotic translation initiation factor 3, subunit 5 epsilon, 47kDa
**DDX39B**	474839	0,49	0,09	0,40	0,29	DEAD (Asp-Glu-Ala-Asp) box polypeptide 39B
**SLC9A3R1**	483299	0,42	-0,01	0,40	0,23	solute carrier family 9 (sodium/hydrogen exchanger), member 3 regulator 1
**VPS4B**	607306	0,50	-0,35	0,40	0,37	vacuolar protein sorting 4 homolog B (S. cerevisiae)
**DDX19A**	479658	0,48	-0,04	0,40	0,15	DEAD (Asp-Glu-Ala-As) box polypeptide 19A
**TNPO1**	478093	0,65	0,08	0,40	0,20	transportin 1
**SAFB**	485028	0,73	0,41	0,41	0,69	scaffold attachment factor B
**CIRH1A**	489738	0,51	0,34	0,41	0,35	cirrhosis, autosomal recessive 1A (cirhin)
**MCM2**	484622	0,69	0,22	0,41	0,40	minichromosome maintenance complex component 2
**FARSB**	478926	0,70	0,15	0,42	0,50	phenylalanyl-tRNA synthetase, beta subunit

		log2 fold change				
**Symbol**	**Entrez Id**	**4hrs p.i.**	**8 hrs p.i.**	**10 hrs p.i.**	**12 hrs p.i.**	**Name**
**RPS12**	476206	0,63	0,39	0,42	0,52	ribosomal protein S12
**DNPEP**	478922	0,49	-0,08	0,42	0,38	aspartyl aminopeptidase
**RRM2**	482963	0,67	0,20	0,42	0,17	ribonucleotide reductase M2
**XPO1**	474609	0,43	-0,16	0,42	0,19	exportin 1 (CRM1 homolog, yeast)
**CCT6B**	491146	0,57	0,20	0,42	0,43	chaperonin containing TCP1, subunit 6B (zeta 2)
**HNRNPM**	476725	0,55	0,31	0,42	0,57	heterogeneous nuclear ribonucleoprotein M
**OSBP**	483448	0,35	-0,07	0,42	0,18	oxysterol binding protein
**MCM6**	476131	0,56	-0,09	0,42	0,35	minichromosome maintenance complex component 6
**TES**	475293	0,67	-0,39	0,42	0,16	testis derived transcript (3 LIM domains)
**PLCD1**	485586	0,64	0,39	0,43	0,26	phospholipase C, delta 1
**RBM39**	477216	0,54	0,17	0,43	0,37	RNA binding motif protein 39
**MAP4**	484781	0,38	-0,42	0,43	0,22	microtubule-associated protein 4
**ACTC1**	478250	0,43	-0,18	0,43	0,28	actin, alpha, cardiac muscle 1
**RPL5**	479949	0,82	0,45	0,43	0,59	ribosomal protein L5
**ARPC1B**	479744	0,57	-0,19	0,44	0,18	actin related protein 2/3 complex, subunit 1B, 41kDa
**EIF3J**	487532	0,38	-0,16	0,44	0,74	eukaryotic translation initiation factor 3, subunit J
**GTF2I**	479714	-0,70	-1,10	0,44	-0,40	general transcription factor IIi
**PPP2R2A**	477374	0,62	-0,02	0,44	0,25	protein phosphatase 2, regulatory subunit B, alpha
**LOC475156**	475156	0,20	-0,34	0,44	0,23	similar to peroxiredoxin 1
**SCFD1**	480281	0,36	-0,13	0,45	0,37	sec1 family domain containing 1
**LTA4H**	482611	0,46	-0,32	0,45	-0,02	leukotriene A4 hydrolase
**PAFAH1B3**	476449	0,41	-0,55	0,45	-0,01	platelet-activating factor acetylhydrolase 1b, catalytic subunit 3 (29kDa)
**USO1**	478430	0,48	0,06	0,45	0,34	USO1 vesicle docking protein homolog (yeast)
**LOC479165**	479165	0,21	-0,80	0,45	0,01	similar to Dihydrofolate reductase
**BCLAF1**	483999	0,46	0,17	0,46	0,39	BCL2-associated transcription factor 1
**GLO1**	474894	0,67	-0,60	0,46	0,08	glyoxalase I
**SEPT7**	475284	0,44	-0,07	0,46	0,21	septin 7
**LOC475721**	475721	0,72	0,67	0,46	1,37	similar to CG5913-PA
**ACP6**	475822	0,03	-0,16	0,46	0,10	acid phosphatase 6, lysophosphatidic
**GORASP2**	488395	0,17	-0,43	0,47	0,05	golgi reassembly stacking protein 2, 55kDa
**HNRNPH2**	480989	0,12	0,04	0,47	0,30	heterogeneous nuclear ribonucleoprotein H2 (H')
**CBR1**	610164	0,41	-0,52	0,47	0,06	carbonyl reductase 1
**USP7**	479854	0,43	-0,21	0,47	0,19	ubiquitin specific peptidase 7 (herpes virus-associated)
**LOC481459**	481459	-0,22	-0,12	0,48	0,20	similar to sequestosome 1
**TCEA1**	477879	0,59	3,33	0,48	0,97	transcription elongation factor A (SII), 1
**LOC475722**	475722	0,63	0,62	0,49	0,72	similar to nucleophosmin 1
**EIF5**	480442	0,71	0,30	0,49	0,46	eukaryotic translation initiation factor 5
**BZW2**	475250	0,68	0,04	0,49	0,51	basic leucine zipper and W2 domains 2
**SRP72**	403944	0,66	0,51	0,49	0,34	signal recognition particle 72kDa
**KPNA6**	487314	0,72	0,49	0,49	0,43	karyopherin alpha 6 (importin alpha 7)
**LAP3**	479081	0,27	-0,46	0,49	0,13	leucine aminopeptidase 3
**EPRS**	478962	0,55	0,02	0,50	0,60	glutamyl-prolyl-tRNA synthetase

		log2 fold change				
**Symbol**	**Entrez Id**	**4hrs p.i.**	**8 hrs p.i.**	**10 hrs p.i.**	**12 hrs p.i.**	**Name**
**CAPNS1**	611746	0,68	-0,16	0,50	0,25	calpain, small subunit 1
**EIF3E**	475070	0,58	0,13	0,51	0,38	eukaryotic translation initiation factor 3, subunit E
**SERBP1**	479535	0,93	0,71	0,51	0,83	SERPINE1 mRNA binding protein 1
**PIR**	480846	0,28	-0,70	0,51	-0,18	pirin (iron-binding nuclear protein)
**NUDT5**	478006	0,46	-0,66	0,51	0,25	nudix (nucleoside diphosphate linked moiety X)-type motif 5
**VARS**	481716	0,25	-0,21	0,51	0,41	valyl-tRNA synthetase
**CAD**	483009	0,76	0,43	0,51	0,56	carbamoyl-phosphate synthetase 2, aspartate transcarbamylase, and dihydroorotase
**CTSB**	486077	0,48	-0,21	0,52	0,35	cathepsin B
**PPP2R1B**	479444	0,41	-0,13	0,52	0,23	protein phosphatase 2, regulatory subunit A, beta
**EML4**	483048	0,45	0,02	0,52	0,49	echinoderm microtubule associated protein like 4
**CAST**	479148	0,46	0,18	0,52	0,72	calpastatin
**MCM7**	479737	0,47	-0,06	0,52	0,36	minichromosome maintenance complex component 7
**COPG**	476517	0,51	0,00	0,53	0,36	coatomer protein complex, subunit gamma
**PDCD6IP**	477015	0,53	-0,22	0,53	0,28	programmed cell death 6 interacting protein
**UBE2V1**	477262	0,42	-0,71	0,53	0,08	ubiquitin-conjugating enzyme E2 variant 1
**CAPZB**	478209	0,67	0,12	0,54	0,51	capping protein (actin filament) muscle Z-line, beta
**ARPC2**	478907	0,68	-0,07	0,54	0,33	actin related protein 2/3 complex, subunit 2, 34kDa
**QARS**	476628	0,52	-0,02	0,54	0,52	glutaminyl-tRNA synthetase
**ADK**	479253	0,57	-0,51	0,55	0,20	adenosine kinase
**IPO7**	485383	0,61	-0,12	0,55	0,45	importin 7
**EIF4G2**	476854	0,72	0,10	0,55	0,53	eukaryotic translation initiation factor 4 gamma, 2
**TLN1**	474759	0,42	-0,17	0,55	0,29	talin 1
**PCBP1**	481417	0,73	0,23	0,55	0,39	poly(rC) binding protein 1
**EIF3M**	475950	0,74	0,11	0,55	0,59	eukaryotic translation initiation factor 3, subunit M
**ARF1**	474591	0,61	0,02	0,55	0,27	ADP-ribosylation factor 1
**KPNA2**	480469	0,48	0,08	0,56	0,25	karyopherin alpha 2 (RAG cohort 1, importin alpha 1)
**KPNA3**	476909	0,71	0,20	0,56	0,42	karyopherin alpha 3 (importin alpha 4)
**NAP1L4**	483670	0,59	0,15	0,56	0,45	nucleosome assembly protein 1-like 4
**PGM1**	479545	0,37	-0,62	0,56	0,06	phosphoglucomutase 1
**NAMPT**	483267	0,49	-0,04	0,56	0,17	nicotinamide phosphoribosyltransferase
**COPS2**	478295	0,20	-0,25	0,56	0,31	COP9 constitutive photomorphogenic homolog subunit 2 (Arabidopsis)
**EZR**	484056	0,69	-0,23	0,56	0,23	ezrin
**PSAT1**	476318	0,99	-0,19	0,56	0,25	phosphoserine aminotransferase 1
**CLTC**	480578	0,63	0,43	0,57	0,53	clathrin, heavy chain (Hc)
**MTPN**	403487	0,63	-0,49	0,57	-0,22	myotrophin
**SAE1**	476425	-0,03	-0,89	0,57	0,04	SUMO1 activating enzyme subunit 1
**HSP70**	403612	0,41	0,09	0,58	0,47	heat shock protein 70
**API5**	483413	0,62	0,13	0,58	0,53	apoptosis inhibitor 5
**TCP1**	484064	0,60	0,14	0,58	0,56	t-complex 1
**TSTA3**	475116	0,82	-0,40	0,58	-0,09	tissue specific transplantation antigen P35B
**ARF4**	607073	0,56	-0,08	0,58	0,39	ADP-ribosylation factor 4

		log2 fold change				
**Symbol**	**Entrez Id**	**4hrs p.i.**	**8 hrs p.i.**	**10 hrs p.i.**	**12 hrs p.i.**	**Name**
**DDX3X**	480886	0,79	0,45	0,58	0,50	DEAD (Asp-Glu-Ala-Asp) box polypeptide 3, X-linked
**AP1B1**	486344	0,05	0,01	0,58	0,13	adaptor-related protein complex 1, beta 1 subunit
**LOC606787**	606787	0,75	0,34	0,58	0,54	similar to T-complex protein 1, zeta subunit (TCP-1-zeta) (CCT-zeta) (CCT-zeta-1)
**ACTN4**	484526	0,70	0,04	0,58	0,38	actinin, alpha 4
**HSBP1**	609752	0,64	-0,15	0,59	0,36	heat shock factor binding protein 1
**DARS**	476132	0,72	0,07	0,59	0,78	aspartyl-tRNA synthetase
**AATF**	480595	0,62	0,28	0,59	0,67	apoptosis antagonizing transcription factor
**FLNA**	481084	0,69	-0,12	0,60	0,48	filamin A, alpha
**TNNT2**	403532	0,66	-0,43	0,60	0,24	troponin T type 2 (cardiac)
**LOC475208**	475208	0,84	0,16	0,61	0,43	similar to Microtubule-associated protein RP/EB family member 1 (APC-binding protein EB1) (End-binding protein 1) (EB1)
**TXNRD1**	474536	0,17	-0,59	0,61	0,77	thioredoxin reductase 1
**HNRNPU**	480100	0,79	0,49	0,61	0,65	heterogeneous nuclear ribonucleoprotein U (scaffold attachment factor A)
**FXR1**	478642	0,69	0,45	0,61	0,49	fragile X mental retardation, autosomal homolog 1
**SMARCC1**	476640	0,70	0,12	0,61	0,81	SWI/SNF related, matrix associated, actin dependent regulator of chromatin, subfamily c, member 1
**PRKAR2A**	484774	0,41	-0,09	0,62	0,51	protein kinase, cAMP-dependent, regulatory, type II, alpha
**TAX1BP3**	491221	0,52	-0,45	0,62	0,38	Tax1 (human T-cell leukemia virus type I) binding protein 3
**VPS35**	475346	0,66	0,01	0,62	0,44	vacuolar protein sorting 35 homolog (S. cerevisiae)
**VPS26A**	479233	0,66	-0,17	0,62	0,38	vacuolar protein sorting 26 homolog A (S. pombe)
**EIF3A**	486914	0,62	0,06	0,62	0,53	eukaryotic translation initiation factor 3, subunit A
**CCT4**	482271	0,68	0,27	0,62	0,60	chaperonin containing TCP1, subunit 4 (delta)
**SEPT9**	483339	0,79	0,06	0,62	0,51	septin 9
**EIF3I**	478152	0,64	0,20	0,62	0,57	eukaryotic translation initiation factor 3, subunit I
**OLA1**	478803	0,59	-0,28	0,62	0,28	Obg-like ATPase 1
**EEF1E1**	478717	0,57	-0,05	0,63	0,68	eukaryotic translation elongation factor 1 epsilon 1
**LOC608479**	608479	0,66	-0,05	0,63	0,62	similar to Glyceraldehyde-3-phosphate dehydrogenase (GAPDH)
**LOC477309**	477309	0,82	0,34	0,63	0,38	similar to Transcription factor BTF3 homolog 3
**UCK2**	608979	0,69	-0,11	0,63	0,59	uridine-cytidine kinase 2
**UCHL5**	478958	0,64	-0,23	0,63	0,47	ubiquitin carboxyl-terminal hydrolase L5
**DDX39A**	476689	0,52	0,01	0,63	0,33	DEAD (Asp-Glu-Ala-Asp) box polypeptide 39A
**UBE2L3**	477572	0,65	-0,25	0,64	-0,05	ubiquitin-conjugating enzyme E2L 3
**MARS**	474414	0,43	-0,12	0,64	0,54	methionyl-tRNA synthetase
**EDF1**	480674	0,87	0,32	0,64	0,32	endothelial differentiation-related factor 1
**RBM14**	610639	0,67	0,14	0,64	0,58	RNA binding motif protein 14
**ANXA7**	479246	0,58	-0,42	0,64	0,16	annexin A7
**EIF3B**	479766	0,75	0,28	0,65	0,64	eukaryotic translation initiation factor 3, subunit B
**WDR1**	611070	0,78	-0,03	0,65	0,42	WD repeat domain 1
**LOC491969**	491969	0,68	-0,10	0,65	0,43	similar to CG7519-PA

		log2 fold change				
**Symbol**	**Entrez Id**	**4hrs p.i.**	**8 hrs p.i.**	**10 hrs p.i.**	**12 hrs p.i.**	**Name**
**IDH1**	478889	0,74	-0,36	0,65	0,46	isocitrate dehydrogenase 1 (NADP+), soluble
**ACACA**	491130	0,74	0,14	0,65	0,48	acetyl-CoA carboxylase alpha
**IPO4**	480260	0,44	-0,33	0,66	0,34	importin 4
**ARCN1**	479415	0,66	0,07	0,66	0,57	archain 1
**MAPK1**	477575	0,65	0,34	0,66	0,60	mitogen-activated protein kinase 1
**HRSP12**	475043	0,63	-0,24	0,66	0,43	heat-responsive protein 12
**EIF3K**	476468	0,67	0,06	0,66	0,60	eukaryotic translation initiation factor 3, subunit K
**YWHAG**	489818	0,55	-0,01	0,66	0,55	tyrosine 3-monooxygenase/tryptophan 5-monooxygenase activation protein, gamma polypeptide
**PPP1R7**	477430	0,66	-0,34	0,66	0,62	protein phosphatase 1, regulatory (inhibitor) subunit 7
**SERPINB5**	476177	0,40	-0,88	0,67	-0,16	serpin peptidase inhibitor, clade B (ovalbumin), member 5
**CAPZA1**	475857	0,64	-0,02	0,67	0,55	capping protein (actin filament) muscle Z-line, alpha 1
**GLOD4**	480640	0,61	-0,61	0,67	0,29	glyoxalase domain containing 4
**CSE1L**	477257	0,49	-0,06	0,67	0,42	CSE1 chromosome segregation 1-like (yeast)
**PSMD14**	478765	0,55	0,05	0,67	0,63	proteasome (prosome, macropain) 26S subunit, non-ATPase, 14
**NSF**	490921	0,64	-0,02	0,67	0,86	N-ethylmaleimide-sensitive factor
**GARS**	475268	0,70	-0,11	0,67	0,55	glycyl-tRNA synthetase
**PSME3**	480512	0,78	0,06	0,67	0,50	proteasome (prosome, macropain) activator subunit 3 (PA28 gamma; Ki)
**EIF3D**	608665	0,85	0,40	0,67	0,71	eukaryotic translation initiation factor 3, subunit D
**PEA15**	610113	0,85	-0,76	0,67	0,15	phosphoprotein enriched in astrocytes 15
**PSMD11**	480610	0,64	0,02	0,68	0,63	proteasome (prosome, macropain) 26S subunit, non-ATPase, 11
**PSME1**	480256	0,88	-0,16	0,68	0,45	proteasome (prosome, macropain) activator subunit 1 (PA28 alpha)
**PSMC5**	480478	0,48	-0,18	0,68	0,45	proteasome (prosome, macropain) 26S subunit, ATPase, 5
**COPB1**	476866	0,60	0,16	0,68	0,43	coatomer protein complex, subunit beta 1
**RRM1**	476823	0,54	-0,09	0,68	0,30	ribonucleotide reductase M1
**TUBA4A**	478918	0,75	0,54	0,68	0,39	tubulin, alpha 4a
**PMPCB**	475897	0,19	-0,23	0,68	-0,08	peptidase (mitochondrial processing) beta
**LOC476790**	476790	0,79	0,45	0,69	0,68	similar to chaperonin containing TCP1, subunit 3 isoform c
**PSMC4**	476459	0,51	-0,24	0,69	0,66	proteasome (prosome, macropain) 26S subunit, ATPase, 4
**ACTR2**	481396	0,69	-0,07	0,69	0,31	ARP2 actin-related protein 2 homolog (yeast)
**ACTN1**	480369	0,81	0,09	0,69	0,47	actinin, alpha 1
**KRT7**	477602	1,08	0,91	0,69	0,75	keratin 7
**MAPK1IP1L**	480329	0,51	-0,14	0,69	0,79	mitogen-activated protein kinase 1 interacting protein 1-like
**NUP85**	475913	0,48	-0,04	0,69	0,37	nucleoporin 85kDa
**DDX46**	474685	0,70	-0,10	0,69	0,61	DEAD (Asp-Glu-Ala-Asp)

Symbol	Entrez Id	log2 fold change 4hrs p.i.	8 hrs p.i.	10 hrs p.i.	12 hrs p.i.	Name
						box polypeptide 46
**AIMP1**	487893	0,44	-0,24	0,69	0,69	aminoacyl tRNA synthetase complex-interacting multifunctional protein 1
**PSMC2**	475896	0,35	-0,37	0,69	0,46	proteasome (prosome, macropain) 26S subunit, ATPase, 2
**RPS21**	485959	1,01	0,86	0,69	0,87	ribosomal protein S21
**HPRT1**	442945	0,86	-0,18	0,70	0,35	hypoxanthine phosphoribosyltransferase 1
**FLNB**	484713	0,78	-0,09	0,70	0,55	filamin B, beta
**CAND1**	474437	0,66	-0,02	0,70	0,48	cullin-associated and neddylation-dissociated 1
**PSMA5**	490123	0,74	0,01	0,70	0,63	proteasome (prosome, macropain) subunit, alpha type, 5
**PSMD5**	474703	0,45	-0,22	0,70	0,30	proteasome (prosome, macropain) 26S subunit, non-ATPase, 5
**DNM1L**	477649	0,58	-0,35	0,70	0,57	dynamin 1-like
**LOC475101**	475101	0,76	-0,17	0,71	0,31	similar to L-lactate dehydrogenase B chain (LDH-B) (LDH heart subunit) (LDH-H)
**MAP2K3**	489547	0,71	-0,10	0,71	0,43	mitogen-activated protein kinase kinase 3
**MAT2A**	475770	0,89	0,24	0,71	0,47	methionine adenosyltransferase II, alpha
**COMMD9**	608575	0,89	0,37	0,71	0,49	COMM domain containing 9
**APEX1**	482558	0,81	0,26	0,71	0,71	APEX nuclease (multifunctional DNA repair enzyme) 1
**EIF3C**	479795	0,74	0,20	0,71	0,71	eukaryotic translation initiation factor 3, subunit C
**ASNS**	475240	0,90	0,28	0,71	0,59	asparagine synthetase (glutamine-hydrolyzing)
**HSPA8**	479406	0,73	0,29	0,71	0,64	heat shock 70kDa protein 8
**HDAC2**	475035	0,64	0,29	0,72	0,67	histone deacetylase 2
**PABPN1**	490611	0,74	0,57	0,72	0,82	poly(A) binding protein, nuclear 1
**PTGR1**	474802	0,65	-0,21	0,72	0,41	prostaglandin reductase 1
**PDCD6**	609549	0,87	-0,14	0,72	0,14	programmed cell death 6
**G3BP1**	479322	0,91	0,62	0,73	0,79	GTPase activating protein (SH3 domain) binding protein 1
**CALM2**	474584	0,89	0,35	0,73	0,66	calmodulin 2 (phosphorylase kinase, delta)
**DIABLO**	477463	0,68	0,21	0,73	0,84	diablo, IAP-binding mitochondrial protein
**U2AF1**	478422	0,44	0,42	0,73	0,52	U2 small nuclear RNA auxiliary factor 1
**BZW1**	478865	0,99	0,18	0,73	0,62	basic leucine zipper and W2 domains 1
**TNPO3**	482263	0,57	0,12	0,73	0,40	transportin 3
**UGDH**	479107	0,37	-0,22	0,73	0,57	UDP-glucose 6-dehydrogenase
**HSPH1**	477322	0,70	0,05	0,73	0,63	heat shock 105kDa/110kDa protein 1
**G3BP2**	478429	0,81	0,42	0,73	0,76	GTPase activating protein (SH3 domain) binding protein 2
**LOC486736**	486736	0,68	-0,29	0,73	0,13	similar to chromosome 12 open reading frame 5
**KHDRBS1**	487316	0,59	-0,05	0,74	0,55	KH domain containing, RNA binding, signal transduction associated 1
**SF3A1**	477538	0,49	-0,27	0,74	0,41	splicing factor 3a, subunit 1, 120kDa
**VPS29**	477477	0,45	-0,13	0,74	0,61	vacuolar protein sorting 29 homolog (S. cerevisiae)
**SPR**	483118	0,67	-0,45	0,74	0,27	sepiapterin reductase (7,8-

		log2 fold change				
**Symbol**	**Entrez Id**	**4hrs p.i.**	**8 hrs p.i.**	**10 hrs p.i.**	**12 hrs p.i.**	**Name**
						dihydrobiopterin:NADP+ oxidoreductase)
**GSPT1**	479846	0,94	0,08	0,74	0,63	G1 to S phase transition 1
**CASP8**	488473	1,01	-0,05	0,74	0,65	caspase 8, apoptosis-related cysteine peptidase
**PSMD2**	478654	0,64	-0,20	0,74	0,44	proteasome (prosome, macropain) 26S subunit, non-ATPase, 2
**CCT2**	474445	0,79	0,27	0,74	0,77	chaperonin containing TCP1, subunit 2 (beta)
**PSMD8**	476470	0,46	-0,51	0,74	0,49	proteasome (prosome, macropain) 26S subunit, non-ATPase, 8
**PFKP**	478019	0,83	0,25	0,74	0,68	phosphofructokinase, platelet
**ARPC5L**	612856	0,89	0,43	0,75	0,66	actin related protein 2/3 complex, subunit 5-like
**ALDH1L1**	476506	0,43	-0,14	0,75	0,57	aldehyde dehydrogenase 1 family, member L1
**RUVBL1**	476512	0,67	0,29	0,75	0,57	RuvB-like 1 (E. coli)
**TWF1**	486600	0,81	0,06	0,75	0,52	twinfilin, actin-binding protein, homolog 1 (Drosophila)
**PGM3**	474981	0,70	-0,31	0,75	0,15	phosphoglucomutase 3
**ZW10**	479433	0,86	0,52	0,75	0,89	ZW10, kinetochore associated, homolog (Drosophila)
**CSTF2**	492015	0,40	-0,25	0,75	0,38	cleavage stimulation factor, 3' pre-RNA, subunit 2, 64kDa
**NUTF2**	479681	0,55	-0,34	0,75	0,40	nuclear transport factor 2
**PLA2G4A**	480048	0,78	-0,03	0,75	0,53	phospholipase A2, group IVA (cytosolic, calcium-dependent)
**SHMT1**	489536	0,47	-0,17	0,75	0,08	serine hydroxymethyltransferase 1 (soluble)
**CCT5**	606873	0,86	0,28	0,76	0,70	chaperonin containing TCP1, subunit 5 (epsilon)
**DDX42**	480479	0,60	0,29	0,76	0,56	DEAD (Asp-Glu-Ala-Asp) box polypeptide 42
**NUP93**	478119	0,59	0,03	0,76	0,50	nucleoporin 93kDa
**RSU1**	477993	0,53	-0,23	0,76	0,39	Ras suppressor protein 1
**PSME2**	480258	0,86	0,04	0,76	0,64	proteasome (prosome, macropain) activator subunit 2 (PA28 beta)
**CLIC1**	474847	0,69	-0,25	0,76	0,22	chloride intracellular channel 1
**SUMO2**	474963	0,68	-0,40	0,76	0,48	SMT3 suppressor of mif two 3 homolog 2 (S. cerevisiae)
**ACTR3**	403702	0,91	0,16	0,77	0,64	ARP3 actin-related protein 3 homolog (yeast)
**EIF4A2**	488118	0,89	0,13	0,77	0,44	eukaryotic translation initiation factor 4A2
**UTP14A**	492128	0,85	-0,39	0,77	0,89	UTP14, U3 small nucleolar ribonucleoprotein, homolog A (yeast)
**SPTAN1**	403484	0,83	0,34	0,77	0,71	spectrin, alpha, non-erythrocytic 1 (alpha-fodrin)
**CTH**	479991	0,66	-0,37	0,77	0,38	cystathionase (cystathionine gamma-lyase)
**ACTB**	403580	0,82	0,15	0,78	0,60	actin, beta
**ARPC3**	477474	0,77	-0,03	0,78	0,52	actin related protein 2/3 complex, subunit 3, 21kDa
**TPR**	480045	0,58	-0,06	0,78	0,55	translocated promoter region (to activated MET oncogene)
**RUVBL2**	476418	0,79	0,34	0,78	0,73	RuvB-like 2 (E. coli)
**PSMC1**	478703	0,58	0,14	0,78	0,72	proteasome (prosome, macropain) 26S subunit, ATPase, 1
**UPP1**	480772	1,02	-0,21	0,78	0,48	uridine phosphorylase 1

		log2 fold change				
**Symbol**	**Entrez Id**	**4hrs p.i.**	**8 hrs p.i.**	**10 hrs p.i.**	**12 hrs p.i.**	**Name**
**DUT**	609526	0,36	-0,50	0,78	0,44	deoxyuridine triphosphatase
**ANXA4**	606756	0,89	-0,11	0,78	0,33	annexin A4
**EEF1B2**	478881	0,95	0,41	0,79	0,83	eukaryotic translation elongation factor 1 beta 2
**cOR2W9**	488336	0,89	0,02	0,79	0,54	cOR2W9 olfactory receptor family 2 subfamily W-like
**FDPS**	480129	0,76	-0,26	0,79	0,42	farnesyl diphosphate synthase
**DPYSL2**	486107	0,67	-0,23	0,79	0,66	dihydropyrimidinase-like 2
**ARPC4**	476540	0,94	0,18	0,79	0,61	actin related protein 2/3 complex, subunit 4, 20kDa
**CACYBP**	480062	0,82	-0,05	0,80	0,59	calcyclin binding protein
**UFM1**	477297	0,73	-0,09	0,80	0,28	ubiquitin-fold modifier 1
**HSPA4**	474680	0,75	0,06	0,80	0,69	heat shock 70kDa protein 4
**CNDP2**	476166	0,99	0,04	0,80	0,73	CNDP dipeptidase 2 (metallopeptidase M20 family)
**PSMC6**	478522	0,72	0,05	0,81	0,62	proteasome (prosome, macropain) 26S subunit, ATPase, 6
**PSMD12**	480465	0,77	0,11	0,81	0,74	proteasome (prosome, macropain) 26S subunit, non-ATPase, 12
**PARK7**	479595	0,63	-0,36	0,81	0,17	parkinson protein 7
**ANXA2**	403435	1,11	0,69	0,81	0,72	annexin A2
**NPEPPS**	480538	0,79	-0,19	0,81	0,43	aminopeptidase puromycin sensitive
**LOC489680**	489680	1,09	-0,29	0,81	0,48	similar to coactosin-like 1
**RBBP7**	480854	0,85	0,33	0,82	0,83	retinoblastoma binding protein 7
**CFL2**	490649	0,50	-0,35	0,83	0,48	cofilin 2 (muscle)
**LOC609233**	609233	1,10	0,43	0,83	0,75	similar to dynein, cytoplasmic, light peptide
**IPO5**	485528	0,82	0,12	0,83	0,71	importin 5
**PSMA6**	480290	0,84	0,07	0,83	0,77	proteasome (prosome, macropain) subunit, alpha type, 6
**UBA2**	476490	0,56	-0,21	0,83	0,68	ubiquitin-like modifier activating enzyme 2
**MBNL1**	477116	0,75	0,28	0,83	0,97	muscleblind-like (Drosophila)
**KRT17**	490977	1,22	0,24	0,84	0,48	keratin 17
**SNRPA**	476454	0,79	0,16	0,84	0,45	small nuclear ribonucleoprotein polypeptide A
**ATP6V1B2**	486137	0,67	0,11	0,84	0,64	ATPase, H+ transporting, lysosomal 56/58kDa, V1 subunit B2
**PRPS2**	476855	1,08	0,68	0,84	0,88	phosphoribosyl pyrophosphate synthetase 2
**VIM**	477991	0,84	0,55	0,85	0,60	vimentin
**PCNA**	477166	0,95	0,24	0,85	0,72	proliferating cell nuclear antigen
**RANBP1**	477563	0,71	-0,11	0,85	0,46	RAN binding protein 1
**PCMT1**	476242	0,60	-0,21	0,85	0,51	protein-L-isoaspartate (D-aspartate) O-methyltransferase
**HNRNPK**	476309	0,90	0,31	0,85	0,85	heterogeneous nuclear ribonucleoprotein K
**KHSRP**	485022	0,91	0,36	0,85	0,75	KH-type splicing regulatory protein
**CNN3**	479937	0,61	-0,39	0,86	0,38	calponin 3, acidic
**MTHFD1**	480352	0,87	-0,02	0,86	0,57	methylenetetrahydrofolate dehydrogenase (NADP+ dependent) 1, methenyltetrahydrofolate cyclohydrolase, formyltetrahydrofolate synthetase
**ARHGAP1**	483631	0,86	-0,42	0,86	0,29	Rho GTPase activating

		log2 fold change				
**Symbol**	**Entrez Id**	**4hrs p.i.**	**8 hrs p.i.**	**10 hrs p.i.**	**12 hrs p.i.**	**Name**
						protein 1
**TPI1**	477711	0,90	-0,09	0,86	0,60	triosephosphate isomerase 1
**ATIC**	488513	0,92	-0,09	0,86	0,60	5-aminoimidazole-4-carboxamide ribonucleotide formyltransferase/IMP cyclohydrolase
**ERH**	480371	0,97	0,55	0,86	0,84	enhancer of rudimentary homolog (Drosophila)
**PRDX5**	476032	0,75	-0,28	0,86	0,36	peroxiredoxin 5
**EEF1A1**	403506	0,99	0,36	0,86	0,70	eukaryotic translation elongation factor 1 alpha 1
**CBX3**	482015	0,89	0,39	0,86	0,76	chromobox homolog 3
**CAP1**	475317	0,87	0,07	0,87	0,79	CAP, adenylate cyclase-associated protein 1 (yeast)
**PSMA4**	475132	0,77	-0,14	0,87	0,77	proteasome (prosome, macropain) subunit, alpha type, 4
**BLVRB**	476456	0,56	-0,41	0,87	0,12	biliverdin reductase B (flavin reductase (NADPH))
**SARS**	490124	0,58	-0,39	0,87	0,60	seryl-tRNA synthetase
**KATNAL2**	490465	0,46	-0,23	0,87	0,51	katanin p60 subunit A-like 2
**MDH1**	474614	1,04	0,09	0,87	0,63	malate dehydrogenase 1, NAD (soluble)
**COPS4**	478455	0,35	-0,42	0,88	0,48	COP9 constitutive photomorphogenic homolog subunit 4 (Arabidopsis)
**GLRX3**	477869	0,91	0,03	0,88	0,44	glutaredoxin 3
**PSMB5**	480246	0,93	0,09	0,88	0,83	proteasome (prosome, macropain) subunit, beta type, 5
**FASN**	483378	0,90	0,07	0,88	0,59	fatty acid synthase
**FUBP1**	490201	0,73	0,02	0,88	0,48	far upstream element (FUSE) binding protein 1
**CCT8**	478399	0,91	0,35	0,88	0,86	chaperonin containing TCP1, subunit 8 (theta)
**OTUB1**	476039	0,96	-0,01	0,88	0,51	OTU domain, ubiquitin aldehyde binding 1
**HSPB1**	403979	0,37	-0,35	0,88	0,43	heat shock 27kDa protein 1
**ADSL**	474499	0,88	-0,03	0,89	0,59	adenylosuccinate lyase
**KRT8**	486513	1,25	0,97	0,89	0,96	keratin 8
**DDB1**	476067	0,73	-0,04	0,89	0,68	damage-specific DNA binding protein 1, 127kDa
**PRMT1**	476411	0,97	0,67	0,89	0,79	protein arginine methyltransferase 1
**S100A6**	480143	1,01	-0,25	0,89	0,47	S100 calcium binding protein A6
**PDAP1**	489861	1,10	0,29	0,89	1,04	PDGFA associated protein 1
**DDT**	607589	0,93	-0,29	0,89	0,51	D-dopachrome tautomerase
**PTGES3**	612760	0,92	-0,14	0,89	0,71	prostaglandin E synthase 3 (cytosolic)
**DCTN2**	474415	0,72	0,11	0,89	0,97	dynactin 2 (p50)
**NME2**	480559	1,04	0,24	0,90	0,68	non-metastatic cells 2, protein (NM23B) expressed in
**LOC607890**	607890	1,15	0,36	0,90	0,86	similar to D-3-phosphoglycerate dehydrogenase (3-PGDH)
**NUDC**	487349	0,68	-0,25	0,90	0,60	nuclear distribution gene C homolog (A. nidulans)
**CDC37**	484955	0,74	-0,02	0,90	0,90	cell division cycle 37 homolog (S. cerevisiae)
**ANXA3**	478447	1,11	0,16	0,90	0,66	annexin A3
**LDHA**	476882	1,03	0,22	0,90	0,73	lactate dehydrogenase A
**PSMD6**	484700	0,83	0,21	0,90	0,77	proteasome (prosome, macropain) 26S subunit, non-ATPase, 6
**PSMC3**	475980	0,68	0,06	0,90	0,60	proteasome (prosome, macropain) 26S subunit, ATPase, 3

		log2 fold change				
**Symbol**	**Entrez Id**	**4hrs p.i.**	**8 hrs p.i.**	**10 hrs p.i.**	**12 hrs p.i.**	**Name**
**PSMA1**	476867	0,87	-0,05	0,91	0,82	proteasome (prosome, macropain) subunit, alpha type, 1
**PAICS**	475150	0,76	-0,12	0,91	0,67	phosphoribosylaminoimidazole carboxylase, phosphoribosylaminoimidazole succinocarboxamide synthetase
**PUF60**	607245	0,61	-0,08	0,91	0,49	poly-U binding splicing factor 60KDa
**CAPN2**	480118	1,11	0,04	0,91	0,81	calpain 2, (m/II) large subunit
**PPM1G**	475703	0,60	-0,06	0,91	0,72	protein phosphatase, Mg2+/Mn2+ dependent, 1G
**PSMA7**	404305	0,81	0,09	0,91	0,69	proteasome (prosome, macropain) subunit, alpha type, 7
**NASP**	475373	0,79	0,23	0,91	0,90	nuclear autoantigenic sperm protein (histone-binding)
**PSMA2**	475870	0,82	0,12	0,92	0,87	proteasome (prosome, macropain) subunit, alpha type, 2
**NDRG1**	482049	1,03	-0,02	0,92	0,35	N-myc downstream regulated 1
**PDLIM5**	478482	0,84	-0,03	0,92	0,27	PDZ and LIM domain 5
**KRT18**	477601	1,29	1,06	0,92	1,06	keratin 18
**PLEC**	482083	1,17	0,68	0,92	0,79	plectin
**RNMT**	476200	0,77	0,22	0,92	0,75	RNA (guanine-7-) methyltransferase
**PGD**	478236	0,77	-0,07	0,92	0,59	phosphogluconate dehydrogenase
**KPNB1**	491042	0,88	0,25	0,93	0,82	karyopherin (importin) beta 1
**HMGA1**	442946	0,77	0,33	0,93	0,38	high mobility group AT-hook 1
**TSNAX**	479203	0,60	-0,19	0,93	0,56	translin-associated factor X
**PPP1CA**	403609	0,92	0,48	0,93	0,81	protein phosphatase 1, catalytic subunit, alpha isozyme
**SF3B2**	476015	0,72	0,18	0,94	0,60	splicing factor 3b, subunit 2, 145kDa
**G6PD**	481088	0,71	-0,18	0,94	0,47	glucose-6-phosphate dehydrogenase
**GART**	487740	0,82	0,05	0,94	0,66	phosphoribosylglycinamide formyltransferase, phosphoribosylglycinamide synthetase, phosphoribosylaminoimidazole synthetase
**PPA1**	479238	0,80	-0,28	0,94	0,72	pyrophosphatase (inorganic) 1
**LOC474798**	474798	0,95	3.81332562604872e-05	0,95	0,26	similar to Thioredoxin (ATL-derived factor) (ADF) (Surface associated sulphydryl protein) (SASP)
**THRAP3**	482476	0,95	0,43	0,95	0,86	thyroid hormone receptor associated protein 3
**ACOT7**	479589	0,84	-0,30	0,95	0,36	acyl-CoA thioesterase 7
**LOC475852**	475852	1,10	0,01	0,95	0,56	similar to Calgizzarin (S100 calcium-binding protein A11) (S100C protein) (MLN 70)
**NQO1**	610935	0,90	-0,27	0,95	0,43	NAD(P)H dehydrogenase, quinone 1
**USP5**	486718	0,68	-0,40	0,95	0,56	ubiquitin specific peptidase 5 (isopeptidase T)
**CPNE1**	477213	0,80	-0,21	0,96	0,57	copine I
**ALDH1A1**	476323	1,16	0,11	0,96	0,84	aldehyde dehydrogenase 1 family, member A1
**SF3A3**	475326	0,93	0,27	0,96	0,85	splicing factor 3a, subunit 3, 60kDa
**TALDO1**	475937	1,20	0,08	0,96	0,83	transaldolase 1
**LOC476570**	476570	1,01	0,45	0,96	0,93	similar to Elongation factor 1-gamma (EF-1-gamma)

		log2 fold change				
**Symbol**	**Entrez Id**	**4hrs p.i.**	**8 hrs p.i.**	**10 hrs p.i.**	**12 hrs p.i.**	**Name**
						(eEF-1B gamma)
**TCEB1**	477914	0,73	0,09	0,96	0,89	transcription elongation factor B (SIII), polypeptide 1 (15kDa, elongin C)
**CA2**	477928	0,83	0,12	0,96	0,76	carbonic anhydrase II
**UMPS**	478593	0,95	0,07	0,96	0,85	uridine monophosphate synthetase
**USP10**	479625	0,95	0,32	0,96	0,79	ubiquitin specific peptidase 10
**GMPS**	477123	0,70	0,00	0,97	0,73	guanine monphosphate synthetase
**TCEB2**	479873	1,15	0,29	0,97	0,81	transcription elongation factor B (SIII), polypeptide 2 (18kDa, elongin B)
**IMPDH2**	476630	0,88	0,58	0,97	1,04	IMP (inosine 5'-monophosphate) dehydrogenase 2
**SEC31A**	478453	0,51	-0,18	0,97	0,72	SEC31 homolog A (S. cerevisiae)
**PSMD13**	475934	0,83	0,23	0,97	0,85	proteasome (prosome, macropain) 26S subunit, non-ATPase, 13
**SKP1**	474682	0,90	0,32	0,97	0,62	S-phase kinase-associated protein 1
**CAB39**	477403	1,04	-0,02	0,98	0,64	calcium binding protein 39
**CD2AP**	474928	0,67	-0,32	0,98	0,47	CD2-associated protein
**RBM25**	610314	0,22	-0,22	0,98	0,39	RNA binding motif protein 25
**SEPHS1**	477999	0,91	0,24	0,98	0,84	selenophosphate synthetase 1
**AK2**	478145	0,58	0,07	0,99	0,76	adenylate kinase 2
**CFL1**	476022	1,02	-0,20	0,99	0,40	cofilin 1 (non-muscle)
**PSMB4**	475848	0,83	0,18	0,99	1,07	proteasome (prosome, macropain) subunit, beta type, 4
**LOC483608**	483608	1,07	0,33	0,99	0,90	similar to heat shock 70kD protein binding protein
**PGK1**	480964	1,02	0,10	1,00	0,71	phosphoglycerate kinase 1
**PSMD7**	479671	0,82	0,06	1,00	0,80	proteasome (prosome, macropain) 26S subunit, non-ATPase, 7
**ME1**	403709	0,61	-0,07	1,01	0,60	malic enzyme 1, NADP(+)-dependent, cytosolic
**ANXA5**	476094	1,16	-0,25	1,01	0,54	annexin A5
**COPS3**	479528	0,88	0,03	1,01	0,78	COP9 constitutive photomorphogenic homolog subunit 3 (Arabidopsis)
**PPID**	475481	1,01	-0,09	1,01	0,67	peptidylprolyl isomerase D
**PRDX6**	480069	0,99	-0,24	1,01	0,51	peroxiredoxin 6
**LUC7L2**	475529	1,12	0,43	1,01	0,90	LUC7-like 2 (S. cerevisiae)
**FKBP4**	477726	0,93	0,04	1,02	0,84	FK506 binding protein 4, 59kDa
**MAP2K1**	478347	0,79	0,13	1,02	0,61	mitogen-activated protein kinase kinase 1
**UBA1**	480896	1,10	0,07	1,02	0,85	ubiquitin-like modifier activating enzyme 1
**EEF1D**	475115	1,17	0,57	1,02	1,00	eukaryotic translation elongation factor 1 delta (guanine nucleotide exchange protein)
**CPSF6**	474441	0,77	0,46	1,02	0,81	cleavage and polyadenylation specific factor 6, 68kDa
**LOC474987**	474987	1,02	0,33	1,02	1,13	similar to Proteasome subunit beta type 3 (Proteasome theta chain) (Proteasome chain 13) (Proteasome component C10-II)
**FABP3**	478156	0,73	-0,74	1,02	-0,07	fatty acid binding protein 3, muscle and heart

		log2 fold change				
**Symbol**	**Entrez Id**	**4hrs p.i.**	**8 hrs p.i.**	**10 hrs p.i.**	**12 hrs p.i.**	**Name**
						(mammary-derived growth inhibitor)
**SEC23A**	480295	0,66	0,24	1,02	0,44	Sec23 homolog A (S. cerevisiae)
**ENO1**	479597	1,06	0,08	1,02	0,73	enolase 1, (alpha)
**ATP6V1G2**	474840	1,08	0,46	1,02	1,03	ATPase, H+ transporting, lysosomal 13kDa, V1 subunit G2
**ATP6V1A**	487981	0,89	0,17	1,03	0,83	ATPase, H+ transporting, lysosomal 70kDa, V1 subunit A
**PSMD3**	491018	0,76	0,02	1,03	0,71	proteasome (prosome, macropain) 26S subunit, non-ATPase, 3
**ACAT2**	484063	0,98	-0,19	1,03	0,54	acetyl-CoA acetyltransferase 2
**YWHAE**	480645	1,08	0,40	1,04	0,87	tyrosine 3-monooxygenase/tryptophan 5-monooxygenase activation protein, epsilon polypeptide
**LOC477951**	477951	0,91	-0,04	1,04	0,66	similar to suppressor of G2 allele of SKP1
**SPTBN1**	403743	1,05	0,55	1,04	0,95	spectrin, beta, non-erythrocytic 1
**LOC478521**	478521	1,23	0,46	1,04	0,96	similar to 14-3-3 protein theta (14-3-3 protein tau)
**ANXA11**	479259	1,09	0,01	1,04	0,67	annexin A11
**PSMB2**	475338	0,99	0,21	1,05	1,03	proteasome (prosome, macropain) subunit, beta type, 2
**DCXR**	475926	0,88	-0,02	1,05	0,62	dicarbonyl/L-xylulose reductase
**GDI2**	403818	1,00	0,08	1,05	0,74	GDP dissociation inhibitor 2
**PSMA3**	480338	1,03	0,32	1,05	1,03	proteasome (prosome, macropain) subunit, alpha type, 3
**RNH1**	483402	0,93	-0,13	1,05	0,68	ribonuclease/angiogenin inhibitor 1
**SRRM2**	609638	0,70	0,02	1,06	0,81	serine/arginine repetitive matrix 2
**EIF4B**	477600	1,23	0,77	1,06	1,06	eukaryotic translation initiation factor 4B
**CTTN**	610283	1,10	0,33	1,06	0,86	cortactin
**PSMB1**	475040	0,97	0,18	1,06	1,08	proteasome (prosome, macropain) subunit, beta type, 1
**AK1**	480712	0,69	-0,46	1,06	0,24	adenylate kinase 1
**ESD**	607116	0,52	-0,60	1,06	0,42	esterase D
**HSP90AB1**	474919	0,98	0,21	1,07	0,92	heat shock protein 90kDa alpha (cytosolic), class B member 1
**TXNL1**	483970	0,96	-0,15	1,07	0,65	thioredoxin-like 1
**ATOX1**	403713	1,13	-0,30	1,07	0,02	ATX1 antioxidant protein 1 homolog (yeast)
**SRI**	475220	1,27	0,08	1,07	0,87	sorcin
**PKM2**	403874	1,08	0,26	1,07	1,01	pyruvate kinase, muscle
**TIMM8A**	480986	1,01	0,85	1,07	1,07	translocase of inner mitochondrial membrane 8 homolog A (yeast)
**EEF2**	476744	1,15	0,31	1,08	0,84	eukaryotic translation elongation factor 2
**CDK6**	609920	0,74	-0,15	1,08	0,01	cyclin-dependent kinase 6
**NME1**	476767	0,99	0,12	1,08	0,69	non-metastatic cells 1, protein (NM23A) expressed in
**STIP1**	474837	1,09	0,32	1,08	1,03	stress-induced-phosphoprotein 1
**TTLL12**	474474	0,90	-0,01	1,08	0,78	tubulin tyrosine ligase-like family, member 12
**CRYAB**	479441	0,85	0,19	1,08	0,91	crystallin, alpha B

		log2 fold change				
**Symbol**	**Entrez Id**	**4hrs p.i.**	**8 hrs p.i.**	**10 hrs p.i.**	**12 hrs p.i.**	**Name**
**LOC486122**	486122	0,67	0,19	1,09	0,62	similar to p30 DBC protein
**YWHAB**	477237	1,09	0,44	1,09	0,99	tyrosine 3-monooxygenase/tryptophan 5-monooxygenase activation protein, beta polypeptide
**LOC480151**	480151	0,87	0,27	1,09	0,89	similar to CG14299-PA, isoform A
**RAN**	442976	1,04	0,14	1,09	0,89	RAN, member RAS oncogene family
**BCAT1**	486633	1,03	-0,03	1,10	0,71	branched chain amino-acid transaminase 1, cytosolic
**ATP6V1E1**	477740	0,75	-0,06	1,11	0,77	ATPase, H+ transporting, lysosomal 31kDa, V1 subunit E1
**LOC607013**	607013	1,00	0,29	1,11	1,04	similar to Coatomer zeta-1 subunit (Zeta-1 coat protein) (Zeta-1 COP)
**CAPG**	483082	1,29	0,00	1,11	0,71	capping protein (actin filament), gelsolin-like
**IPO9**	480004	0,97	0,08	1,12	0,93	importin 9
**LASP1**	608624	1,15	0,09	1,12	0,76	LIM and SH3 protein 1
**ANXA8**	479270	1,11	-0,10	1,13	0,57	annexin A8
**VCP**	481590	0,86	0,34	1,13	0,99	valosin containing protein
**EIF3G**	612971	1,15	0,44	1,13	0,95	eukaryotic translation initiation factor 3, subunit G
**GSPT2**	480921	0,71	-0,07	1,13	0,84	G1 to S phase transition 2
**TBCA**	479173	0,80	-0,46	1,13	0,44	tubulin folding cofactor A
**DGKA**	474393	0,98	-0,03	1,13	0,93	diacylglycerol kinase, alpha 80kDa
**S100A4**	403787	1,05	-0,79	1,13	0,09	S100 calcium binding protein A4
**PGLS**	610090	0,67	-0,47	1,13	0,20	6-phosphogluconolactonase
**UBQLN1**	476312	0,99	0,04	1,14	0,88	ubiquilin 1
**PPP2CA**	403608	1,02	0,39	1,14	0,89	protein phosphatase 2, catalytic subunit, alpha isozyme
**PEBP1**	477501	0,96	-0,22	1,14	0,37	phosphatidylethanolamine binding protein 1
**PNP**	475393	1,33	0,15	1,14	0,88	purine nucleoside phosphorylase
**GDI1**	403819	1,09	-0,03	1,14	0,78	GDP dissociation inhibitor 1
**ISG15**	479575	1,32	0,10	1,14	1,53	ISG15 ubiquitin-like modifier
**LOC476784**	476784	1,00	0,07	1,15	0,62	similar to ubiquitin-conjugating enzyme E2N
**ANXA1**	476322	1,34	0,10	1,15	0,73	annexin A1
**EIF4H**	607749	1,28	0,56	1,15	0,98	eukaryotic translation initiation factor 4H
**PRDX2**	484926	1,11	0,33	1,16	0,97	peroxiredoxin 2
**HMGCS1**	479344	1,15	0,51	1,16	0,68	3-hydroxy-3-methylglutaryl-CoA synthase 1 (soluble)
**CORO1B**	476007	1,13	0,32	1,16	0,88	coronin, actin binding protein, 1B
**VASP**	403936	1,13	0,08	1,16	0,89	vasodilator-stimulated phosphoprotein
**PRKAR1A**	480459	1,01	0,07	1,16	0,81	protein kinase, cAMP-dependent, regulatory, type I, alpha (tissue specific extinguisher 1)
**BPNT1**	608525	0,97	0,03	1,17	0,72	3'(2'), 5'-bisphosphate nucleotidase 1
**USP14**	480182	0,99	0,24	1,17	0,86	ubiquitin specific peptidase 14 (tRNA-guanine transglycosylase)
**TPT1**	476924	1,34	0,31	1,17	0,91	tumor protein, translationally-controlled 1
**EIF4A1**	479485	1,23	0,60	1,18	0,98	eukaryotic translation initiation factor 4A1
**PDCD5**	476499	0,89	-0,24	1,18	0,90	programmed cell death 5
**CSTB**	478424	1,33	0,01	1,19	0,61	cystatin B (stefin B)

		log2 fold change				
**Symbol**	**Entrez Id**	**4hrs p.i.**	**8 hrs p.i.**	**10 hrs p.i.**	**12 hrs p.i.**	**Name**
**TKT**	476588	1,22	0,30	1,20	1,02	transketolase
**UBXN1**	476055	0,91	-0,12	1,21	0,84	UBX domain protein 1
**AHSA1**	480402	0,67	-0,02	1,22	0,98	AHA1, activator of heat shock 90kDa protein ATPase homolog 1 (yeast)
**SF1**	476031	0,74	0,50	1,22	0,85	splicing factor 1
**ALDOC**	480622	0,75	-0,11	1,22	0,82	aldolase C, fructose-bisphosphate
**YWHAZ**	475864	1,27	0,50	1,23	1,03	tyrosine 3-monooxygenase/tryptophan 5-monooxygenase activation protein, zeta polypeptide
**HINT1**	474667	1,17	0,07	1,23	0,83	histidine triad nucleotide binding protein 1
**PGAM1**	477786	1,04	-0,04	1,24	0,71	phosphoglycerate mutase 1 (brain)
**GSTO1**	477813	1,29	-0,08	1,24	0,75	glutathione S-transferase omega 1
**DBNL**	607412	0,87	0,15	1,24	0,88	drebrin-like
**PREP**	481945	0,89	-0,18	1,25	0,78	prolyl endopeptidase
**TFG**	487954	1,05	-0,13	1,25	1,13	TRK-fused gene
**LOC475191**	475191	1,20	-0,07	1,26	0,74	similar to Glutathione S-transferase P (GST 7-7) (Chain 7) (GST class-pi)
**EML2**	476434	0,65	-0,31	1,28	0,40	echinoderm microtubule associated protein like 2
**NOLC1**	609496	1,04	0,32	1,29	1,05	nucleolar and coiled-body phosphoprotein 1
**TAGLN2**	610210	1,37	0,20	1,29	0,93	transgelin 2
**TPM1**	478332	1,53	0,65	1,33	1,33	tropomyosin 1 (alpha)
**STMN1**	478175	1,46	-0,10	1,34	0,87	stathmin 1
**ARHGDIA**	475924	1,21	0,23	1,35	0,84	Rho GDP dissociation inhibitor (GDI) alpha
**SERPINB9**	488192	1,36	0,35	1,37	1,56	serpin peptidase inhibitor, clade B (ovalbumin), member 9
**LOC476719**	476719	1,21	0,25	1,38	1,09	similar to Fructose-bisphosphate aldolase A (Muscle-type aldolase) (Lung cancer antigen NY-LU-1)
**SFN**	487351	1,42	0,43	1,39	1,07	stratifin
**NDUFA8**	480741	-0,76	-0,64	1,39	-0,64	NADH dehydrogenase (ubiquinone) 1 alpha subcomplex, 8, 19kDa
**DBI**	476115	1,61	0,17	1,43	0,76	diazepam binding inhibitor (GABA receptor modulator, acyl-CoA binding protein)
**HNRNPK**	480979	1,53	0,98	1,45	1,56	heterogeneous nuclear ribonucleoprotein K
**PPIA**	403581	1,70	0,63	1,45	1,10	peptidylprolyl isomerase A (cyclophilin A)
**GLTP**	612989	1,41	0,52	1,46	0,75	glycolipid transfer protein
**SRRM1**	478185	1,40	0,40	1,46	1,15	serine/arginine repetitive matrix 1
**LOC613005**	613005	1,60	0,59	1,48	1,00	similar to thymosin, beta 10
**TPM4**	609879	1,60	0,44	1,48	1,33	tropomyosin 4
**SF3A2**	612188	0,98	0,41	1,50	1,14	splicing factor 3a, subunit 2, 66kDa
**AHCY**	477198	1,08	-0,02	1,51	1,12	adenosylhomocysteinase
**LGALS3**	404021	1,71	0,74	1,52	1,00	lectin, galactoside-binding, soluble, 3
**PAFAH1B2**	479425	1,41	0,23	1,54	1,06	platelet-activating factor acetylhydrolase 1b, catalytic subunit 2 (30kDa)
**APRT**	479615	1,24	-0,25	1,56	0,84	adenine phosphoribosyltransferase
**ANP32A**	403534	1,67	0,74	1,68	1,68	acidic (leucine-rich) nuclear phosphoprotein 32 family, member A
**PKP3**	483399	0,27	-0,01	-	-0,27	plakophilin 3

		log2 fold change				
**Symbol**	**Entrez Id**	**4hrs p.i.**	**8 hrs p.i.**	**10 hrs p.i.**	**12 hrs p.i.**	**Name**
				3.486461914<br>79722e-05		
