# Supplementary material for: Alteration of Protein Levels during Influenza Virus H1N1 Infection in Host Cells: A Proteomic Survey of Host and Virus Reveals Differential Dynamics
Source: PLoS One. 2014 Apr 9;9(4):e94257. doi: 10.1371/journal.pone.0094257 (PMC3981805; doi:10.1371/journal.pone.0094257)
Supplement: Table S2 — Z-scores for all significant GO terms in the four quartiles. (PDF) [file pone.0094257.s006.pdf]

Table S2: Z-scores for all significant GO terms in the four quartiles.

GO Biological Process	Enrichment z-score 0-10%	10-50%	50-90%	90-100%
regulation of cellular macromolecule biosynthetic process	-0,565553225	1,496656278	-0,407702215	-0,523400839
hormone transport	-0,55977901	1,49685698	-0,409339831	-0,52773814
negative regulation of biosynthetic process	-0,545871096	1,497142015	-0,411868877	-0,539402042
cellular amino acid metabolic process	-0,573720597	1,494487541	-0,378390231	-0,542376713
RNA biosynthetic process	-0,55529168	1,495098674	-0,384134882	-0,555672112
regulation of cellular biosynthetic process	-0,610582956	1,492355211	-0,365708927	-0,516063328
intracellular signal transduction	-0,610582956	1,492355211	-0,365708927	-0,516063328
regulation of protein metabolic process	-0,541398419	1,49792594	-0,4251922	-0,53133532
anatomical structure formation involved in morphogenesis	-0,535692048	1,49805306	-0,427320967	-0,535040046
peptide transport	-0,541580026	1,498182305	-0,430358999	-0,52624328
cellular component assembly	-0,531606474	1,49845503	-0,435314947	-0,531533609
response to inorganic substance	-0,517920332	1,49847298	-0,437382508	-0,543170141
signaling	-0,539338218	1,498925011	-0,448691224	-0,51089557
organ morphogenesis	-0,516123195	1,499617994	-0,467959702	-0,515535097
cellular macromolecule metabolic process	-0,520553924	1,499361288	-0,458521532	-0,520285832
nucleic acid metabolic process	-0,518307384	1,499488102	-0,462885794	-0,518294925
purine ribonucleoside triphosphate biosynthetic process	-0,507145788	1,499831969	-0,479105229	-0,513580952
regulation of biosynthetic process	-0,420667236	1,497678336	-0,535171994	-0,541839105
divalent inorganic cation homeostasis	-0,394853496	1,495951619	-0,552886855	-0,548211269
regulation of localization	-0,470734472	1,499624573	-0,503773184	-0,525116917
regulation of catalytic activity	-0,391542975	1,484802403	-0,695706596	-0,397552833
defense response	-0,372862523	1,492063461	-0,62446493	-0,494736008
small molecule biosynthetic process	-0,424063255	1,492524195	-0,638483753	-0,429977186
regulation of signal transduction	-0,424063255	1,492524195	-0,638483753	-0,429977186
cell communication	-0,454112617	1,497134593	-0,586404435	-0,456617541
negative regulation of cellular macromolecule biosynthetic process	-0,532588205	1,492897716	-0,59528302	-0,365026492
regulation of gene expression	-0,547443582	1,495436852	-0,5595201	-0,388473171
growth	-0,539697859	1,497052936	-0,546847747	-0,410507331
regulation of cellular component organization	-0,696113378	1,472491227	-0,539540287	-0,236837562
genitalia development	-0,65402046	1,469401806	-0,608554061	-0,206827285
tissue morphogenesis	-0,65402046	1,469401806	-0,608554061	-0,206827285
response to corticosteroid stimulus	-0,627925382	1,477979039	-0,598220454	-0,251833203
reproductive process	-0,621641004	1,480570529	-0,591627345	-0,26730218
mitotic cell cycle	-0,719938008	1,426247169	-0,667854406	-0,038454755
cell cycle process	-0,728749989	1,432637791	-0,642411966	-0,061475836
nucleobase-containing biosynthetic process	-0,650662866	1,445001392	-0,691381741	-0,102956786
nucleoside phosphate metabolic process	-0,650662866	1,445001392	-0,691381741	-0,102956786
positive regulation of cellular component organization	-0,273333455	1,477493757	-0,695085537	-0,509074765
signal release	-0,223841165	1,466895022	-0,734493036	-0,508560821
protein transport	-0,285096615	1,482929266	-0,632376611	-0,56545604
ribonucleotide biosynthetic process	0,004021439	1,408293814	-0,804583168	-0,607732084
morphogenesis of an epithelium	-0,22755114	1,458939395	-0,440254958	-0,791133297
negative regulation of cellular process	-0,762558278	1,377477285	-0,714889565	0,099970559
response to steroid hormone stimulus	-0,815084588	1,292818259	-0,763339587	0,285605916
regulation of programmed cell death	-0,791081188	1,328626119	0,213133082	-0,750678013
ribonucleoside triphosphate metabolic process	-0,650833348	1,405217243	0,021074875	-0,77545877
positive regulation of molecular function	-0,596388293	1,474700946	-0,234386254	-0,643926399
protein localization	-0,668154212	1,449683599	-0,120031132	-0,661498254
protein localization to organelle	-0,582224957	1,44524438	-0,115375626	-0,747643797
hydrogen transport	-0,489499587	-0,481249084	-0,528926651	1,499675322
sterol biosynthetic process	-0,499354743	-0,500322549	-0,500322553	1,499999844

GO Biological Process	Enrichment z-score 0-10%	10-50%	50-90%	90-100%
regulation of phosphorus metabolic process	-0,467672048	-0,515969327	-0,515969799	1,499611175
intracellular protein kinase cascade	-0,512074995	-0,468020525	-0,51951709	1,49961261
regulation of organelle organization	-0,521148079	-0,445666587	-0,532077938	1,498892605
oxidation-reduction process	-0,493123274	-0,443687727	-0,561439928	1,498250929
striated muscle contraction	-0,485497929	-0,4519187	-0,56102133	1,498437959
cellular macromolecule catabolic process	-0,554155606	-0,361311883	-0,577477568	1,492945058
response to metal ion	-0,543323032	-0,383358038	-0,568276592	1,494957662
regulation of nucleobase-containing compound metabolic process	-0,475268852	-0,41538986	-0,604655005	1,495313716
cytoskeleton organization	-0,492943233	-0,548405023	-0,4576039	1,498952157
cellular component organization	-0,463626391	-0,568584922	-0,465992012	1,498203324
muscle system process	-0,503051377	-0,569199634	-0,425147082	1,497398093
protein heterooligomerization	-0,540649583	-0,519251555	-0,438651681	1,49855282
taxis	-0,522322596	-0,530189495	-0,446417423	1,498929514
muscle contraction	-0,559213839	-0,526187146	-0,411594881	1,496995866
positive regulation of apoptosis	-0,548670006	-0,550128368	-0,397342877	1,496141251
cellular amine metabolic process	-0,553009644	-0,553324166	-0,389177366	1,495511176
cell differentiation	-0,559964398	-0,449936183	-0,488540453	1,498441033
response to glucocorticoid stimulus	-0,562132246	-0,454080965	-0,482214849	1,498428061
protein polymerization	-0,587509465	-0,434638514	-0,474707108	1,496855087
neuron projection morphogenesis	-0,605831902	-0,482928921	-0,406158199	1,494919022
positive regulation of catalytic activity	-0,572722399	-0,494329883	-0,430405063	1,497457346
small molecule metabolic process	-0,479599403	-0,685904186	-0,317337244	1,482840833
proteolysis involved in cellular protein catabolic process	-0,446357021	-0,651265247	-0,393057134	1,490679401
striated muscle cell differentiation	-0,417370459	-0,54248466	-0,537631129	1,497486248
establishment of protein localization	-0,409221286	-0,551422598	-0,536303043	1,496946927
organic acid transport	-0,310545084	-0,593758032	-0,582771407	1,487074523
nematode larval development	-0,373658159	-0,557480729	-0,563042485	1,494181373
cellular divalent inorganic cation homeostasis	-0,360848426	-0,574156484	-0,557928101	1,492933012
response to stress	-0,351801102	-0,599640945	-0,54016424	1,491606287
purine-containing compound metabolic process	-0,362932639	-0,662423042	-0,462975396	1,488331077
gene expression	-0,32284372	-0,666087631	-0,49626804	1,485199391
epithelium development	-0,619793718	-0,609994945	-0,247535909	1,477324571
RNA metabolic process	-0,614667243	-0,625971341	-0,234301096	1,474939679
proteolysis	-0,614667243	-0,625971341	-0,234301096	1,474939679
leukocyte activation	-0,614667243	-0,625971341	-0,234301096	1,474939679
negative regulation of protein metabolic process	-0,614667243	-0,625971341	-0,234301096	1,474939679
pyridine-containing compound metabolic process	-0,614667243	-0,625971341	-0,234301096	1,474939679
regulation of hydrolase activity	-0,632454602	-0,633395487	-0,203024448	1,468874537
negative regulation of cellular biosynthetic process	-0,626257931	-0,638280814	-0,204650423	1,469189168
positive regulation of programmed cell death	-0,677323084	-0,578397847	-0,214241619	1,46996255
purine ribonucleotide biosynthetic process	-0,642562347	-0,575712677	-0,260796832	1,479071856
hermaphrodite genitalia development	-0,627837732	-0,535225223	-0,324833798	1,487896753
regulation of cell cycle process	-0,627837732	-0,535225223	-0,324833798	1,487896753
regulation of phosphorylation	-0,627837732	-0,535225223	-0,324833798	1,487896753
nucleobase-containing catabolic process	-0,566093724	-0,603290137	-0,318593627	1,487977489
oxidoreduction coenzyme metabolic process	-0,659477664	-0,662538748	-0,130278064	1,452294476
regulation of cellular catabolic process	-0,670855551	-0,647037137	-0,135684137	1,453576825
calcium ion transport	-0,653161357	-0,651481337	-0,153254853	1,457897547
organophosphate metabolic process	-0,663990457	-0,405826247	-0,419598808	1,489415513
oogenesis	-0,741295314	-0,384014242	-0,351088491	1,476398046
nicotinamide nucleotide metabolic process	-0,704276589	-0,417133275	-0,361578722	1,482988586
positive regulation of macromolecule metabolic process	-0,820641192	-0,316558902	-0,320142455	1,457342549
mitochondrial transport	-0,76385153	-0,442238079	-0,261139855	1,467229464

GO Biological Process	Enrichment z-score 0-10%	10-50%	50-90%	90-100%
regulation of nitrogen compound metabolic process	-0,185038967	-0,798144282	-0,468981548	1,452164796
regulation of biological quality	-0,270619249	-0,892318765	-0,271267773	1,434205787
response to wounding	-0,321112891	-0,306655443	-0,827562812	1,455331146
cellular calcium ion homeostasis	-0,216267426	-0,158372124	-1,008081679	1,382721229
actin cytoskeleton organization	-0,044212469	-0,706193431	-0,677835888	1,428241788
cell-cell signaling	-0,059635948	-0,7239537	-0,648716643	1,432306291
lipid biosynthetic process	-0,090912243	-0,666294831	-0,68467086	1,441877934
learning or memory	-0,096038788	-0,666426893	-0,680834669	1,44330035
small molecule catabolic process	-0,095487039	-0,664003412	-0,683638248	1,443128699
cell death	-0,135864078	-0,669553339	-0,648217233	1,453634651
purine-containing compound catabolic process	-0,14044872	-0,619551031	-0,694158136	1,454157887
establishment of protein localization to organelle	-0,04566788	-0,527388186	-0,843865597	1,416921663
cellular macromolecule biosynthetic process	-0,265178096	-0,569631473	-0,644844493	1,479654062
response to drug	-0,242185657	-0,578926846	-0,654590908	1,475703411
negative regulation of phosphate metabolic process	-0,221760956	-0,61103914	-0,639695414	1,47249551
cell activation	-0,217486496	-0,603207651	-0,650801057	1,471495205
negative regulation of nucleobase-containing compound metabolic process	-0,236261604	-0,643448148	-0,595328758	1,47503851
ion transport	-0,238509856	-0,648556664	-0,588223445	1,475289965
cellular component morphogenesis	-0,230542542	-0,50265167	-0,734630518	1,46782473
regulation of macromolecule biosynthetic process	-0,313324336	-0,537746667	-0,635241444	1,486312447
translational elongation	-0,272454212	-0,547456243	-0,66007603	1,479986486
cellular biosynthetic process	-0,280832208	-0,529130227	-0,670457042	1,480419477
establishment of protein localization in mitochondrion	-0,306661181	-0,482520133	-0,692085391	1,481266705
protein complex biogenesis	-0,306661181	-0,482520133	-0,692085391	1,481266705
regulation of body fluid levels	-0,354087747	-0,502763862	-0,633354075	1,490205684
regulation of phosphate metabolic process	-0,336740893	-0,508860332	-0,64259744	1,488198664
myeloid leukocyte activation	-0,336740893	-0,508860332	-0,64259744	1,488198664
cellular nitrogen compound biosynthetic process	-0,853133895	-0,172057029	-0,413970973	1,439161898
regulation of protein modification process	-0,765017247	-0,165442481	-0,52332963	1,453789358
ATP metabolic process	-0,629668486	-0,285102951	-0,568243482	1,483014919
mitochondrion organization	-0,629668486	-0,285102951	-0,568243482	1,483014919
purine nucleoside triphosphate metabolic process	-0,642686908	-0,190694015	-0,632911237	1,46629216
organ development	-0,705249749	-0,013747028	-0,699736154	1,41873293
spindle organization	-0,845204816	0,542355589	-0,834886799	1,137736027
death	-0,850192683	0,529767611	-0,825987892	1,146412963
response to estrogen stimulus	-0,864323662	-0,867478729	0,886583345	0,845219046
male gamete generation	-0,864228851	-0,867607117	0,885133554	0,846702413
positive regulation of cellular protein metabolic process	-0,844791661	-0,886756573	0,886512827	0,845035406
metal ion transport	-0,867976886	-0,863153713	0,905410117	0,825720482
oxoacid metabolic process	-0,856241591	-0,850063386	0,64279061	1,063514367
negative regulation of cellular protein metabolic process	-0,85748049	-0,858782746	0,693147295	1,023115941
neurogenesis	-0,857105444	-0,857633695	0,684642119	1,03009702
cell projection morphogenesis	-0,86253887	-0,863617274	0,762121683	0,964034461
response to chemical stimulus	-0,802946501	-0,786610638	0,308376127	1,281181012
negative regulation of macromolecule biosynthetic process	-0,787712294	-0,790664992	0,284847101	1,293530186
divalent metal ion transport	-0,831085624	-0,778890402	0,354091671	1,255884355
regulation of secretion	-0,772972992	-0,789293294	0,252365294	1,309900992
monovalent inorganic cation transport	-0,770941342	-0,797561854	0,26490422	1,303598976
regulation of transmembrane transport	-0,789637226	-0,77671807	0,260469491	1,305885805
positive regulation of protein metabolic process	-0,786337833	-0,76089207	0,223271475	1,323958428
cognition	-0,821857619	-0,820479745	0,432114037	1,210223327
negative regulation of nitrogen compound metabolic process	-0,833281442	-0,841029421	0,523584769	1,150726093
spermatogenesis	-0,849022586	-0,521764798	-0,045151693	1,415939077

GO Biological Process	0-10%	Enrichment z-score<br>10-50%	50-90%	90-100%
cell development	-0,691300834	-0,712912652	-0,014814087	1,419027573
anatomical structure morphogenesis	-0,697966311	-0,707531221	-0,012978467	1,418475999
pyridine nucleotide metabolic process	-0,719909932	-0,721190828	0,041128648	1,399972112
cellular nitrogen compound metabolic process	-0,721457569	-0,723381311	0,046979302	1,397859578
positive regulation of cell death	-0,708518693	-0,71626025	0,015979033	1,40879991
biological regulation	-0,776543967	-0,690057135	0,083169326	1,383431777
alcohol metabolic process	-0,776543967	-0,690057135	0,083169326	1,383431777
actin filament-based process	-0,749582682	-0,76263844	0,158991623	1,353229499
purine ribonucleotide metabolic process	-0,751585137	-0,74137722	0,125590166	1,367372191
mRNA processing	-0,708678501	-0,799342353	0,153276236	1,354744618
neuron projection development	-0,721407598	-0,772094286	0,127006014	1,36649587
regulation of anatomical structure size	-0,707479599	-0,76533084	0,092527891	1,380282548
regulation of protein localization	-0,063408486	-1,358073293	0,976551978	0,444929802
cellular protein catabolic process	0,50025096	-1,228685516	1,062347792	-0,333913235
generation of neurons	-0,712107389	0,015219329	1,409067657	-0,712179597
generation of a signal involved in cell-cell signaling	-0,712575677	-0,076104387	1,437277208	-0,648597144
ion transmembrane transport	-0,636097535	-0,194929416	1,467197732	-0,636170781
reproduction	-0,641339535	-0,19721319	1,46765731	-0,629104585
cell-cell adhesion	-0,742395565	-0,165841981	1,45634069	-0,548103144
monosaccharide metabolic process	-0,555223137	-0,526760029	1,497262296	-0,41527913
transmembrane transport	-0,558319196	-0,555403742	1,494842537	-0,381119599
purine ribonucleoside triphosphate metabolic process	-0,576175076	-0,565713539	1,491823146	-0,34993453
generation of precursor metabolites and energy	-0,564913528	-0,566709764	1,493019042	-0,36139575
positive regulation of intracellular protein kinase cascade	-0,653686659	-0,504274335	1,486715126	-0,328754132
protein import	-0,676891321	-0,431949047	1,487285308	-0,378444941
nucleoside triphosphate biosynthetic process	-0,531959334	-0,493361767	1,499567779	-0,474246678
modification-dependent macromolecule catabolic process	-0,549592054	-0,476163493	1,499064795	-0,473309248
proton transport	-0,542514359	-0,506255767	1,498916715	-0,450146589
cell division	-0,496066192	-0,500806717	1,499993534	-0,503120626
regulation of RNA metabolic process	-0,496164362	-0,506777133	1,499982651	-0,497041156
cell morphogenesis involved in differentiation	-0,459512295	-0,574991363	1,497847177	-0,463343519
response to nutrient	-0,476721708	-0,544501437	1,499248574	-0,47802543
regulation of protein phosphorylation	-0,564137908	-0,37988827	1,494715541	-0,550689363
calcium ion homeostasis	-0,55455112	-0,389132665	1,495506701	-0,551822917
response to endogenous stimulus	-0,568498755	-0,399904382	1,496126302	-0,527723165
heterocycle catabolic process	-0,553144883	-0,439612289	1,498373689	-0,505616516
transcription, DNA-dependent	-0,545960189	-0,423077594	1,497784826	-0,528747043
regulation of response to stimulus	-0,551256308	-0,423279718	1,497735858	-0,523199831
mitochondrial membrane organization	-0,547013379	-0,431716943	1,498186335	-0,519456013
regulation of apoptosis	-0,446636508	-0,665885772	1,488532307	-0,376010027
modification-dependent protein catabolic process	-0,375958913	-0,608821792	1,493163654	-0,508382949
chemotaxis	-0,35206387	-0,630802605	1,490213957	-0,507347481
negative regulation of transport	-0,626638331	-0,638794719	1,468959704	-0,203526654
positive regulation of metabolic process	-0,646372001	-0,648139923	1,460962701	-0,166450777
positive regulation of cellular process	-0,676359737	-0,668618822	1,444176624	-0,099198065
regulation of nucleotide metabolic process	-0,656680728	-0,656809608	1,455100706	-0,141610371
nervous system development	-0,663944042	-0,663969157	1,45029194	-0,122378741
cell part morphogenesis	-0,552723196	-0,674232278	1,475799613	-0,248844139
positive regulation of signal transduction	-0,60297221	-0,61666381	1,479413965	-0,259777945
hormone secretion	-0,620375329	-0,588266374	1,481438216	-0,272796513
cellular protein complex assembly	-0,576896956	-0,579342513	1,49001537	-0,333775901
regulation of primary metabolic process	-0,60045378	-0,572718272	1,487497428	-0,314325376
purine nucleotide biosynthetic process	-0,740744698	-0,741224131	1,374927738	0,107041091

GO Biological Process	Enrichment z-score 0-10%	10-50%	50-90%	90-100%
negative regulation of molecular function	-0,757046607	-0,757458564	1,351505899	0,162999272
macromolecule modification	-0,751559414	-0,751992809	1,359777189	0,143775034
regulation of macromolecule metabolic process	-0,787925091	-0,738396614	1,341570533	0,184751172
cellular process	-0,68226393	-0,700935744	1,428683395	-0,045483721
ribonucleoside triphosphate biosynthetic process	-0,698192689	-0,716948232	1,413686701	0,00145422
neuron differentiation	-0,711241799	-0,729708442	1,399994029	0,040956212
cellular nitrogen compound catabolic process	-0,568105042	-1,065583901	1,138805517	0,494883426
proteasomal protein catabolic process	-0,778823972	-0,874732094	1,188131842	0,465424224
maintenance of location	-0,822866608	-0,829987447	1,192519993	0,460334062
post-embryonic development	-0,830558828	-0,815600798	1,203899072	0,442260553
protein catabolic process	-0,848784383	-0,858129087	1,061306133	0,645607337
neuron development	-0,846418079	-0,725160339	1,297057023	0,274521395
hexose metabolic process	-0,783238713	-0,783602058	1,305440467	0,261400305
purine nucleoside triphosphate biosynthetic process	-0,837839382	-0,787249232	1,235514351	0,389574263
system development	-0,838066261	-0,791163864	1,229650773	0,399579351
amine metabolic process	-0,785766949	-0,822076623	1,258993127	0,348850445
anatomical structure development	-0,819836751	-0,779954935	1,268868807	0,330922879
response to hormone stimulus	-0,802703075	-0,794021748	1,272931773	0,32379305
protein targeting	1,451049721	-0,649096723	-0,676261666	-0,125691332
response to oxygen levels	1,447680101	-0,622144819	-0,70975577	-0,115779511
protein targeting to membrane	1,447680101	-0,622144819	-0,70975577	-0,115779511
divalent inorganic cation transport	1,447680101	-0,622144819	-0,70975577	-0,115779511
protein phosphorylation	1,434471596	-0,683111698	-0,686629141	-0,064730756
regulation of biological process	1,44386436	-0,728763636	-0,611001295	-0,104099429
synaptic transmission	1,44386436	-0,728763636	-0,611001295	-0,104099429
posttranscriptional regulation of gene expression	1,417437802	-0,608945395	-0,787146711	-0,021345696
regulation of metabolic process	1,476881934	-0,609692558	-0,622123242	-0,245066133
regulation of intracellular protein kinase cascade	1,485016019	-0,588987268	-0,600309581	-0,29571917
tissue development	1,488145209	-0,60201022	-0,566335338	-0,319799652
regulation of cell death	1,481785995	-0,628592291	-0,576950182	-0,276243521
translational initiation	1,481621359	-0,638087171	-0,566452871	-0,277081316
cellular response to stimulus	1,492848689	-0,469937992	-0,628476564	-0,394434133
phospholipid metabolic process	1,492848689	-0,469937992	-0,628476564	-0,394434133
nitrogen compound transport	1,492848689	-0,469937992	-0,628476564	-0,394434133
protein complex subunit organization	1,492848689	-0,469937992	-0,628476564	-0,394434133
regulation of protein polymerization	1,491345597	-0,448359606	-0,645895738	-0,397090253
nitrogen compound metabolic process	1,487416626	-0,499947054	-0,652001405	-0,335468168
female gamete generation	1,48406717	-0,397897827	-0,700151588	-0,386017756
metal ion homeostasis	1,499432299	-0,518584157	-0,51993436	-0,460913782
negative regulation of gene expression	1,494829323	-0,530211576	-0,58007717	-0,384540578
axonogenesis	1,49788465	-0,42528663	-0,525353701	-0,547244319
glucose metabolic process	1,495335409	-0,387002267	-0,554372221	-0,553960921
protein complex assembly	1,495296112	-0,389943196	-0,528893005	-0,576459912
mitotic spindle elongation	1,499726836	-0,472991219	-0,511677794	-0,515057823
regulation of cellular component biogenesis	1,499204525	-0,488223593	-0,466875106	-0,544105826
protein modification process	1,489567075	-0,652108422	-0,367367689	-0,470090964
response to oxidative stress	1,488949325	-0,639255548	-0,343016284	-0,506677493
mRNA metabolic process	1,450002203	-0,714946888	-0,126497159	-0,608558155
smooth muscle contraction	1,476140919	-0,532940112	-0,256927463	-0,686273344
axon guidance	1,317086074	0,237732704	-0,773124758	-0,781694021
regulation of cytoskeleton organization	1,428401893	-0,063052771	-0,791309886	-0,574039236
steroid biosynthetic process	1,428401893	-0,063052771	-0,791309886	-0,574039236
protein localization in mitochondrion	1,192374106	-0,80460166	-0,847979651	0,460207204

GO Biological Process	Enrichment z-score			
	0-10%	10-50%	50-90%	90-100%
lipid metabolic process	1,121690264	-0,807015412	-0,878803623	0,564128771
ubiquitin-dependent protein catabolic process	1,142295819	-0,790680324	-0,885796535	0,53418104
response to DNA damage stimulus	1,28435718	-0,79371053	-0,793083839	0,302437189
positive regulation of organelle organization	1,248220462	-0,657682779	-0,941423492	0,350885808
protein targeting to mitochondrion	1,248220462	-0,657682779	-0,941423492	0,350885808
maintenance of protein location	0,874673254	-0,799843689	-0,929714351	0,854884786
apoptosis	0,873252317	-1,005678657	-0,713938245	0,846364585
ATP biosynthetic process	0,107277583	-0,789679454	-0,69128677	1,373688641
developmental process involved in reproduction	0,107277583	-0,789679454	-0,69128677	1,373688641
cytokinesis	0,202369965	-0,735829854	-0,799879425	1,333339315
cellular metal ion homeostasis	0,262703205	-0,779092486	-0,788388605	1,304777886
cellular developmental process	0,389833817	-0,83558585	-0,789664957	1,23541699
biosynthetic process	0,458688862	-0,805837769	-0,846223035	1,193371943
RNA splicing	0,575743895	-0,872253293	-0,81707795	1,113587349
nucleoside monophosphate metabolic process	0,577885838	-0,843124205	-0,847180244	1,112418611
steroid metabolic process	0,598377719	-0,834119846	-0,861516759	1,097258885
body fluid secretion	0,598377719	-0,834119846	-0,861516759	1,097258885
macromolecule biosynthetic process	0,60075571	-0,830447178	-0,865733426	1,095424894
macromolecule catabolic process	0,60075571	-0,830447178	-0,865733426	1,095424894
purine-containing compound biosynthetic process	0,53838542	-1,08305198	-0,560976001	1,105642562
