# Supplementary material for: Alteration of Protein Levels during Influenza Virus H1N1 Infection in Host Cells: A Proteomic Survey of Host and Virus Reveals Differential Dynamics
Source: PLoS One. 2014 Apr 9;9(4):e94257. doi: 10.1371/journal.pone.0094257 (PMC3981805; doi:10.1371/journal.pone.0094257)
Supplement: Table S3 — Links to enriched GO terms for clusters and lists of genes in these clusters. (PDF) [file pone.0094257.s007.pdf]

Table S3: Links to enriched GO terms for clusters and lists of genes in these clusters.

Cluster 1

Enriched Terms:

Gene to GO BP Conditional test for over-representation GOBPID	Pvalue	OddsRatio	ExpCount	Count	Size	Term
**GO:0043436**	0	2,017	26	43	181	oxoacid metabolic process
**GO:0044248**	0	1,844	35	53	241	cellular catabolic process
**GO:0006399**	0,001	3,693	5	12	32	tRNA metabolic process
**GO:0006520**	0,001	2,591	9	19	66	cellular amino acid metabolic process
**GO:0006418**	0,002	3,827	4	10	26	tRNA aminoacylation for protein translation
**GO:0043038**	0,002	3,827	4	10	26	amino acid activation
**GO:0006166**	0,003	Inf	0	3	3	purine ribonucleoside salvage
**GO:0043086**	0,003	2,445	9	17	60	negative regulation of catalytic activity
**GO:0010033**	0,003	1,695	31	45	220	response to organic substance
**GO:0046686**	0,003	3,209	5	11	32	response to cadmium ion
**GO:0070647**	0,004	2,176	12	21	81	protein modification by small protein conjugation or removal
**GO:0044282**	0,004	1,958	16	26	109	small molecule catabolic process
**GO:0006584**	0,005	12,075	1	4	6	catecholamine metabolic process
**GO:0018958**	0,005	12,075	1	4	6	phenol-containing compound metabolic process
**GO:0034311**	0,005	12,075	1	4	6	diol metabolic process
**GO:0006749**	0,006	5,198	2	6	13	glutathione metabolic process
**GO:0043200**	0,006	5,198	2	6	13	response to amino acid stimulus
**GO:0042176**	0,006	4,253	2	7	17	regulation of protein catabolic process
**GO:0000910**	0,007	2,573	6	13	44	cytokinesis
**GO:0043523**	0,008	3,227	4	9	26	regulation of neuron apoptosis
**GO:0010243**	0,008	2,624	6	12	40	response to organic nitrogen
**GO:0006144**	0,008	6,049	1	5	10	purine base metabolic process
**GO:0010038**	0,008	2,393	7	14	51	response to metal ion
**GO:0009636**	0,008	3,476	3	8	22	response to toxin
**GO:0016055**	0,009	4,545	2	6	14	Wnt receptor signaling pathway
**GO:0006725**	0,009	2,689	5	11	36	cellular aromatic compound metabolic process
**GO:0044106**	0,01	5,432	1	5	11	cellular amine metabolic process

Genes in Cluster:

Entrez Id	Symbol	description
607306	VPS4B	vacuolar protein sorting 4 homolog B (S. cerevisiae)
474394	RAB5B	RAB5B, member RAS oncogene family
483970	TXNL1	thioredoxin-like 1
481459	LOC481459	similar to sequestosome 1

476242	PCMT1	protein-L-isoaspartate (D-aspartate) O-methyltransferase
475043	HRSP12	heat-responsive protein 12
481486	ALDH7A1	aldehyde dehydrogenase 7 family, member A1
474844	CSNK2B	casein kinase 2, beta polypeptide
474847	CLIC1	chloride intracellular channel 1
481716	VARS	valyl-tRNA synthetase
484056	EZR	ezrin
475073	NUDCD1	NudC domain containing 1
484063	ACAT2	acetyl-CoA acetyltransferase 2
475081	EIF3H	eukaryotic translation initiation factor 3, subunit H
474484	LOC474484	similar to NHP2-like protein 1 (High mobility group-like nuclear protein 2 homolog 1) ([U4/U6.U5] tri-snRNP 15.5 kDa protein) (Sperm specific antigen 1) (Fertilization antigen 1) (FA-1)
474487	ACO2	aconitase 2, mitochondrial
482049	NDRG1	N-myc downstream regulated 1
475116	TSTA3	tissue specific transplantation antigen P35B
607245	PUF60	poly-U binding splicing factor 60KDa
475189	COPG2	coatomer protein complex, subunit gamma 2
474894	GLO1	glyoxalase I
607816	UBE2H	ubiquitin-conjugating enzyme E2H
474712	PSIP1	PC4 and SFRS1 interacting protein 1
476318	PSAT1	phosphoserine aminotransferase 1
484161	OSTF1	osteoclast stimulating factor 1
482283	CROT	carnitine O-octanoyltransferase
474912	POLR1C	polymerase (RNA) I polypeptide C, 30kDa
403854	TJP2	tight junction protein 2 (zona occludens 2)
474536	TXNRD1	thioredoxin reductase 1
609920	CDK6	cyclin-dependent kinase 6
476337	LOC476337	similar to D19Bwg1357e protein
474928	CD2AP	CD2-associated protein
474759	TLN1	talin 1
611407	PPAT	phosphoribosyl pyrophosphate amidotransferase
494002	MSH2	mutS homolog 2, colon cancer, nonpolyposis type 1 (E. coli)
474798	LOC474798	similar to Thioredoxin (ATL-derived factor) (ADF) (Surface associated sulphydryl protein) (SASP)
475170	GRSF1	G-rich RNA sequence binding factor 1
474981	PGM3	phosphoglucomutase 3
403709	ME1	malic enzyme 1, NADP(+)-dependent, cytosolic
474802	PTGR1	prostaglandin reductase 1
474609	XPO1	exportin 1 (CRM1 homolog, yeast)
475499	OGDH	oxoglutarate (alpha-ketoglutarate) dehydrogenase (lipoamide)
481396	ACTR2	ARP2 actin-related protein 2 homolog (yeast)
475284	SEPT7	septin 7
475506	NUP205	nucleoporin 205kDa
482401	ANLN	anillin, actin binding protein
606756	ANXA4	annexin A4
403487	MTPN	myotrophin
475293	TES	testis derived transcript (3 LIM domains)
474703	PSMD5	proteasome (prosome, macropain) 26S subunit, non-ATPase, 5
482736	ZYX	zyxin
481945	PREP	prolyl endopeptidase
483817	NAA15	N(alpha)-acetyltransferase 15, NatA auxiliary subunit
613007	SNX3	sorting nexin 3
476506	ALDH1L1	aldehyde dehydrogenase 1 family, member L1
483267	NAMPT	nicotinamide phosphoribosyltransferase
476094	ANXA5	annexin A5

475897	PMPCB	peptidase (mitochondrial processing) beta
476425	SAE1	SUMO1 activating enzyme subunit 1
475896	PSMC2	proteasome (prosome, macropain) 26S subunit, ATPase, 2
607058	LOC607058	similar to SVH protein
476434	EML2	echinoderm microtubule associated protein like 2
607116	ESD	esterase D
475156	LOC475156	similar to peroxiredoxin 1
477993	RSU1	Ras suppressor protein 1
477015	PDCD6IP	programmed cell death 6 interacting protein
474963	SUMO2	SMT3 suppressor of mif two 3 homolog 2 (S. cerevisiae)
475913	NUP85	nucleoporin 85kDa
478006	NUDT5	nudix (nucleoside diphosphate linked moiety X)-type motif 5
476449	PAFAH1B3	platelet-activating factor acetylhydrolase 1b, catalytic subunit 3 (29kDa)
607809	OXSR1	oxidative-stress responsive 1
483322	ACOX1	acyl-CoA oxidase 1, palmitoyl
476947	UCHL3	ubiquitin carboxyl-terminal esterase L3 (ubiquitin thiolesterase)
476131	MCM6	minichromosome maintenance complex component 6
483343	TK1	thymidine kinase 1, soluble
476456	BLVRB	biliverdin reductase B (flavin reductase (NADPH))
476459	PSMC4	proteasome (prosome, macropain) 26S subunit, ATPase, 4
483345	LGALS3BP	lectin, galactoside-binding, soluble, 3 binding protein
483372	PYCR1	pyrroline-5-carboxylate reductase 1
476470	PSMD8	proteasome (prosome, macropain) 26S subunit, non-ATPase, 8
476823	RRM1	ribonucleotide reductase M1
476552	TRNT1	tRNA nucleotidyl transferase, CCA-adding, 1
477297	UFM1	ubiquitin-fold modifier 1
482611	LTA4H	leukotriene A4 hydrolase
611746	CAPNS1	calpain, small subunit 1
608169	FKBP1A	FK506 binding protein 1A, 12kDa
477550	LOC477550	similar to poly(rC) binding protein 2 (predicted)
482897	ASAH1	N-acylsphingosine amidohydrolase (acid ceramidase) 1
476490	UBA2	ubiquitin-like modifier activating enzyme 2
485383	IPO7	importin 7
477901	COPS5	COP9 constitutive photomorphogenic homolog subunit 5 (Arabidopsis)
485842	CHMP4B	chromatin modifying protein 4B
476499	PDCD5	programmed cell death 5
475454	ABCE1	ATP-binding cassette, sub-family E (OABP), member 1
483448	OSBP	oxysterol binding protein
607073	ARF4	ADP-ribosylation factor 4
486077	CTSB	cathepsin B
477610	LIMA1	LIM domain and actin binding 1
477213	CPNE1	copine I
475781	HK2	hexokinase 2
478103	GOT2	glutamic-oxaloacetic transaminase 2, mitochondrial (aspartate aminotransferase 2)
478111	CIAPIN1	cytokine induced apoptosis inhibitor 1
486559	PRKAG1	protein kinase, AMP-activated, gamma 1 non-catalytic subunit
486107	DPYSL2	dihydropyrimidinase-like 2
479165	LOC479165	similar to Dihydrofolate reductase
485866	RPRD1B	regulation of nuclear pre-mRNA domain containing 1B
477374	PPP2R2A	protein phosphatase 2, regulatory subunit B, alpha
483118	SPR	sepiapterin reductase (7,8-dihydrobiopterin:NADP+ oxidoreductase)
478453	SEC31A	SEC31 homolog A (S. cerevisiae)
478455	COPS4	COP9 constitutive photomorphogenic homolog subunit 4 (Arabidopsis)
478259	IVD	isovaleryl-CoA dehydrogenase

483631	ARHGAP1	Rho GTPase activating protein 1
479173	TBCA	tubulin folding cofactor A
477501	PEBP1	phosphatidylethanolamine binding protein 1
478482	PDLIM5	PDZ and LIM domain 5
475998	CARS	cysteinyl-tRNA synthetase
478625	NSUN2	NOP2/Sun domain family, member 2
478148	YARS	tyrosyl-tRNA synthetase
477649	DNM1L	dynamin 1-like
478958	UCHL5	ubiquitin carboxyl-terminal hydrolase L5
477813	GSTO1	glutathione S-transferase omega 1
478424	CSTB	cystatin B (stefin B)
486880	XPNPEP1	X-prolyl aminopeptidase (aminopeptidase P) 1, soluble
403532	TNNT2	troponin T type 2 (cardiac)
478500	PPA2	pyrophosphatase (inorganic) 2
487893	AIMP1	aminoacyl tRNA synthetase complex-interacting multifunctional protein 1
609549	PDCD6	programmed cell death 6
475822	ACP6	acid phosphatase 6, lysophosphatidic
478156	FABP3	fatty acid binding protein 3, muscle and heart (mammary-derived growth inhibitor)
611914	SNRNP40	small nuclear ribonucleoprotein 40kDa (U5)
480242	PRMT5	protein arginine methyltransferase 5
477257	CSE1L	CSE1 chromosome segregation 1-like (yeast)
475191	LOC475191	similar to Glutathione S-transferase P (GST 7-7) (Chain 7) (GST class-pi)
477262	UBE2V1	ubiquitin-conjugating enzyme E2 variant 1
483177	SF3B4	splicing factor 3b, subunit 4, 49kDa
488622	MNDA	myeloid cell nuclear differentiation antigen
606979	DCUN1D1	DCN1, defective in cullin neddylation 1, domain containing 1 (S. cerevisiae)
479203	TSNAX	translin-associated factor X
480256	PSME1	proteasome (prosome, macropain) activator subunit 1 (PA28 alpha)
480846	PIR	pirin (iron-binding nuclear protein)
485942	RAE1	RAE1 RNA export 1 homolog (S. pombe)
480260	IPO4	importin 4
486344	AP1B1	adaptor-related protein complex 1, beta 1 subunit
478250	ACTC1	actin, alpha, cardiac muscle 1
475101	LOC475101	similar to L-lactate dehydrogenase B chain (LDH-B) (LDH heart subunit) (LDH-H)
487349	NUDC	nuclear distribution gene C homolog (A. nidulans)
477845	SEC23IP	SEC23 interacting protein
610113	PEA15	phosphoprotein enriched in astrocytes 15
477538	SF3A1	splicing factor 3a, subunit 1, 120kDa
475846	PIP5K1A	phosphatidylinositol-4-phosphate 5-kinase, type I, alpha
478654	PSMD2	proteasome (prosome, macropain) 26S subunit, non-ATPase, 2
488395	GORASP2	golgi reassembly stacking protein 2, 55kDa
479050	IQGAP1	IQ motif containing GTPase activating protein 1
478595	SNX4	sorting nexin 4
480281	SCFD1	sec1 family domain containing 1
480478	PSMC5	proteasome (prosome, macropain) 26S subunit, ATPase, 5
477858	OAT	ornithine aminotransferase
478656	POLR2H	polymerase (RNA) II (DNA directed) polypeptide H
477430	PPP1R7	protein phosphatase 1, regulatory (inhibitor) subunit 7
478799	LOC478799	similar to histone aminotransferase 1 (predicted)
488997	CDK1	cyclin-dependent kinase 1
484781	MAP4	microtubule-associated protein 4
480858	PDHA1	pyruvate dehydrogenase (lipoamide) alpha 1
612814	PAK2	p21 protein (Cdc42/Rac)-activated kinase 2
487373	GALE	UDP-galactose-4-epimerase

478803	OLA1	Obg-like ATPase 1
487374	LYPLA2	lysophospholipase II
490649	CFL2	cofilin 2 (muscle)
480828	ECHS1	enoyl CoA hydratase, short chain, 1, mitochondrial
482601	LOC482601	similar to malate dehydrogenase 2, NAD (mitochondrial)
403979	HSPB1	heat shock 27kDa protein 1
478889	IDH1	isocitrate dehydrogenase 1 (NADP+), soluble
479227	HNRNPH3	heterogeneous nuclear ribonucleoprotein H3 (2H9)
483743	DPF2	D4, zinc and double PHD fingers family 2
479233	VPS26A	vacuolar protein sorting 26 homolog A (S. pombe)
607589	DDT	D-dopachrome tautomerase
479238	PPA1	pyrophosphatase (inorganic) 1
477558	LOC477558	similar to Glutathione S-transferase theta 2 (GST class-theta 2)
479066	IDH3A	isocitrate dehydrogenase 3 (NAD+) alpha
477563	RANBP1	RAN binding protein 1
479444	PPP2R1B	protein phosphatase 2, regulatory subunit A, beta
480772	UPP1	uridine phosphorylase 1
609526	DUT	deoxyuridine triphosphatase
488784	GRPEL1	GrpE-like 1, mitochondrial (E. coli)
479240	PSAP	prosaposin
486715	LPCAT3	lysophosphatidylcholine acyltransferase 3
476032	PRDX5	peroxiredoxin 5
480069	PRDX6	peroxiredoxin 6
486718	USP5	ubiquitin specific peptidase 5 (isopeptidase T)
478907	ARPC2	actin related protein 2/3 complex, subunit 2, 34kDa
490953	VAT1	vesicle amine transport protein 1 homolog (T. californica)
479737	MCM7	minichromosome maintenance complex component 7
479246	ANXA7	annexin A7
478686	PRKCI	protein kinase C, iota
480325	GNPNAT1	glucosamine-phosphate N-acetyltransferase 1
478295	COPS2	COP9 constitutive photomorphogenic homolog subunit 2 (Arabidopsis)
612835	SEC24C	SEC24 family, member C (S. cerevisiae)
490342	METTL13	methyltransferase like 13
479744	ARPC1B	actin related protein 2/3 complex, subunit 1B, 41kDa
479081	LAP3	leucine aminopeptidase 3
610090	PGLS	6-phosphogluconolactonase
477572	UBE2L3	ubiquitin-conjugating enzyme E2L 3
480084	TIPRL	TIP41, TOR signaling pathway regulator-like (S. cerevisiae)
479253	ADK	adenosine kinase
486736	LOC486736	similar to chromosome 12 open reading frame 5
609457	RBM3	RNA binding motif (RNP1, RRM) protein 3
403955	RAC1	ras-related C3 botulinum toxin substrate 1 (rho family, small GTP binding protein Rac1)
487412	RCC2	regulator of chromosome condensation 2
607466	PSMB6	proteasome (prosome, macropain) subunit, beta type, 6
479107	UGDH	UDP-glucose 6-dehydrogenase
480525	TOP2A	topoisomerase (DNA) II alpha 170kDa
479270	ANXA8	annexin A8
476066	CPSF7	cleavage and polyadenylation specific factor 7, 59kDa
611923	SRP9	signal recognition particle 9kDa
607254	LOC607254	similar to EF hand domain containing 2
480364	ARG2	arginase, type II
475298	NAA38	N(alpha)-acetyltransferase 38, NatC auxiliary subunit
491924	MSN	moesin
480538	NPEPPS	aminopeptidase puromycin sensitive

612089	HDGF	hepatoma-derived growth factor
610314	RBM25	RNA binding motif protein 25
490779	ALDH6A1	aldehyde dehydrogenase 6 family, member A1
480129	FDPS	farnesyl diphosphate synthase
609803	RARS	arginyl-tRNA synthetase
476695	FARSA	phenylalanyl-tRNA synthetase, alpha subunit
403892	SULT1A1	sulfotransferase family, cytosolic, 1A, phenol-preferring, member 1
480955	OGT	O-linked N-acetylglucosamine (GlcNAc) transferase (UDP-N-acetylglucosamine:polypeptide-N-acetylglucosaminyl transferase)
491969	LOC491969	similar to CG7519-PA
480135	UBAP2L	ubiquitin associated protein 2-like
480566	SCPEP1	serine carboxypeptidase 1
478355	KIF23	kinesin family member 23
612322	LOC612322	similar to S100 calcium binding protein A14
403787	S100A4	S100 calcium binding protein A4
492015	CSTF2	cleavage stimulation factor, 3' pre-RNA, subunit 2, 64kDa
480143	S100A6	S100 calcium binding protein A6
490465	KATNAL2	katanin p60 subunit A-like 2
403713	ATOX1	ATX1 antioxidant protein 1 homolog (yeast)
611273	MRPL4	mitochondrial ribosomal protein L4
480435	WARS	tryptophanyl-tRNA synthetase
484963	PIN1	peptidylprolyl cis/trans isomerase, NIMA-interacting 1
490508	PSMA8	proteasome (prosome, macropain) subunit, alpha type, 8
489536	SHMT1	serine hydroxymethyltransferase 1 (soluble)
489547	MAP2K3	mitogen-activated protein kinase kinase 3
479545	PGM1	phosphoglucomutase 1
492128	UTP14A	UTP14, U3 small nucleolar ribonucleoprotein, homolog A (yeast)
608647	NAPG	N-ethylmaleimide-sensitive factor attachment protein, gamma
479548	DOCK7	dedicator of cytokinesis 7
479370	TARS	threonyl-tRNA synthetase
442945	HPRT1	hypoxanthine phosphoribosyltransferase 1
479854	USP7	ubiquitin specific peptidase 7 (herpes virus-associated)
485039	SH3GL1	SH3-domain GRB2-like 1
480640	GLOD4	glyoxalase domain containing 4
490021	PPL	periplakin
610939	LOC610939	similar to LSM5 homolog, U6 small nuclear RNA associated
490032	TRAP1	TNF receptor-associated protein 1
491221	TAX1BP3	Tax1 (human T-cell leukemia virus type I) binding protein 3
485067	SGTA	small glutamine-rich tetratricopeptide repeat (TPR)-containing, alpha
489622	LRRC47	leucine rich repeat containing 47
481084	FLNA	filamin A, alpha
479589	ACOT7	acyl-CoA thioesterase 7
481088	G6PD	glucose-6-phosphate dehydrogenase
479595	PARK7	parkinson protein 7
490124	SARS	seryl-tRNA synthetase
479615	APRT	adenine phosphoribosyltransferase
489680	LOC489680	similar to coactosin-like 1
609752	HSBP1	heat shock factor binding protein 1
479937	CNN3	calponin 3, acidic
480712	AK1	adenylate kinase 1
479654	GLG1	golgi glycoprotein 1
609251	LOC609251	similar to niban protein
479656	AARS	alanyl-tRNA synthetase
479658	DDX19A	DEAD (Asp-Glu-Ala-As) box polypeptide 19A
610935	NQO1	NAD(P)H dehydrogenase, quinone 1

480741	NDUFA8	NADH dehydrogenase (ubiquinone) 1 alpha subcomplex, 8, 19kDa
479681	NUTF2	nuclear transport factor 2
490207	ACADM	acyl-CoA dehydrogenase, C-4 to C-12 straight chain
479693	LOC479693	similar to Dynein light intermediate chain 2, cytosolic (LIC53/55) (LIC-2)
479991	CTH	cystathionase (cystathionine gamma-lyase)
403954	RHOA	ras homolog gene family, member A
403686	HMGN2	non-histone chromosomal protein HMG-17
478123	NUDT21	nudix (nucleoside diphosphate linked moiety X)-type motif 21
479731	SRRT	serrate RNA effector molecule homolog (Arabidopsis)
610164	CBR1	carbonyl reductase 1
474939	GSTA4	glutathione S-transferase A4
476177	SERPINB5	serpin peptidase inhibitor, clade B (ovalbumin), member 5
483402	RNH1	ribonuclease/angiogenin inhibitor 1
476022	CFL1	cofilin 1 (non-muscle)

Cluster 2

Enriched Terms:

Gene to GO BP Conditional test for over-representation GOBPID	Pvalue	OddsRatio	ExpCount	Count	Size	Term
**GO:0031397**	0	10,836	6	25	37	negative regulation of protein ubiquitination
**GO:0051352**	0	11,318	6	24	35	negative regulation of ligase activity
**GO:0051436**	0	11,318	6	24	35	negative regulation of ubiquitin-protein ligase activity involved in mitotic cell cycle
**GO:0051437**	0	9,996	7	25	38	positive regulation of ubiquitin-protein ligase activity involved in mitotic cell cycle
**GO:0051351**	0	9,029	7	26	41	positive regulation of ligase activity
**GO:0051438**	0	8,459	7	26	42	regulation of ubiquitin-protein ligase activity
**GO:0032446**	0	5,462	12	35	69	protein modification by small protein conjugation
**GO:0031145**	0	9,564	6	24	37	anaphase-promoting complex-dependent proteasomal ubiquitin-dependent protein catabolic process
**GO:0031398**	0	7,956	7	26	43	positive regulation of protein ubiquitination
**GO:0006511**	0	4,923	13	37	77	ubiquitin-dependent protein catabolic process
**GO:0044257**	0	4,6	14	38	82	cellular protein catabolic process
**GO:0043632**	0	4,682	14	37	79	modification-dependent macromolecule catabolic process
**GO:0048523**	0	2,389	56	95	327	negative regulation of cellular process
**GO:0010498**	0	5,404	10	29	57	proteasomal protein catabolic process
**GO:0032270**	0	4,221	13	33	74	positive regulation of cellular protein metabolic process
**GO:0044092**	0	4,184	12	32	72	negative regulation of molecular function
**GO:0048522**	0	2,185	58	94	341	positive regulation of cellular process
**GO:0031399**	0	3,363	18	40	103	regulation of protein modification process
**GO:0032269**	0	4,146	12	31	70	negative regulation of cellular protein metabolic process
**GO:0009057**	0	3,08	21	45	123	macromolecule catabolic process
**GO:0044093**	0	3	21	44	122	positive regulation of molecular function
**GO:0050790**	0	2,636	25	48	145	regulation of catalytic activity
**GO:0051246**	0	2,484	28	52	164	regulation of protein metabolic process
**GO:0006508**	0	2,525	26	48	149	proteolysis
**GO:0022402**	0	2,585	23	44	138	cell cycle process
**GO:0031323**	0	1,883	65	95	378	regulation of cellular metabolic process
**GO:0010604**	0	2,482	23	43	134	positive regulation of macromolecule metabolic process
**GO:0043412**	0	2	47	73	272	macromolecule modification
**GO:0009056**	0	5,86	4	14	27	catabolic process
**GO:0000278**	0	3,223	11	25	68	mitotic cell cycle

**GO:0010605**	0	2,158	26	45	154	negative regulation of macromolecule metabolic process
**GO:0050789**	0	1,666	126	155	789	regulation of biological process
**GO:0012501**	0	1,945	36	57	212	programmed cell death
**GO:0019320**	0	4,378	5	14	30	hexose catabolic process
**GO:0032271**	0	4,378	5	14	30	regulation of protein polymerization
**GO:0006606**	0	3,648	7	16	38	protein import into nucleus
**GO:0044087**	0	3,058	9	20	53	regulation of cellular component biogenesis
**GO:0046164**	0	4,118	5	14	31	alcohol catabolic process
**GO:0007015**	0	2,948	10	21	57	actin filament organization
**GO:0033365**	0	2,59	13	25	74	protein localization to organelle
**GO:0007346**	0	3,886	5	14	32	regulation of mitotic cell cycle
**GO:0006096**	0	4,971	4	11	22	glycolysis
**GO:0016265**	0	1,814	38	57	222	death
**GO:0071822**	0,001	1,86	32	49	186	protein complex subunit organization
**GO:0044275**	0,001	3,409	6	15	37	cellular carbohydrate catabolic process
**GO:0030216**	0,001	8,628	2	7	11	keratinocyte differentiation
**GO:0006458**	0,001	6,586	2	8	14	'de novo' protein folding
**GO:0051086**	0,001	Inf	1	4	4	chaperone mediated protein folding independent of cofactor
**GO:0070271**	0,001	1,813	30	45	173	protein complex biogenesis
**GO:0072521**	0,001	2,17	15	27	90	purine-containing compound metabolic process
**GO:0006413**	0,002	3,233	6	13	33	translational initiation
**GO:0044248**	0,002	1,649	41	58	241	cellular catabolic process
**GO:0009124**	0,002	3,636	4	11	26	nucleoside monophosphate biosynthetic process
**GO:0009117**	0,002	1,96	20	32	115	nucleotide metabolic process
**GO:0051533**	0,002	12,264	1	5	7	positive regulation of NFAT protein import into nucleus
**GO:0048610**	0,002	2,054	17	28	97	cellular process involved in reproduction
**GO:0016044**	0,002	1,666	38	54	222	cellular membrane organization
**GO:0008064**	0,002	3,31	5	12	30	regulation of actin polymerization or depolymerization
**GO:0000279**	0,002	1,813	26	39	149	M phase
**GO:0006974**	0,003	2,1	15	25	85	response to DNA damage stimulus
**GO:0044267**	0,003	1,529	61	79	385	cellular protein metabolic process
**GO:0016192**	0,003	1,604	43	59	250	vesicle-mediated transport
**GO:0042981**	0,003	1,723	30	44	175	regulation of apoptosis
**GO:0001821**	0,004	19,574	1	4	5	histamine secretion
**GO:0002349**	0,004	19,574	1	4	5	histamine production involved in inflammatory response
**GO:0002553**	0,004	19,574	1	4	5	histamine secretion by mast cell
**GO:0008633**	0,004	19,574	1	4	5	activation of pro-apoptotic gene products
**GO:0035094**	0,004	19,574	1	4	5	response to nicotine
**GO:0035308**	0,004	19,574	1	4	5	negative regulation of protein dephosphorylation
**GO:0046822**	0,004	3,533	4	10	24	regulation of nucleocytoplasmic transport
**GO:0006164**	0,004	2,367	10	18	56	purine nucleotide biosynthetic process
**GO:0010941**	0,004	1,684	31	45	182	regulation of cell death
**GO:0034621**	0,004	1,684	31	45	182	cellular macromolecular complex subunit organization

**GO:0007010**	0,004	1,663	33	47	197	cytoskeleton organization
**GO:0033043**	0,004	2,101	13	23	78	regulation of organelle organization
**GO:0034654**	0,004	2,122	13	22	74	nucleobase-containing biosynthetic process
**GO:0046686**	0,004	2,975	5	12	32	response to cadmium ion
**GO:0009161**	0,005	4,921	2	7	14	ribonucleoside monophosphate metabolic process
**GO:0031109**	0,005	4,921	2	7	14	microtubule polymerization or depolymerization
**GO:0051494**	0,005	5,985	2	6	11	negative regulation of cytoskeleton organization
**GO:0006183**	0,005	Inf	1	3	3	GTP biosynthetic process
**GO:0009220**	0,005	Inf	1	3	3	pyrimidine ribonucleotide biosynthetic process
**GO:0045138**	0,005	Inf	1	3	3	tail tip morphogenesis
**GO:0048566**	0,005	Inf	1	3	3	embryonic digestive tract development
**GO:0021761**	0,005	8,171	1	5	8	limbic system development
**GO:0007052**	0,005	2,009	14	24	84	mitotic spindle organization
**GO:0043624**	0,005	3,295	4	10	25	cellular protein complex disassembly
**GO:0030041**	0,006	4,003	3	8	18	actin filament polymerization
**GO:0010035**	0,006	1,799	21	32	122	response to inorganic substance
**GO:0051234**	0,006	1,395	100	120	585	establishment of localization
**GO:0009119**	0,006	3,942	3	8	18	ribonucleoside metabolic process
**GO:0031333**	0,006	3,942	3	8	18	negative regulation of protein complex assembly
**GO:0018130**	0,006	1,975	15	24	85	heterocycle biosynthetic process
**GO:0009150**	0,007	2,156	11	19	63	purine ribonucleotide metabolic process
**GO:0006006**	0,007	2,342	9	16	50	glucose metabolic process
**GO:0008360**	0,007	3,415	4	9	22	regulation of cell shape
**GO:0009314**	0,007	2,405	8	15	46	response to radiation
**GO:0007030**	0,007	4,303	3	7	15	Golgi organization
**GO:0042990**	0,007	4,303	3	7	15	regulation of transcription factor import into nucleus
**GO:0051650**	0,007	4,303	3	7	15	establishment of vesicle localization
**GO:0032984**	0,008	3,087	4	10	26	macromolecular complex disassembly
**GO:0043627**	0,008	2,863	5	11	30	response to estrogen stimulus
**GO:0043623**	0,008	1,989	13	22	78	cellular protein complex assembly
**GO:0007017**	0,008	1,675	26	37	149	microtubule-based process
**GO:0009260**	0,009	2,419	7	14	43	ribonucleotide biosynthetic process
**GO:0051707**	0,009	1,966	13	22	78	response to other organism
**GO:0009168**	0,009	4,909	2	6	12	purine ribonucleoside monophosphate biosynthetic process
**GO:0030837**	0,009	4,909	2	6	12	negative regulation of actin filament polymerization
**GO:0080090**	0,009	1,596	32	44	201	regulation of primary metabolic process
**GO:0048513**	0,009	1,393	81	98	470	organ development
**GO:0050793**	0,009	1,672	25	36	145	regulation of developmental process
**GO:0009408**	0,009	2,395	7	14	43	response to heat
**GO:0006000**	0,01	9,781	1	4	6	fructose metabolic process
**GO:0007021**	0,01	9,781	1	4	6	tubulin complex assembly
**GO:0030435**	0,01	9,781	1	4	6	sporulation resulting in formation of a cellular spore
**GO:0051220**	0,01	9,781	1	4	6	cytoplasmic sequestering of protein
**GO:0032535**	0,01	2,477	7	13	39	regulation of cellular component size
**GO:0006733**	0,01	3,169	4	9	23	oxidoreduction coenzyme metabolic process

**GO:0046496**	0,01	3,169	4	9	23	nicotinamide nucleotide metabolic process
**GO:0072524**	0,01	3,169	4	9	23	pyridine-containing compound metabolic process
**GO:0002275**	0,01	6,124	2	5	9	myeloid cell activation involved in immune response
**GO:0002444**	0,01	6,124	2	5	9	myeloid leukocyte mediated immunity
**GO:0043299**	0,01	6,124	2	5	9	leukocyte degranulation
**GO:0048205**	0,01	6,124	2	5	9	COPI coating of Golgi vesicle

Genes in cluster:

Entrez Id	Symbol	description
**474393**	DGKA	diacylglycerol kinase, alpha 80kDa
**612760**	PTGES3	prostaglandin E synthase 3 (cytosolic)
**476200**	RNMT	RNA (guanine-7-) methyltransferase
**474414**	MARS	methionyl-tRNA synthetase
**474415**	DCTN2	dynactin 2 (p50)
**474437**	CAND1	cullin-associated and neddylation-dissociated 1
**474441**	CPSF6	cleavage and polyadenylation specific factor 6, 68kDa
**474445**	CCT2	chaperonin containing TCP1, subunit 2 (beta)
**474839**	DDX39B	DEAD (Asp-Glu-Ala-Asp) box polypeptide 39B
**474840**	ATP6V1G2	ATPase, H+ transporting, lysosomal 13kDa, V1 subunit G2
**474657**	SNX2	sorting nexin 2
**474842**	BAG6	BCL2-associated athanogene 6
**475864**	YWHAZ	tyrosine 3-monooxygenase/tryptophan 5-monooxygenase activation protein, zeta polypeptide
**403612**	HSP70	heat shock protein 70
**475070**	EIF3E	eukaryotic translation initiation factor 3, subunit E
**484064**	TCP1	t-complex 1
**608479**	LOC608479	similar to Glyceraldehyde-3-phosphate dehydrogenase (GAPDH)
**474667**	HINT1	histidine triad nucleotide binding protein 1
**474474**	TTLL12	tubulin tyrosine ligase-like family, member 12
**474680**	HSPA4	heat shock 70kDa protein 4
**474682**	SKP1	S-phase kinase-associated protein 1
**403608**	PPP2CA	protein phosphatase 2, catalytic subunit, alpha isozyme
**474685**	DDX46	DEAD (Asp-Glu-Ala-Asp) box polypeptide 46
**483608**	LOC483608	similar to heat shock 70kD protein binding protein
**474499**	ADSL	adenylosuccinate lyase
**442946**	HMGA1	high mobility group AT-hook 1
**475115**	EEF1D	eukaryotic translation elongation factor 1 delta (guanine nucleotide exchange protein)
**476309**	HNRNPK	heterogeneous nuclear ribonucleoprotein K
**476312**	UBQLN1	ubiquilin 1
**474513**	LOC474513	similar to eukaryotic translation initiation factor 3 subunit 6 interacting protein
**482263**	TNPO3	transportin 3
**608665**	EIF3D	eukaryotic translation initiation factor 3, subunit D
**475132**	PSMA4	proteasome (prosome, macropain) subunit, alpha type, 4
**476322**	ANXA1	annexin A1
**476323**	ALDH1A1	aldehyde dehydrogenase 1 family, member A1
**475220**	SRI	sorcin
**481590**	VCP	valosin containing protein
**474919**	HSP90AB1	heat shock protein 90kDa alpha (cytosolic), class B member 1
**475240**	ASNS	asparagine synthetase (glutamine-hydrolyzing)
**475150**	PAICS	phosphoribosylaminoimidazole carboxylase, phosphoribosylaminoimidazole succinocarboxamide synthetase

**609822**	GCLC	glutamate-cysteine ligase, catalytic subunit
**484221**	UBE2M	ubiquitin-conjugating enzyme E2M
**475250**	BZW2	basic leucine zipper and W2 domains 2
**474584**	CALM2	calmodulin 2 (phosphorylase kinase, delta)
**403506**	EEF1A1	eukaryotic translation elongation factor 1 alpha 1
**474585**	MSH6	mutS homolog 6 (E. coli)
**403743**	SPTBN1	spectrin, beta, non-erythrocytic 1
**476400**	ETFB	electron-transfer-flavoprotein, beta polypeptide
**607412**	DBNL	drebrin-like
**475260**	HNRNPA2B1	heterogeneous nuclear ribonucleoprotein A2/B1
**482015**	CBX3	chromobox homolog 3
**475317**	CAP1	CAP, adenylate cyclase-associated protein 1 (yeast)
**482271**	CCT4	chaperonin containing TCP1, subunit 4 (delta)
**475268**	GARS	glycyl-tRNA synthetase
**474614**	MDH1	malate dehydrogenase 1, NAD (soluble)
**475326**	SF3A3	splicing factor 3a, subunit 3, 60kDa
**478521**	LOC478521	similar to 14-3-3 protein theta (14-3-3 protein tau)
**482476**	THRAP3	thyroid hormone receptor associated protein 3
**484370**	NUP62	nucleoporin 62kDa
**475870**	PSMA2	proteasome (prosome, macropain) subunit, alpha type, 2
**475338**	PSMB2	proteasome (prosome, macropain) subunit, beta type, 2
**475346**	VPS35	vacuolar protein sorting 35 homolog (S. cerevisiae)
**476418**	RUVBL2	RuvB-like 2 (E. coli)
**475035**	HDAC2	histone deacetylase 2
**475529**	LUC7L2	LUC7-like 2 (S. cerevisiae)
**475040**	PSMB1	proteasome (prosome, macropain) subunit, beta type, 1
**476512**	RUVBL1	RuvB-like 1 (E. coli)
**476517**	COPG	coatomer protein complex, subunit gamma
**476909**	KPNA3	karyopherin alpha 3 (importin alpha 4)
**403936**	VASP	vasodilator-stimulated phosphoprotein
**475373**	NASP	nuclear autoantigenic sperm protein (histone-binding)
**477991**	VIM	vimentin
**476924**	TPT1	tumor protein, translationally-controlled 1
**483299**	SLC9A3R1	solute carrier family 9 (sodium/hydrogen exchanger), member 3 regulator 1
**477999**	SEPHS1	selenophosphate synthetase 1
**483312**	GRB2	growth factor receptor-bound protein 2
**477951**	LOC477951	similar to suppressor of G2 allele of SKP1
**476115**	DBI	diazepam binding inhibitor (GABA receptor modulator, acyl-CoA binding protein)
**475703**	PPM1G	protein phosphatase, Mg2+/Mn2+ dependent, 1G
**403702**	ACTR3	ARP3 actin-related protein 3 homolog (yeast)
**476454**	SNRPA	small nuclear ribonucleoprotein polypeptide A
**476132**	DARS	aspartyl-tRNA synthetase
**483339**	SEPT9	septin 9
**403818**	GDI2	GDP dissociation inhibitor 2
**475391**	EBNA1BP2	EBNA1 binding protein 2
**482558**	APEX1	APEX nuclease (multifunctional DNA repair enzyme) 1
**475393**	PNP	purine nucleoside phosphorylase
**478019**	PFKP	phosphofructokinase, platelet
**476462**	SUPT5H	suppressor of Ty 5 homolog (S. cerevisiae)
**403809**	RRBP1	ribosome binding protein 1 homolog 180kDa (dog)
**485528**	IPO5	importin 5
**475715**	DPY30	dpy-30 homolog (C. elegans)
**403580**	ACTB	actin, beta
**484526**	ACTN4	actinin, alpha 4

**476468**	EIF3K	eukaryotic translation initiation factor 3, subunit K
**475924**	ARHGDIA	Rho GDP dissociation inhibitor (GDI) alpha
**475926**	DCXR	dicarbonyl/L-xylulose reductase
**483378**	FASN	fatty acid synthase
**477166**	PCNA	proliferating cell nuclear antigen
**476784**	LOC476784	similar to ubiquitin-conjugating enzyme E2N
**475934**	PSMD13	proteasome (prosome, macropain) 26S subunit, non-ATPase, 13
**483048**	EML4	echinoderm microtubule associated protein like 4
**607013**	LOC607013	similar to Coatomer zeta-1 subunit (Zeta-1 coat protein) (Zeta-1 COP)
**477322**	HSPH1	heat shock 105kDa/110kDa protein 1
**475937**	TALDO1	transaldolase 1
**477177**	IDH3B	isocitrate dehydrogenase 3 (NAD+) beta
**442976**	RAN	RAN, member RAS oncogene family
**483413**	API5	apoptosis inhibitor 5
**607950**	SKIV2L2	superkiller viralicidic activity 2-like 2 (S. cerevisiae)
**608575**	COMMD9	COMM domain containing 9
**484700**	PSMD6	proteasome (prosome, macropain) 26S subunit, non-ATPase, 6
**476840**	LOC476840	similar to eukaryotic translation initiation factor 3, subunit 5 epsilon, 47kDa
**486953**	ATP6V1H	ATPase, H+ transporting, lysosomal 50/57kDa, V1 subunit H
**477600**	EIF4B	eukaryotic translation initiation factor 4B
**486788**	IFIT5	interferon-induced protein with tetratricopeptide repeats 5
**475208**	LOC475208	similar to Microtubule-associated protein RP/EB family member 1 (APC-binding protein EB1) (End-binding protein 1) (EB1)
**475950**	EIF3M	eukaryotic translation initiation factor 3, subunit M
**477768**	IDE	insulin-degrading enzyme
**477449**	RILPL1	Rab interacting lysosomal protein-like 1
**477198**	AHCY	adenosylhomocysteinase
**490977**	KRT17	keratin 17
**484713**	FLNB	filamin B, beta
**476854**	EIF4G2	eukaryotic translation initiation factor 4 gamma, 2
**475767**	USP39	ubiquitin specific peptidase 39
**612329**	LSM6	LSM6 homolog, U6 small nuclear RNA associated (S. cerevisiae)
**475770**	MAT2A	methionine adenosyltransferase II, alpha
**483082**	CAPG	capping protein (actin filament), gelsolin-like
**475968**	TIMM10	translocase of inner mitochondrial membrane 10 homolog (yeast)
**479148**	CAST	calpastatin
**613005**	LOC613005	similar to thymosin, beta 10
**477463**	DIABLO	diablo, IAP-binding mitochondrial protein
**478093**	TNPO1	transportin 1
**477914**	TCEB1	transcription elongation factor B (SIII), polypeptide 1 (15kDa, elongin C)
**476719**	LOC476719	similar to Fructose-bisphosphate aldolase A (Muscle-type aldolase) (Lung cancer antigen NY-LU-1)
**477216**	RBM39	RNA binding motif protein 39
**478429**	G3BP2	GTPase activating protein (SH3 domain) binding protein 2
**476866**	COPB1	coatomer protein complex, subunit beta 1
**478430**	USO1	USO1 vesicle docking protein homolog (yeast)
**477474**	ARPC3	actin related protein 2/3 complex, subunit 3, 21kDa
**476867**	PSMA1	proteasome (prosome, macropain) subunit, alpha type, 1
**477477**	VPS29	vacuolar protein sorting 29 homolog (S. cerevisiae)
**477116**	MBNL1	muscleblind-like (Drosophila)
**476588**	TKT	transketolase
**478399**	CCT8	chaperonin containing TCP1, subunit 8 (theta)
**475481**	PPID	peptidylprolyl isomerase D
**475980**	PSMC3	proteasome (prosome, macropain) 26S subunit, ATPase, 3
**474591**	ARF1	ADP-ribosylation factor 1

**477123**	GMPS	guanine monphosphate synthetase
**478447**	ANXA3	annexin A3
**477786**	PGAM1	phosphoglycerate mutase 1 (brain)
**478119**	NUP93	nucleoporin 93kDa
**478406**	SON	SON DNA binding protein
**487948**	LOC487948	similar to coproporphyrinogen oxidase
**476882**	LDHA	lactate dehydrogenase A
**487740**	GART	phosphoribosylglycinamide formyltransferase, phosphoribosylglycinamide synthetase, phosphoribosylaminoimidazole synthetase
**488192**	SERPINB9	serpin peptidase inhibitor, clade B (ovalbumin), member 9
**486122**	LOC486122	similar to p30 DBC protein
**487954**	TFG	TRK-fused gene
**477237**	YWHAB	tyrosine 3-monooxygenase/tryptophan 5-monooxygenase activation protein, beta polypeptide
**478717**	EEF1E1	eukaryotic translation elongation factor 1 epsilon 1
**486600**	TWF1	twinfilin, actin-binding protein, homolog 1 (Drosophila)
**478765**	PSMD14	proteasome (prosome, macropain) 26S subunit, non-ATPase, 14
**483662**	CTSD	cathepsin D
**609496**	NOLC1	nucleolar and coiled-body phosphoprotein 1
**607890**	LOC607890	similar to D-3-phosphoglycerate dehydrogenase (3-PGDH)
**606873**	CCT5	chaperonin containing TCP1, subunit 5 (epsilon)
**486137**	ATP6V1B2	ATPase, H+ transporting, lysosomal 56/58kDa, V1 subunit B2
**606787**	LOC606787	similar to T-complex protein 1, zeta subunit (TCP-1-zeta) (CCT-zeta) (CCT-zeta-1)
**403791**	ACTR1A	ARP1 actin-related protein 1 homolog A, centractin alpha (yeast)
**483670**	NAP1L4	nucleosome assembly protein 1-like 4
**609233**	LOC609233	similar to dynein, cytoplasmic, light peptide
**478145**	AK2	adenylate kinase 2
**610283**	CTTN	cortactin
**478149**	RBBP4	retinoblastoma binding protein 4
**478957**	TROVE2	TROVE domain family, member 2
**607102**	SNRPA1	small nuclear ribonucleoprotein polypeptide A'
**478422**	U2AF1	U2 small nuclear RNA auxiliary factor 1
**480004**	IPO9	importin 9
**478152**	EIF3I	eukaryotic translation initiation factor 3, subunit I
**487981**	ATP6V1A	ATPase, H+ transporting, lysosomal 70kDa, V1 subunit A
**477403**	CAB39	calcium binding protein 39
**612989**	GLTP	glycolipid transfer protein
**487316**	KHDRBS1	KH domain containing, RNA binding, signal transduction associated 1
**478962**	EPRS	glutamyl-prolyl-tRNA synthetase
**608525**	BPNT1	3'(2'), 5'-bisphosphate nucleotidase 1
**480459**	PRKAR1A	protein kinase, cAMP-dependent, regulatory, type I, alpha (tissue specific extinguisher 1)
**480246**	PSMB5	proteasome (prosome, macropain) subunit, beta type, 5
**480465**	PSMD12	proteasome (prosome, macropain) 26S subunit, non-ATPase, 12
**490611**	PABPN1	poly(A) binding protein, nuclear 1
**486633**	BCAT1	branched chain amino-acid transaminase 1, cytosolic
**476007**	CORO1B	coronin, actin binding protein, 1B
**480469**	KPNA2	karyopherin alpha 2 (RAG cohort 1, importin alpha 1)
**403609**	PPP1CA	protein phosphatase 1, catalytic subunit, alpha isozyme
**479406**	HSPA8	heat shock 70kDa protein 8
**475834**	ANP32E	acidic (leucine-rich) nuclear phosphoprotein 32 family, member E
**487332**	RCC1	regulator of chromosome condensation 1
**610210**	TAGLN2	transgelin 2
**476628**	QARS	glutaminyl-tRNA synthetase
**478865**	BZW1	basic leucine zipper and W2 domains 1
**476630**	IMPDH2	IMP (inosine 5'-monophosphate) dehydrogenase 2
**480258**	PSME2	proteasome (prosome, macropain) activator subunit 2 (PA28 beta)

**607749**	EIF4H	eukaryotic translation initiation factor 4H
**486914**	EIF3A	eukaryotic translation initiation factor 3, subunit A
**484774**	PRKAR2A	protein kinase, cAMP-dependent, regulatory, type II, alpha
**488336**	cOR2W9	cOR2W9 olfactory receptor family 2 subfamily W-like
**487351**	SFN	stratifin
**478593**	UMPS	uridine monophosphate synthetase
**610639**	RBM14	RNA binding motif protein 14
**478787**	SSB	Sjogren syndrome antigen B (autoantigen La)
**404305**	PSMA7	proteasome (prosome, macropain) subunit, alpha type, 7
**479415**	ARCN1	archain 1
**477284**	ADRM1	adhesion regulating molecule 1
**480854**	RBBP7	retinoblastoma binding protein 7
**475848**	PSMB4	proteasome (prosome, macropain) subunit, beta type, 4
**478175**	STMN1	stathmin 1
**480479**	DDX42	DEAD (Asp-Glu-Ala-Asp) box polypeptide 42
**487367**	CLIC4	chloride intracellular channel 4
**475852**	LOC475852	similar to Calgizzarin (S100 calcium-binding protein A11) (S100C protein) (MLN 70)
**478185**	SRRM1	serine/arginine repetitive matrix 1
**487886**	SEPT2	septin 2
**476015**	SF3B2	splicing factor 3b, subunit 2, 145kDa
**476640**	SMARCC1	SWI/SNF related, matrix associated, actin dependent regulator of chromatin, subfamily c, member 1
**477691**	DDX47	DEAD (Asp-Glu-Ala-Asp) box polypeptide 47
**479425**	PAFAH1B2	platelet-activating factor acetylhydrolase 1b, catalytic subunit 2 (30kDa)
**477869**	GLRX3	glutaredoxin 3
**480037**	ARPC5	actin related protein 2/3 complex, subunit 5, 16kDa
**478881**	EEF1B2	eukaryotic translation elongation factor 1 beta 2
**480290**	PSMA6	proteasome (prosome, macropain) subunit, alpha type, 6
**475857**	CAPZA1	capping protein (actin filament) muscle Z-line, alpha 1
**608979**	UCK2	uridine-cytidine kinase 2
**490921**	NSF	N-ethylmaleimide-sensitive factor
**489818**	YWHAG	tyrosine 3-monooxygenase/tryptophan 5-monooxygenase activation protein, gamma polypeptide
**487532**	EIF3J	eukaryotic translation initiation factor 3, subunit J
**484785**	CCDC12	coiled-coil domain containing 12
**488118**	EIF4A2	eukaryotic translation initiation factor 4A2
**480045**	TPR	translocated promoter region (to activated MET oncogene)
**479433**	ZW10	ZW10, kinetochore associated, homolog (Drosophila)
**480048**	PLA2G4A	phospholipase A2, group IVA (cytosolic, calcium-dependent)
**480295**	SEC23A	Sec23 homolog A (S. cerevisiae)
**481417**	PCBP1	poly(rC) binding protein 1
**479441**	CRYAB	crystallin, alpha B
**479068**	WDR61	WD repeat domain 61
**476031**	SF1	splicing factor 1
**488513**	ATIC	5-aminoimidazole-4-carboxamide ribonucleotide formyltransferase/IMP cyclohydrolase
**480062**	CACYBP	calcyclin binding protein
**476033**	TRMT112	tRNA methyltransferase 11-2 homolog (S. cerevisiae)
**477711**	TPI1	triosephosphate isomerase 1
**474837**	STIP1	stress-induced-phosphoprotein 1
**480512**	PSME3	proteasome (prosome, macropain) activator subunit 3 (PA28 gamma; Ki)
**476039**	OTUB1	OTU domain, ubiquitin aldehyde binding 1
**478522**	PSMC6	proteasome (prosome, macropain) 26S subunit, ATPase, 6
**480896**	UBA1	ubiquitin-like modifier activating enzyme 1
**403581**	PPIA	peptidylprolyl isomerase A (cyclophilin A)
**480329**	MAPK1IP1L	mitogen-activated protein kinase 1 interacting protein 1-like
**404021**	LGALS3	lectin, galactoside-binding, soluble, 3

**489861**	PDAP1	PDGFA associated protein 1
**478209**	CAPZB	capping protein (actin filament) muscle Z-line, beta
**480338**	PSMA3	proteasome (prosome, macropain) subunit, alpha type, 3
**478922**	DNPEP	aspartyl aminopeptidase
**477575**	MAPK1	mitogen-activated protein kinase 1
**611070**	WDR1	WD repeat domain 1
**477726**	FKBP4	FK506 binding protein 4, 59kDa
**477584**	ATAD1	ATPase family, AAA domain containing 1
**476055**	UBXN1	UBX domain protein 1
**479259**	ANXA11	annexin A11
**609879**	TPM4	tropomyosin 4
**476570**	LOC476570	similar to Elongation factor 1-gamma (EF-1-gamma) (eEF-1B gamma)
**480352**	MTHFD1	methylenetetrahydrofolate dehydrogenase (NADP+ dependent) 1, methenyltetrahydrofolate cyclohydrolase, formyltetrahydrofolate synthetase
**480921**	GSPT2	G1 to S phase transition 2
**477736**	LOC477736	similar to cat eye syndrome chromosome region, candidate 5 isoform 2 precursor
**478926**	FARSB	phenylalanyl-tRNA synthetase, beta subunit
**477740**	ATP6V1E1	ATPase, H+ transporting, lysosomal 31kDa, V1 subunit E1
**476067**	DDB1	damage-specific DNA binding protein 1, 127kDa
**491018**	PSMD3	proteasome (prosome, macropain) 26S subunit, non-ATPase, 3
**476689**	DDX39A	DEAD (Asp-Glu-Ala-Asp) box polypeptide 39A
**480118**	CAPN2	calpain 2, (m/II) large subunit
**479766**	EIF3B	eukaryotic translation initiation factor 3, subunit B
**611552**	PRPF19	PRP19/PSO4 pre-mRNA processing factor 19 homolog (S. cerevisiae)
**480369**	ACTN1	actinin, alpha 1
**608624**	LASP1	LIM and SH3 protein 1
**480371**	ERH	enhancer of rudimentary homolog (Drosophila)
**474987**	LOC474987	similar to Proteasome subunit beta type 3 (Proteasome theta chain) (Proteasome chain 13) (Proteasome component C10-II)
**479485**	EIF4A1	eukaryotic translation initiation factor 4A1
**491042**	KPNB1	karyopherin (importin) beta 1
**612240**	CTBP1	C-terminal binding protein 1
**478236**	PGD	phosphogluconate dehydrogenase
**476790**	LOC476790	similar to chaperonin containing TCP1, subunit 3 isoform c
**479795**	EIF3C	eukaryotic translation initiation factor 3, subunit C
**484926**	PRDX2	peroxiredoxin 2
**480402**	AHSA1	AHA1, activator of heat shock 90kDa protein ATPase homolog 1 (yeast)
**480964**	PGK1	phosphoglycerate kinase 1
**478347**	MAP2K1	mitogen-activated protein kinase kinase 1
**480559**	NME2	non-metastatic cells 2, protein (NM23B) expressed in
**403534**	ANP32A	acidic (leucine-rich) nuclear phosphoprotein 32 family, member A
**480979**	HNRNPK	heterogeneous nuclear ribonucleoprotein K
**478703**	PSMC1	proteasome (prosome, macropain) 26S subunit, ATPase, 1
**480986**	TIMM8A	translocase of inner mitochondrial membrane 8 homolog A (yeast)
**489167**	LOC489167	similar to CG9590-PA
**403874**	PKM2	pyruvate kinase, muscle
**480151**	LOC480151	similar to CG14299-PA, isoform A
**480989**	HNRNPH2	heterogeneous nuclear ribonucleoprotein H2 (H')
**484955**	CDC37	cell division cycle 37 homolog (S. cerevisiae)
**480595**	AATF	apoptosis antagonizing transcription factor
**612971**	EIF3G	eukaryotic translation initiation factor 3, subunit G
**480437**	LOC480437	similar to dynein, cytoplasmic, heavy polypeptide 1
**491130**	ACACA	acetyl-CoA carboxylase alpha
**491146**	CCT6B	chaperonin containing TCP1, subunit 6B (zeta 2)
**480182**	USP14	ubiquitin specific peptidase 14 (tRNA-guanine transglycosylase)

**480610**	PSMD11	proteasome (prosome, macropain) 26S subunit, non-ATPase, 11
**479528**	COPS3	COP9 constitutive photomorphogenic homolog subunit 3 (Arabidopsis)
**476725**	HNRNPM	heterogeneous nuclear ribonucleoprotein M
**479344**	HMGCS1	3-hydroxy-3-methylglutaryl-CoA synthase 1 (soluble)
**485022**	KHSRP	KH-type splicing regulatory protein
**480622**	ALDOC	aldolase C, fructose-bisphosphate
**479846**	GSPT1	G1 to S phase transition 1
**480631**	NUFIP2	nuclear fragile X mental retardation protein interacting protein 2
**481063**	RBMX	RNA binding motif protein, X-linked
**480645**	YWHAE	tyrosine 3-monooxygenase/tryptophan 5-monooxygenase activation protein, epsilon polypeptide
**476744**	EEF2	eukaryotic translation elongation factor 2
**491201**	LOC491201	similar to CG7338-PA
**609638**	SRRM2	serine/arginine repetitive matrix 2
**479575**	ISG15	ISG15 ubiquitin-like modifier
**479873**	TCEB2	transcription elongation factor B (SIII), polypeptide 2 (18kDa, elongin B)
**612188**	SF3A2	splicing factor 3a, subunit 2, 66kDa
**611128**	HN1L	hematological and neurological expressed 1-like
**403819**	GDI1	GDP dissociation inhibitor 1
**479597**	ENO1	enolase 1, (alpha)
**490123**	PSMA5	proteasome (prosome, macropain) subunit, alpha type, 5
**479625**	USP10	ubiquitin specific peptidase 10
**403484**	SPTAN1	spectrin, alpha, non-erythrocytic 1 (alpha-fodrin)
**479644**	KARS	lysyl-tRNA synthetase
**612856**	ARPC5L	actin related protein 2/3 complex, subunit 5-like
**479671**	PSMD7	proteasome (prosome, macropain) 26S subunit, non-ATPase, 7
**490201**	FUBP1	far upstream element (FUSE) binding protein 1
**476166**	CNDP2	CNDP dipeptidase 2 (metallopeptidase M20 family)
**476767**	NME1	non-metastatic cells 1, protein (NM23A) expressed in
**476540**	ARPC4	actin related protein 2/3 complex, subunit 4, 20kDa
**478680**	KPNA4	karyopherin alpha 4 (importin alpha 3)
**488473**	CASP8	caspase 8, apoptosis-related cysteine peptidase
**477928**	CA2	carbonic anhydrase II

Cluster 3

Enriched Terms:

Gene to GO BP Conditional test for over-representation GOBPID	Pvalue	OddsRatio	ExpCount	Count	Size	Term
**GO:0006414**	0	4,073	5	16	79	translational elongation
**GO:0006270**	0	29,847	0	4	6	DNA-dependent DNA replication initiation
**GO:0000003**	0,001	1,955	28	43	427	reproduction
**GO:0042303**	0,001	4,306	3	9	42	molting cycle
**GO:0018996**	0,001	4,175	3	9	42	molting cycle, collagen and cuticulin-based cuticle
**GO:0010467**	0,001	1,814	36	51	551	gene expression
**GO:0006892**	0,002	5,648	1	6	22	post-Golgi vesicle-mediated transport
**GO:0042274**	0,002	5,648	1	6	22	ribosomal small subunit biogenesis
**GO:0009059**	0,003	1,813	25	38	390	macromolecule biosynthetic process
**GO:0033572**	0,004	Inf	0	2	2	transferrin transport
**GO:0051304**	0,004	Inf	0	2	2	chromosome separation
**GO:0007017**	0,006	2,156	10	18	149	microtubule-based process
**GO:0030855**	0,008	4,093	2	6	28	epithelial cell differentiation
**GO:0022404**	0,008	6,606	1	4	13	molting cycle process
**GO:0033205**	0,008	11,086	0	3	7	cell cycle cytokinesis
**GO:0035017**	0,008	11,086	0	3	7	cuticle pattern formation

Genes in cluster:

Entrez Id	Symbol	description
**474396**	PA2G4	proliferation-associated 2G4, 38kDa
**607631**	NARS	asparaginyl-tRNA synthetase
**474408**	NACA	nascent polypeptide-associated complex alpha subunit
**476206**	RPS12	ribosomal protein S12
**474826**	ABCF1	ATP-binding cassette, sub-family F (GCN20), member 1
**474663**	LMNB1	lamin B1
**474696**	LOC474696	similar to eukaryotic translation termination factor 1 (predicted)
**474508**	DDX17	DEAD (Asp-Glu-Ala-Asp) box polypeptide 17
**482083**	PLEC	plectin
**474519**	TXN2	thioredoxin 2
**474722**	RPS6	ribosomal protein S6
**475199**	ATP6V1F	ATPase, H+ transporting, lysosomal 14kDa, V1 subunit F
**481280**	MYH9	myosin, heavy chain 9, non-muscle
**475204**	SND1	staphylococcal nuclease and tudor domain containing 1
**610519**	MCM5	minichromosome maintenance complex component 5
**474576**	LOC474576	similar to DnaJ homolog subfamily A member 1 (Heat shock 40 kDa protein 4) (DnaJ protein homolog 2) (HSJ-2) (HSDJ)
**474740**	SMU1	smu-1 suppressor of mec-8 and unc-52 homolog (C. elegans)
**481839**	MCM3	minichromosome maintenance complex component 3
**403944**	SRP72	signal recognition particle 72kDa
**474556**	EIF5B	eukaryotic translation initiation factor 5B
**474765**	CLTA	clathrin, light chain A
**476378**	RPL28	ribosomal protein L28

**475321**	MACF1	microtubule-actin crosslinking factor 1
**475649**	RPS7	ribosomal protein S7
**482963**	RRM2	ribonucleotide reductase M2
**476411**	PRMT1	protein arginine methyltransferase 1
**475671**	DDX1	DEAD (Asp-Glu-Ala-Asp) box polypeptide 1
**475682**	LOC475682	similar to splicing factor 3B, 14 kDa subunit
**612058**	GRWD1	glutamate-rich WD repeat containing 1
**484622**	MCM2	minichromosome maintenance complex component 2
**477968**	KIF5B	kinesin family member 5B
**476111**	MKI67IP	MKI67 (FHA domain) interacting nucleolar phosphoprotein
**483009**	CAD	carbamoyl-phosphate synthetase 2, aspartate transcarbamylase, and dihydroorotase
**475540**	LOC475540	similar to testis-specific histone 2b
**403952**	SRP68	signal recognition particle 68kDa
**477029**	RPSA	ribosomal protein SA
**607653**	SEC13	SEC13 homolog (S. cerevisiae)
**476460**	FBL	fibrillarin
**474964**	HNRNPCL1	heterogeneous nuclear ribonucleoprotein C-like 1
**475719**	TTC27	tetratricopeptide repeat domain 27
**475721**	LOC475721	similar to CG5913-PA
**607948**	LOC607948	similar to THO complex subunit 4 (Tho4) (Ally of AML-1 and LEF-1) (Transcriptional coactivator Aly/REF) (bZIP enhancing factor BEF)
**475429**	METAP2	methionyl aminopeptidase 2
**483399**	PKP3	plakophilin 3
**477871**	MCM4	minichromosome maintenance complex component 4
**485806**	SNRPB	small nuclear ribonucleoprotein polypeptides B and B1
**477879**	TCEA1	transcription elongation factor A (SII), 1
**477887**	RPS20	ribosomal protein S20
**477757**	HNRNPF	heterogeneous nuclear ribonucleoprotein F
**477601**	KRT18	keratin 18
**486513**	KRT8	keratin 8
**477602**	KRT7	keratin 7
**609784**	VAMP8	vesicle-associated membrane protein 8 (endobrevin)
**478864**	LOC478864	similar to 60S ribosomal protein L21
**477309**	LOC477309	similar to Transcription factor BTF3 homolog 3
**478254**	LOC478254	similar to CG5913-PA
**477224**	CTNNBL1	catenin, beta like 1
**488448**	GLS	glutaminase
**488207**	DSP	desmoplakin
**487314**	KPNA6	karyopherin alpha 6 (importin alpha 7)
**475816**	SEC22B	SEC22 vesicle trafficking protein homolog B (S. cerevisiae) (gene/pseudogene)
**478504**	PAPSS1	3'-phosphoadenosine 5'-phosphosulfate synthase 1
**480311**	RPL36AL	ribosomal protein L36a-like
**478642**	FXR1	fragile X mental retardation, autosomal homolog 1
**476855**	PRPS2	phosphoribosyl pyrophosphate synthetase 2
**442955**	EPB41	erythrocyte membrane protein band 4.1 (elliptocytosis 1, RH-linked)
**480472**	DDX5	DEAD (Asp-Glu-Ala-Asp) box polypeptide 5
**478165**	ATPIF1	ATPase inhibitory factor 1
**610764**	CAMK2D	calcium/calmodulin-dependent protein kinase II delta
**475838**	GOLPH3L	golgi phosphoprotein 3-like
**477274**	VAPB	VAMP (vesicle-associated membrane protein)-associated protein B and C
**479071**	LOC479071	similar to Probable ATP-dependent RNA helicase DDX6 (DEAD-box protein 6) (ATP-dependent RNA helicase p54) (Oncogene RCK)
**477856**	ACADSB	acyl-CoA dehydrogenase, short/branched chain
**485959**	RPS21	ribosomal protein S21
**486663**	STRAP	serine/threonine kinase receptor associated protein

**477857**	BUB3	budding uninhibited by benzimidazoles 3 homolog (yeast)
**477431**	HDLBP	high density lipoprotein binding protein
**611715**	EIF1AX	eukaryotic translation initiation factor 1A, X-linked
**479230**	CCAR1	cell division cycle and apoptosis regulator 1
**480306**	FKBP3	FK506 binding protein 3, 25kDa
**479237**	SAR1A	SAR1 homolog A (S. cerevisiae)
**480886**	DDX3X	DEAD (Asp-Glu-Ala-Asp) box polypeptide 3, X-linked
**479733**	GNB2	guanine nucleotide binding protein (G protein), beta polypeptide 2
**478831**	ZC3H15	zinc finger CCCH-type containing 15
**476663**	COPE	coatomer protein complex, subunit epsilon
**479969**	LOC479969	similar to Protein C14orf166
**477714**	CHD4	chromodomain helicase DNA binding protein 4
**490966**	PTRF	polymerase I and transcript release factor
**480332**	KTN1	kinectin 1 (kinesin receptor)
**478918**	TUBA4A	tubulin, alpha 4a
**610636**	TUBA3C	tubulin, alpha 3c
**489450**	C1QBP	complement component 1, q subcomponent binding protein
**476053**	HNRNPUL2	heterogeneous nuclear ribonucleoprotein U-like 2
**607852**	ACLY	ATP citrate lyase
**480100**	HNRNPU	heterogeneous nuclear ribonucleoprotein U (scaffold attachment factor A)
**480522**	JUP	junction plakoglobin
**489468**	EIF5A	eukaryotic translation initiation factor 5A
**403682**	RPL19	ribosomal protein L19
**488856**	DHX15	DEAH (Asp-Glu-Ala-His) box polypeptide 15
**403683**	RPL23	ribosomal protein L23
**490754**	SRSF5	serine/arginine-rich splicing factor 5
**403435**	ANXA2	annexin A2
**479282**	NOP16	NOP16 nucleolar protein homolog (yeast)
**480124**	LMNA	lamin A/C
**475722**	LOC475722	similar to nucleophosmin 1
**490449**	RPS27	ribosomal protein S27
**480578**	CLTC	clathrin, heavy chain (Hc)
**484949**	DNM2	dynamin 2
**479322**	G3BP1	GTPase activating protein (SH3 domain) binding protein 1
**403690**	RPS14	ribosomal protein S14
**480440**	TNFAIP2	tumor necrosis factor, alpha-induced protein 2
**480442**	EIF5	eukaryotic translation initiation factor 5
**480193**	MYL12B	myosin, light chain 12B, regulatory
**479535**	SERBP1	SERPINE1 mRNA binding protein 1
**476799**	RPL23A	ribosomal protein L23a
**485028**	SAFB	scaffold attachment factor B
**480213**	TUBB6	tubulin, beta 6
**481079**	NSDHL	NAD(P) dependent steroid dehydrogenase-like
**489591**	CDK11A	cyclin-dependent kinase 11A
**492246**	HCFC1	host cell factor C1 (VP16-accessory protein)
**491231**	TUBB2C	tubulin, beta 2C
**480674**	EDF1	endothelial differentiation-related factor 1
**479949**	RPL5	ribosomal protein L5
**479666**	AP1G1	adaptor-related protein complex 1, gamma 1 subunit
**489733**	NOB1	NIN1/RPN12 binding protein 1 homolog (S. cerevisiae)
**489738**	CIRH1A	cirrhosis, autosomal recessive 1A (cirhin)
**475851**	S100A10	S100 calcium binding protein A10
**480818**	GNB2L1	guanine nucleotide binding protein (G protein), beta polypeptide 2-like 1
**487554**	TMOD3	tropomodulin 3 (ubiquitous)

Cluster 4

Enriched Terms:

Gene to GO BP Conditional test for over-representation GOBPID	Pvalue	OddsRatio	ExpCount	Count	Size	Term
**GO:0071824**	0	5,528	4	15	35	protein-DNA complex subunit organization
**GO:0006334**	0	6,35	4	13	28	nucleosome assembly
**GO:0046942**	0	5,836	3	12	27	carboxylic acid transport
**GO:0006333**	0	4,409	5	15	40	chromatin assembly or disassembly
**GO:0055085**	0	3,545	7	18	57	transmembrane transport
**GO:0006323**	0	3,663	6	15	45	DNA packaging
**GO:0006200**	0	10,051	2	7	12	ATP catabolic process
**GO:0006172**	0	Inf	1	4	4	ADP biosynthetic process
**GO:0009136**	0	Inf	1	4	4	purine nucleoside diphosphate biosynthetic process
**GO:0009188**	0	Inf	1	4	4	ribonucleoside diphosphate biosynthetic process
**GO:0006820**	0	7,374	2	8	16	anion transport
**GO:0022904**	0	5,168	3	10	24	respiratory electron transport chain
**GO:0009206**	0	4,153	4	12	33	purine ribonucleoside triphosphate biosynthetic process
**GO:0015992**	0	5,409	3	9	21	proton transport
**GO:0015711**	0	10,728	1	6	10	organic anion transport
**GO:0051234**	0,001	1,627	73	96	585	establishment of localization
**GO:0006839**	0,001	3,787	4	12	35	mitochondrial transport
**GO:0009142**	0,001	3,787	4	12	35	nucleoside triphosphate biosynthetic process
**GO:0015980**	0,001	3,002	7	16	55	energy derivation by oxidation of organic compounds
**GO:0008203**	0,001	5,751	2	8	18	cholesterol metabolic process
**GO:0034220**	0,001	3,982	4	11	31	ion transmembrane transport
**GO:0009179**	0,001	28,414	1	4	5	purine ribonucleoside diphosphate metabolic process
**GO:0019233**	0,001	28,414	1	4	5	sensory perception of pain
**GO:0033555**	0,001	11,876	1	5	8	multicellular organismal response to stress
**GO:0040008**	0,001	1,861	25	40	203	regulation of growth
**GO:0040010**	0,002	1,98	19	31	148	positive regulation of growth rate
**GO:0009853**	0,002	Inf	0	3	3	photorespiration
**GO:0015800**	0,002	8,902	1	5	9	acidic amino acid transport
**GO:0006119**	0,003	4,416	3	8	21	oxidative phosphorylation
**GO:0051240**	0,003	2,748	6	14	51	positive regulation of multicellular organismal process

**GO:0015986**	0,003	5,01	2	7	17	ATP synthesis coupled proton transport
**GO:0014911**	0,003	14,198	1	4	6	positive regulation of smooth muscle cell migration
**GO:0015837**	0,003	3,806	3	9	26	amine transport
**GO:0030001**	0,004	3,101	5	11	37	metal ion transport
**GO:0030155**	0,004	4,552	2	7	18	regulation of cell adhesion
**GO:0006816**	0,005	3,822	3	8	23	calcium ion transport
**GO:0007568**	0,006	1,961	14	24	114	aging
**GO:0019915**	0,006	4,17	2	7	19	lipid storage
**GO:0006814**	0,006	9,46	1	4	7	sodium ion transport
**GO:0019218**	0,006	9,46	1	4	7	regulation of steroid metabolic process
**GO:0051276**	0,007	1,99	13	22	103	chromosome organization
**GO:0009199**	0,007	2,54	6	13	50	ribonucleoside triphosphate metabolic process
**GO:0016126**	0,007	4,754	2	6	15	sterol biosynthetic process
**GO:0001662**	0,007	21,219	1	3	4	behavioral fear response
**GO:0006971**	0,007	21,219	1	3	4	hypotonic response
**GO:0046513**	0,007	21,219	1	3	4	ceramide biosynthetic process
**GO:0001817**	0,007	5,927	1	5	11	regulation of cytokine production
**GO:0008610**	0,008	2,234	9	16	68	lipid biosynthetic process
**GO:0022610**	0,008	2,234	9	16	68	biological adhesion
**GO:0009144**	0,008	2,471	6	13	51	purine nucleoside triphosphate metabolic process
**GO:0016049**	0,008	2,874	4	10	35	cell growth
**GO:0006812**	0,009	3,752	3	7	21	cation transport

Genes in cluster:

Entrey Id	Symbol	description
**476186**	LMAN1	lectin, mannose-binding, 1
**474397**	ESYT1	extended synaptotagmin-like protein 1
**403669**	ATP5B	ATP synthase, H+ transporting, mitochondrial F1 complex, beta polypeptide
**474630**	HSD17B4	hydroxysteroid (17-beta) dehydrogenase 4
**483987**	STX7	syntaxin 7
**607458**	SHMT2	serine hydroxymethyltransferase 2 (mitochondrial)
**481436**	TMED7	transmembrane emp24 protein transport domain containing 7
**474644**	NHP2	NHP2 ribonucleoprotein homolog (yeast)
**481455**	HNRNPH1	heterogeneous nuclear ribonucleoprotein H1 (H)
**403908**	CANX	calnexin
**608981**	RAP1B	RAP1B, member of RAS oncogene family
**474448**	TSPAN8	tetraspanin 8
**481991**	LOC481991	similar to small nuclear ribonucleoprotein D2
**474475**	TSPO	translocator protein (18kDa)
**474479**	CYB5R3	cytochrome b5 reductase 3
**474681**	VDAC1	voltage-dependent anion channel 1
**403935**	ANXA13	annexin A13
**608021**	SQLE	squalene epoxidase
**481514**	H2AFY	H2A histone family, member Y
**481746**	ITPR3	inositol 1,4,5-trisphosphate receptor, type 3
**474697**	HSPA9	heat shock 70kDa protein 9 (mortalin)

**476303**	GOLM1	golgi membrane protein 1
**610117**	TOMM22	translocase of outer mitochondrial membrane 22 homolog (yeast)
**482252**	PODXL	podocalyxin-like
**403687**	SRSF3	serine/arginine-rich splicing factor 3
**475121**	LOC475121	similar to cytochrome c-1
**609079**	TOMM6	translocase of outer mitochondrial membrane 6 homolog (yeast)
**474526**	PWP1	PWP1 homolog (S. cerevisiae)
**612937**	TMEM33	transmembrane protein 33
**475225**	LOC475225	similar to Cytochrome P450 51A1 (CYPLI) (P450LI) (Sterol 14-alpha demethylase) (Lanosterol 14-alpha demethylase) (LDM) (P450-14DM) (P45014DM)
**476333**	DOCK8	dedicator of cytokinesis 8
**475140**	OCIAD1	OCIA domain containing 1
**474755**	STOML2	stomatin (EPB72)-like 2
**484192**	SPTLC1	serine palmitoyltransferase, long chain base subunit 1
**610138**	SLC25A13	solute carrier family 25, member 13 (citrin)
**474567**	COX5B	cytochrome c oxidase polypeptide Vb
**474572**	LRPPRC	leucine-rich PPR-motif containing
**481360**	EPCAM	epithelial cell adhesion molecule
**611134**	LOC611134	similar to cytochrome c oxidase, subunit 7a 3
**474598**	RTN4	reticulon 4
**474978**	BCKDHB	branched chain keto acid dehydrogenase E1, beta polypeptide
**484356**	LOC484356	hypothetical LOC484356
**482460**	ZMPSTE24	zinc metallopeptidase (STE24 homolog, S. cerevisiae)
**482464**	PABPC4	poly(A) binding protein, cytoplasmic 4 (inducible form)
**475501**	LOC475501	similar to H2A histone family, member V isoform 5
**482691**	TBRG4	transforming growth factor beta regulator 4
**403774**	RAB1A	RAB1A, member RAS oncogene family
**481418**	PCYOX1	prenylcysteine oxidase 1
**403980**	CAV1	caveolin 1, caveolae protein, 22kDa
**482966**	NOL10	nucleolar protein 10
**404017**	SEC61G	Sec61 gamma subunit
**612336**	LOC612336	similar to Protein FAM3C precursor (Protein GS3786)
**475875**	RALA	v-ral simian leukemia viral oncogene homolog A (ras related)
**476413**	RRAS	related RAS viral (r-ras) oncogene homolog
**474818**	RAB14	RAB14, member RAS oncogene family
**612719**	STOM	stomatin
**477956**	ITGB1	integrin, beta 1 (fibronectin receptor, beta polypeptide, antigen CD29 includes MDF2, MSK12)
**476084**	PGRMC2	progesterone receptor membrane component 2
**475524**	LOC475524	similar to single-stranded DNA binding protein 1 isoform 1
**482764**	AGK	acylglycerol kinase
**475029**	RPF2	ribosome production factor 2 homolog (S. cerevisiae)
**485119**	ENDOD1	endonuclease domain containing 1
**476516**	RPN1	ribophorin I
**404007**	RAB7A	RAB7A, member RAS oncogene family
**484425**	SLC1A5	solute carrier family 1 (neutral amino acid transporter), member 5
**403958**	RAB10	RAB10, member RAS oncogene family
**476101**	UGGT1	UDP-glucose glycoprotein glucosyltransferase 1
**475687**	HADHA	hydroxyacyl-CoA dehydrogenase/3-ketoacyl-CoA thiolase/enoyl-CoA hydratase (trifunctional protein), alpha subunit
**476519**	ACAD9	acyl-CoA dehydrogenase family, member 9
**477002**	ACSS1	acyl-CoA synthetase short-chain family member 1
**477988**	YME1L1	YME1-like 1 (S. cerevisiae)
**484637**	TMEM43	transmembrane protein 43

**485448**	SUCLA2	succinate-CoA ligase, ADP-forming, beta subunit
**611055**	TOMM40	translocase of outer mitochondrial membrane 40 homolog (yeast)
**474433**	LOC474433	similar to ATP synthase, H+ transporting, mitochondrial F0 complex, subunit d isoform a
**476794**	NDUFC2	NADH dehydrogenase (ubiquinone) 1, subcomplex unknown, 2, 14.5kDa
**483318**	ITGB4	integrin, beta 4
**475916**	H3F3B	H3 histone, family 3B (H3.3B)
**484473**	CEACAM28	carcinoembryonic antigen-related cell adhesion molecule 28
**478009**	ATP5C1	ATP synthase, H+ transporting, mitochondrial F1 complex, gamma polypeptide 1
**476130**	UBXN4	UBX domain protein 4
**612817**	SRSF2	serine/arginine-rich splicing factor 2
**477032**	CTNNB1	catenin (cadherin-associated protein), beta 1, 88kDa
**482533**	ELOVL1	ELOVL fatty acid elongase 1
**485187**	SERPINH1	serpin peptidase inhibitor, clade H (heat shock protein 47), member 1, (collagen binding protein 1)
**476461**	TIMM50	translocase of inner mitochondrial membrane 50 homolog (S. cerevisiae)
**475462**	VDAC3	voltage-dependent anion channel 3
**612874**	SUPT16H	suppressor of Ty 16 homolog (S. cerevisiae)
**608330**	RAB6A	RAB6A, member RAS oncogene family
**485637**	SLC4A7	solute carrier family 4, sodium bicarbonate cotransporter, member 7
**612944**	ARL8B	ADP-ribosylation factor-like 8B
**608030**	MRPL12	mitochondrial ribosomal protein L12
**607518**	ERLIN2	ER lipid raft associated 2
**475593**	MAK16	MAK16 homolog (S. cerevisiae)
**477175**	MRPS26	mitochondrial ribosomal protein S26
**477072**	TF	transferrin
**476559**	ARL6IP5	ADP-ribosylation-like factor 6 interacting protein 5
**475436**	SLC25A3	solute carrier family 25 (mitochondrial carrier; phosphate carrier), member 3
**475939**	HSD17B12	hydroxysteroid (17-beta) dehydrogenase 12
**486776**	TIMM23	translocase of inner mitochondrial membrane 23 homolog (yeast)
**612644**	LOC612644	similar to cytochrome c oxidase, subunit VIb polypeptide 1
**403474**	CAT	catalase
**477185**	CSNK2A1	casein kinase 2, alpha 1 polypeptide
**431686**	ATP4A	ATPase, H+/K+ exchanging, alpha polypeptide
**404009**	RAB2A	RAB2A, member RAS oncogene family
**486970**	YTHDF3	YTH domain family, member 3
**486978**	RRS1	RRS1 ribosome biogenesis regulator homolog (S. cerevisiae)
**479137**	REEP5	receptor accessory protein 5
**477347**	PSPC1	paraspeckle component 1
**475764**	IMMT	inner membrane protein, mitochondrial
**475630**	SLC25A4	solute carrier family 25 (mitochondrial carrier; adenine nucleotide translocator), member 4
**477088**	COPB2	coatomer protein complex, subunit beta 2 (beta prime)
**477098**	ATP1B3	ATPase, Na+/K+ transporting, beta 3 polypeptide
**607472**	MYOF	myoferlin
**483489**	CTNND1	catenin (cadherin-associated protein), delta 1
**607299**	NOP10	NOP10 ribonucleoprotein homolog (yeast)
**477362**	FDFT1	farnesyl-diphosphate farnesyltransferase 1
**477103**	U2SURP	U2 snRNP-associated SURP domain containing
**477210**	EIF6	eukaryotic translation initiation factor 6
**476372**	LOC476372	similar to ATP synthase, H+ transporting, mitochondrial F0 complex, subunit f, isoform 2
**475978**	NDUFS3	NADH dehydrogenase (ubiquinone) Fe-S protein 3, 30kDa (NADH-coenzyme Q reductase)
**403878**	ATP2A2	ATPase, Ca++ transporting, cardiac muscle, slow twitch 2

**475979**	CELF1	CUGBP, Elav-like family member 1
**475480**	ETFDH	electron-transferring-flavoprotein dehydrogenase
**477223**	RPN2	ribophorin II
**612786**	TM9SF3	transmembrane 9 superfamily member 3
**608951**	FKBP11	FK506 binding protein 11, 19 kDa
**477124**	SSR3	signal sequence receptor, gamma (translocon-associated protein gamma)
**608725**	RRP12	ribosomal RNA processing 12 homolog (S. cerevisiae)
**479172**	SCAMP1	secretory carrier membrane protein 1
**478059**	LOC478059	similar to F26E4.12
**478755**	GPD2	glycerol-3-phosphate dehydrogenase 2 (mitochondrial)
**488199**	PRPF4B	PRP4 pre-mRNA processing factor 4 homolog B (yeast)
**403951**	SSR1	signal sequence receptor, alpha
**478410**	LOC478410	similar to ATP synthase O subunit, mitochondrial precursor (Oligomycin sensitivity conferral protein) (OSCP)
**403992**	ATP1A1	ATPase, Na+/K+ transporting, alpha 1 polypeptide
**486848**	SFXN3	sideroflexin 3
**478552**	CD47	CD47 molecule
**488289**	LOC488289	similar to Histone H2A.1
**442957**	GNAI2	guanine nucleotide binding protein (G protein), alpha inhibiting activity polypeptide 2
**403583**	CPT1A	carnitine palmitoyltransferase 1A (liver)
**610084**	CISD2	CDGSH iron sulfur domain 2
**486308**	TRPV4	transient receptor potential cation channel, subfamily V, member 4
**488456**	SF3B1	splicing factor 3b, subunit 1, 155kDa
**479192**	HEATR1	HEAT repeat containing 1
**488264**	LOC488264	similar to Histone H1.2 (H1d)
**477660**	MRPS35	mitochondrial ribosomal protein S35
**480830**	SLC25A6	solute carrier family 25 (mitochondrial carrier; adenine nucleotide translocator), member 6
**483166**	LOC483166	similar to germinal histone H4 gene
**476004**	NDUFV1	NADH dehydrogenase (ubiquinone) flavoprotein 1, 51kDa
**486317**	DAO	D-amino-acid oxidase
**611089**	LOC611089	similar to H2B histone family, member F
**483175**	LOC483175	similar to histone H2A
**477264**	DPM1	dolichyl-phosphate mannosyltransferase polypeptide 1, catalytic subunit
**477841**	TIAL1	TIA1 cytotoxic granule-associated RNA binding protein-like 1
**479212**	CISD1	CDGSH iron sulfur domain 1
**489372**	LOC489372	similar to Histone H2A.x (H2a/x)
**608244**	NDUFA10	NADH dehydrogenase (ubiquinone) 1 alpha subcomplex, 10, 42kDa
**478974**	F11R	F11 receptor
**476012**	MRPL11	mitochondrial ribosomal protein L11
**478798**	SLC25A12	solute carrier family 25 (mitochondrial carrier, Aralar), member 12
**403703**	TFRC	transferrin receptor (p90, CD71)
**611961**	BANF1	barrier to autointegration factor 1
**480032**	DHX9	DEAH (Asp-Glu-Ala-His) box polypeptide 9
**478192**	HNRNPR	heterogeneous nuclear ribonucleoprotein R
**478880**	NDUFS1	NADH dehydrogenase (ubiquinone) Fe-S protein 1, 75kDa (NADH-coenzyme Q reductase)
**478663**	TRA2B	transformer 2 beta homolog (Drosophila)
**403728**	ZONAB	Y-box protein ZONAB-A
**480866**	EIF2S3	eukaryotic translation initiation factor 2, subunit 3 gamma, 52kDa
**477695**	LOC477695	similar to mago-nashi homolog
**489816**	POR	P450 (cytochrome) oxidoreductase
**478992**	TMCO1	transmembrane and coiled-coil domains 1
**488421**	AGPS	alkylglycerone phosphate synthase

**491408**	RAB18	RAB18, member RAS oncogene family
**476891**	DDX21	DEAD (Asp-Glu-Ala-Asp) box polypeptide 21
**479728**	FIS1	fission 1 (mitochondrial outer membrane) homolog (S. cerevisiae)
**477700**	M6PR	mannose-6-phosphate receptor (cation dependent)
**483747**	MRPL49	mitochondrial ribosomal protein L49
**489406**	DLAT	dihydrolipoamide S-acetyltransferase
**476659**	NDUFA13	NADH dehydrogenase (ubiquinone) 1 alpha subcomplex, 13
**489042**	MCU	mitochondrial calcium uniporter
**488437**	ITGAV	integrin, alpha V (vitronectin receptor, alpha polypeptide, antigen CD51)
**610791**	TMX1	thioredoxin-related transmembrane protein 1
**488807**	LETM1	leucine zipper-EF-hand containing transmembrane protein 1
**404012**	DDOST	dolichyl-diphosphooligosaccharide--protein glycosyltransferase
**476043**	RTN3	reticulon 3
**476044**	ATL3	atlastin GTPase 3
**487406**	MRTO4	mRNA turnover 4 homolog (S. cerevisiae)
**611024**	LOC611024	similar to 3-beta-hydroxysteroid-delta(8),delta(7)-isomerase (Cholestenol delta-isomerase) (Delta8-delta7 sterol isomerase) (D8-D7 sterol isomerase) (Emopamil-binding protein)
**612432**	LOC612432	similar to CG2943-PA
**403966**	ATP1B1	ATPase, Na+/K+ transporting, beta 1 polypeptide
**489442**	MYBBP1A	MYB binding protein (P160) 1a
**477718**	NDUFA9	NADH dehydrogenase (ubiquinone) 1 alpha subcomplex, 9, 39kDa
**480086**	BRP44	brain protein 44
**479255**	VDAC2	voltage-dependent anion channel 2
**483777**	SLC3A2	solute carrier family 3 (activators of dibasic and neutral amino acid transport), member 2
**607694**	CNP	2',3'-cyclic nucleotide 3' phosphodiesterase
**403773**	RAB8A	RAB8A, member RAS oncogene family
**478217**	SDHB	succinate dehydrogenase complex, subunit B, iron sulfur (Ip)
**479470**	SLC25A11	solute carrier family 25 (mitochondrial carrier; oxoglutarate carrier), member 11
**479261**	LOC479261	similar to R53.5
**403423**	RBM47	RNA binding motif protein 47
**489463**	ACADVL	acyl-CoA dehydrogenase, very long chain
**490385**	PARP1	poly (ADP-ribose) polymerase 1
**480928**	SMC1A	structural maintenance of chromosomes 1A
**489477**	MPDU1	mannose-P-dolichol utilization defect 1
**606860**	LOC606860	similar to Cytochrome c oxidase polypeptide VIc-2
**489102**	FAF2	Fas associated factor family member 2
**489109**	SFXN1	sideroflexin 1
**479778**	LOC479778	similar to fusion (involved in t(12;16) in malignant liposarcoma) (predicted)
**480547**	PHB	prohibitin
**480127**	DAP3	death associated protein 3
**491074**	LOC491074	similar to integrin alpha 3 isoform b, precursor
**480388**	DLST	dihydrolipoamide S-succinyltransferase (E2 component of 2-oxo-glutarate complex)
**610559**	TMED10	transmembrane emp24-like trafficking protein 10 (yeast)
**491080**	LOC491080	similar to CG3408-PA
**478337**	PPIB	peptidylprolyl isomerase B (cyclophilin B)
**480403**	SPTLC2	serine palmitoyltransferase, long chain base subunit 2
**491103**	TRIM25	tripartite motif containing 25
**475148**	MSI2	musashi homolog 2 (Drosophila)
**609693**	SRSF1	serine/arginine-rich splicing factor 1
**490458**	LOC490458	similar to S100 calcium binding protein A16
**480149**	ATP5A1	ATP synthase, H+ transporting, mitochondrial F1 complex, alpha subunit 1, cardiac muscle

**479815**	UQCRC2	ubiquinol-cytochrome c reductase core protein II
**478370**	LOC478370	similar to Cytochrome c oxidase polypeptide Va, mitochondrial precursor
**481018**	ACSL4	acyl-CoA synthetase long-chain family member 4
**610134**	ETFA	electron-transfer-flavoprotein, alpha polypeptide
**489992**	NOMO2	NODAL modulator 2
**490532**	YES1	v-yes-1 Yamaguchi sarcoma viral oncogene homolog 1
**481029**	PGRMC1	progesterone receptor membrane component 1
**489208**	ITGA2	integrin, alpha 2 (CD49B, alpha 2 subunit of VLA-2 receptor)
**480204**	NDUFV2	NADH dehydrogenase (ubiquinone) flavoprotein 2, 24kDa
**480208**	VAPA	VAMP (vesicle-associated membrane protein)-associated protein A, 33kDa
**481048**	AIFM1	apoptosis-inducing factor, mitochondrion-associated, 1
**480626**	SDF2	stromal cell-derived factor 2
**479365**	LOC479365	similar to Brix domain containing protein 2 (Ribosome biogenesis protein Brix)
**489573**	DHCR24	24-dehydrocholesterol reductase
**481073**	HMGB3	high mobility group box 3
**480646**	MYO1C	myosin IC
**479864**	HMOX2	heme oxygenase (decycling) 2
**480651**	PRPF8	PRP8 pre-mRNA processing factor 8 homolog (S. cerevisiae)
**481080**	BCAP31	B-cell receptor-associated protein 31
**479568**	ATAD3B	ATPase family, AAA domain containing 3B
**485062**	NCLN	nicalin
**481082**	SSR4	signal sequence receptor, delta
**491203**	LOC491203	similar to Putative eukaryotic translation initiation factor 3 subunit (eIF-3)
**492244**	L1CAM	L1 cell adhesion molecule
**476756**	ATP5D	ATP synthase, H+ transporting, mitochondrial F1 complex, delta subunit
**612784**	DNAJC11	DnaJ (Hsp40) homolog, subfamily C, member 11
**476758**	LOC476758	similar to basigin isoform 1
**479901**	ATP5F1	ATP synthase, H+ transporting, mitochondrial Fo complex, subunit B1
**479623**	LOC479623	similar to Cytochrome c oxidase subunit IV isoform 1, mitochondrial precursor (COX IV-1) (Cytochrome c oxidase polypeptide IV)
**611922**	SLC25A24	solute carrier family 25 (mitochondrial carrier; phosphate carrier), member 24
**479633**	GCSH	glycine cleavage system protein H (aminomethyl carrier)
**490153**	F3	coagulation factor III (thromboplastin, tissue factor)
**479939**	ABCD3	ATP-binding cassette, sub-family D (ALD), member 3
**612406**	ZNF326	zinc finger protein 326
**610942**	LOC610942	similar to cytochrome b5 outer mitochondrial membrane precursor
**483035**	CEBPZ	CCAAT/enhancer binding protein (C/EBP), zeta
**608348**	SLC25A1	solute carrier family 25 (mitochondrial carrier; citrate transporter), member 1
**488273**	LOC488273	similar to Histone H1.5 (Histone H1a)
**488258**	LOC488258	similar to Histone H1.2 (H1d)
**483167**	LOC483167	similar to H3 histone, family 2 isoform 2
**483168**	LOC483168	similar to Histone H2A.o (H2A/o) (H2A.2) (H2a-615)
**475214**	NDUFA5	NADH dehydrogenase (ubiquinone) 1 alpha subcomplex, 5, 13kDa
**608656**	LOC608656	similar to oxoglutarate (alpha-ketoglutarate) dehydrogenase (lipoamide) isoform 1 precursor
**610569**	RPS27L	ribosomal protein S27-like

Cluster 5

Enriched Terms:

Gene to GO BP Conditional test for over-representation GOBPID	Pvalue	OddsRatio	ExpCount	Count	Size	Term
**GO:0043436**	0	2,601	13	28	181	oxoacid metabolic process
**GO:0055114**	0	2,555	13	26	169	oxidation-reduction process
**GO:0006105**	0	25,632	0	4	6	succinate metabolic process
**GO:0002520**	0,001	3,102	6	15	80	immune system development
**GO:0048878**	0,001	3,005	6	15	82	chemical homeostasis
**GO:0040027**	0,001	17,078	1	4	7	negative regulation of vulval development
**GO:0061062**	0,001	17,078	1	4	7	regulation of nematode larval development
**GO:0043648**	0,001	6,658	1	6	18	dicarboxylic acid metabolic process
**GO:0065008**	0,001	1,917	24	38	322	regulation of biological quality
**GO:0032496**	0,002	5,05	2	7	25	response to lipopolysaccharide
**GO:0032787**	0,002	2,783	6	14	81	monocarboxylic acid metabolic process
**GO:0006084**	0,002	4,781	2	7	26	acetyl-CoA metabolic process
**GO:0051186**	0,005	2,679	5	12	71	cofactor metabolic process
**GO:0045454**	0,005	4,551	2	6	23	cell redox homeostasis
**GO:0050804**	0,005	4,551	2	6	23	regulation of synaptic transmission
**GO:0001837**	0,005	Inf	0	2	2	epithelial to mesenchymal transition
**GO:0048147**	0,005	Inf	0	2	2	negative regulation of fibroblast proliferation
**GO:0051588**	0,005	Inf	0	2	2	regulation of neurotransmitter transport
**GO:2000300**	0,005	Inf	0	2	2	regulation of synaptic vesicle exocytosis
**GO:0007399**	0,006	1,867	17	27	225	nervous system development
**GO:0006091**	0,006	2,486	6	13	82	generation of precursor metabolites and energy
**GO:0006457**	0,006	2,722	5	11	64	protein folding
**GO:0030097**	0,006	2,588	5	12	73	hemopoiesis
**GO:0006099**	0,006	5,349	1	5	17	tricarboxylic acid cycle
**GO:0030866**	0,006	7,303	1	4	11	cortical actin cytoskeleton organization
**GO:0048581**	0,006	7,303	1	4	11	negative regulation of post-embryonic development
**GO:0022904**	0,007	4,296	2	6	24	respiratory electron transport chain
**GO:0002562**	0,007	12,715	0	3	6	somatic diversification of immune receptors via germline recombination within a single locus
**GO:0006664**	0,007	12,715	0	3	6	glycolipid metabolic process
**GO:0055072**	0,007	12,715	0	3	6	iron ion homeostasis
**GO:0008406**	0,008	4,068	2	6	25	gonad development
**GO:0045333**	0,009	4,743	1	5	19	cellular respiration
**GO:0006873**	0,01	2,525	5	11	68	cellular ion homeostasis

Genes in cluster:

Entrez Id	Symbol	description
**474403**	CS	citrate synthase

**607115**	CNPY2	canopy 2 homolog (zebrafish)
**610360**	FECH	ferrochelatase
**476198**	ME2	malic enzyme 2, NAD(+)-dependent, mitochondrial
**481134**	TSFM	Ts translation elongation factor, mitochondrial
**442965**	UTRN	utrophin
**476245**	MTHFD1L	methylenetetrahydrofolate dehydrogenase (NADP+ dependent) 1-like
**481489**	PRRC1	proline-rich coiled-coil 1
**476258**	SOD2	superoxide dismutase 2, mitochondrial
**607061**	TRABD	TraB domain containing
**474473**	SAMM50	sorting and assembly machinery component 50 homolog (S. cerevisiae)
**481226**	NAGA	N-acetylgalactosaminidase, alpha-
**481234**	CHADL	chondroadherin-like
**481524**	HNRNPA0	heterogeneous nuclear ribonucleoprotein A0
**474700**	MATR3	matrin 3
**475201**	CALU	calumenin
**608746**	TOM1	target of myb1 (chicken)
**481576**	ACO1	aconitase 1, soluble
**481582**	NOL6	nucleolar protein family 6 (RNA-associated)
**484188**	ERMP1	endoplasmic reticulum metallopeptidase 1
**403689**	TRIM28	tripartite motif containing 28
**482343**	MACC1	metastasis associated in colon cancer 1
**404018**	SEC61B	Sec61 beta subunit
**481884**	MYO6	myosin VI
**482355**	GPNMB	glycoprotein (transmembrane) nmb
**481376**	PNPT1	polyribonucleotide nucleotidyltransferase 1
**479610**	HIBADH	3-hydroxyisobutyrate dehydrogenase
**403550**	ALB	albumin
**610135**	LOC610135	similar to small nuclear ribonucleoprotein polypeptide G
**503863**	CAPZA2	capping protein (actin filament) muscle Z-line, alpha 2
**475668**	PDIA6	protein disulfide isomerase family A, member 6
**475518**	GSTK1	glutathione S-transferase kappa 1
**484388**	SNRNP70	small nuclear ribonucleoprotein 70kDa (U1)
**403978**	DLD	dihydrolipoamide dehydrogenase
**403523**	BAX	BCL2-associated X protein
**484391**	NUCB1	nucleobindin 1
**484417**	EHD2	EH-domain containing 2
**612960**	NAPA	N-ethylmaleimide-sensitive factor attachment protein, alpha
**476773**	CHORDC1	cysteine and histidine-rich domain (CHORD) containing 1
**475374**	LOC475374	similar to Alcohol dehydrogenase [NADP+] (Aldehyde reductase) (Aldo-keto reductase family 1 member A1)
**477023**	ACAA1	acetyl-CoA acyltransferase 1
**485604**	ENTPD3	ectonucleoside triphosphate diphosphohydrolase 3
**475713**	WDR43	WD repeat domain 43
**476152**	PRPF40A	PRP40 pre-mRNA processing factor 40 homolog A (S. cerevisiae)
**483369**	P4HB	prolyl 4-hydroxylase, beta polypeptide
**477294**	MRPS31	mitochondrial ribosomal protein S31
**475730**	SRSF7	serine/arginine-rich splicing factor 7
**482609**	SNRPF	small nuclear ribonucleoprotein polypeptide F
**476831**	PRKCDBP	protein kinase C, delta binding protein
**482618**	TMPO	thymopoietin
**403170**	HMGB1	high mobility group box 1
**476562**	SUCLG2	succinate-CoA ligase, GDP-forming, beta subunit
**475742**	SNRNP200	small nuclear ribonucleoprotein 200kDa (U5)
**477597**	PFDN5	prefoldin subunit 5
**476574**	PDHB	pyruvate dehydrogenase (lipoamide) beta

**485385**	SWAP70	SWAP switching B-cell complex 70kDa subunit
**404019**	HSP90B1	heat shock protein 90kDa beta (Grp94), member 1
**488888**	LOC488888	hypothetical LOC488888
**476503**	LOC476503	similar to ubiquinol-cytochrome c reductase, Rieske iron-sulfur polypeptide 1
**486068**	HMGB2	high mobility group box 2
**475775**	SUCLG1	succinate-CoA ligase, alpha subunit
**477781**	ALDH18A1	aldehyde dehydrogenase 18 family, member A1
**477482**	ERP29	endoplasmic reticulum protein 29
**477488**	PTPN11	protein tyrosine phosphatase, non-receptor type 11
**487050**	CDH17	cadherin 17, LI cadherin (liver-intestine)
**607040**	HIBCH	3-hydroxyisobutyryl-CoA hydrolase
**478934**	SNRPE	small nuclear ribonucleoprotein polypeptide E
**479700**	GBAS	glioblastoma amplified sequence
**608421**	MANF	mesencephalic astrocyte-derived neurotrophic factor
**488246**	ALDH5A1	aldehyde dehydrogenase 5 family, member A1
**610883**	ACOT13	acyl-CoA thioesterase 13
**489293**	SRPR	signal recognition particle receptor (docking protein)
**490236**	RNPEP	arginyl aminopeptidase (aminopeptidase B)
**403739**	COL17A1	collagen, type XVII, alpha 1
**403486**	PPP3CA	protein phosphatase 3, catalytic subunit, alpha isozyme
**607017**	SBDS	Shwachman-Bodian-Diamond syndrome
**478854**	HSPD1	heat shock 60kDa protein 1 (chaperonin)
**478855**	LOC478855	similar to heat shock 10kDa protein 1 (chaperonin 10)
**487993**	POGLUT1	protein O-glucosyltransferase 1
**478634**	SDHA	succinate dehydrogenase complex, subunit A, flavoprotein (Fp)
**478963**	IARS2	isoleucyl-tRNA synthetase 2, mitochondrial
**478506**	HADH	hydroxyacyl-CoA dehydrogenase
**479714**	GTF2I	general transcription factor IIi
**480255**	PCK2	phosphoenolpyruvate carboxykinase 2 (mitochondrial)
**479043**	IDH2	isocitrate dehydrogenase 2 (NADP+), mitochondrial
**477839**	LOC477839	similar to Thioredoxin-dependent peroxide reductase, mitochondrial precursor (Peroxiredoxin 3) (Antioxidant protein 1) (AOP-1) (MER5 protein homolog) (HBC189) (PRX III)
**479410**	HYOU1	hypoxia up-regulated 1
**480971**	LOC480971	similar to Ewing sarcoma breakpoint region 1 isoform EWS
**477686**	EPS8	epidermal growth factor receptor pathway substrate 8
**488648**	PFDN2	prefoldin subunit 2
**488664**	UAP1	UDP-N-acteylglucosamine pyrophosphorylase 1
**478279**	PDIA3	protein disulfide isomerase family A, member 3
**491776**	PRDX4	peroxiredoxin 4
**480864**	ACOT9	acyl-CoA thioesterase 9
**607482**	BCR	breakpoint cluster region
**477554**	LOC477554	similar to small nuclear ribonucleoprotein D3
**403907**	PNN	pinin, desmosome associated protein
**491830**	MID1IP1	MID1 interacting protein 1 (gastrulation specific G12 homolog (zebrafish))
**490935**	EFTUD2	elongation factor Tu GTP binding domain containing 2
**479446**	RDX	radixin
**489421**	ACAT1	acetyl-CoA acetyltransferase 1
**403934**	CDC42	cell division cycle 42 (GTP binding protein, 25kDa)
**490691**	ERO1L	ERO1-like (S. cerevisiae)
**607705**	ATP6V0A1	ATPase, H+ transporting, lysosomal V0 subunit a1
**477715**	NCAPD2	non-SMC condensin I complex, subunit D2
**478210**	AKR7A2	aldo-keto reductase family 7, member A2 (aflatoxin aldehyde reductase)
**490967**	STAT3	signal transducer and activator of transcription 3 (acute-phase response factor)
**483780**	NXF1	nuclear RNA export factor 1
**403941**	RAB5C	RAB5C, member RAS oncogene family

**479752**	AIMP2	aminoacyl tRNA synthetase complex-interacting multifunctional protein 2
**480092**	FH	fumarate hydratase
**483784**	GANAB	glucosidase, alpha; neutral AB
**490975**	FKBP10	FK506 binding protein 10, 65 kDa
**403674**	EIF1	eukaryotic translation initiation factor 1
**487421**	EPHA2	EPH receptor A2
**480112**	PYCR2	pyrroline-5-carboxylate reductase family, member 2
**479116**	PGM2	phosphoglucomutase 2
**480930**	HSD17B10	hydroxysteroid (17-beta) dehydrogenase 10
**480130**	MTX1	metaxin 1
**490789**	EIF2B2	eukaryotic translation initiation factor 2B, subunit 2 beta, 39kDa
**476694**	CALR	calreticulin
**476699**	ASNA1	arsA arsenite transporter, ATP-binding, homolog 1 (bacterial)
**479796**	TUFM	Tu translation elongation factor, mitochondrial
**490810**	LOC490810	similar to CG33071-PC, isoform C
**479313**	CLINT1	clathrin interactor 1
**484941**	PRKCSH	protein kinase C substrate 80K-H
**476708**	RAB3D	RAB3D, member RAS oncogene family
**479805**	NDUFAB1	NADH dehydrogenase (ubiquinone) 1, alpha/beta subcomplex, 1, 8kDa
**611211**	AP1M2	adaptor-related protein complex 1, mu 2 subunit
**490500**	DSG2	desmoglein 2
**480605**	AP2B1	adaptor-related protein complex 2, beta 1 subunit
**480178**	SNRPD1	small nuclear ribonucleoprotein D1 polypeptide 16kDa
**479331**	CSNK1A1	casein kinase 1, alpha 1
**491142**	NLE1	notchless homolog 1 (Drosophila)
**403880**	STXBP2	syntaxin binding protein 2
**485005**	TIMM44	translocase of inner mitochondrial membrane 44 homolog (yeast)
**481037**	LAMP2	lysosomal-associated membrane protein 2
**481038**	MCTS1	malignant T cell amplified sequence 1
**611425**	LONP1	lon peptidase 1, mitochondrial
**480635**	BLMH	bleomycin hydrolase
**489585**	CPT2	carnitine palmitoyltransferase 2
**479564**	SCP2	sterol carrier protein 2
**479880**	RNPS1	RNA binding protein S1, serine-rich domain
**611859**	POLR2E	polymerase (RNA) II (DNA directed) polypeptide E, 25kDa
**480677**	PMPCA	peptidase (mitochondrial processing) alpha
**611810**	GNAI3	guanine nucleotide binding protein (G protein), alpha inhibiting activity polypeptide 3
**479659**	SF3B3	splicing factor 3b, subunit 3, 130kDa
**480726**	HSPA5	heat shock 70kDa protein 5 (glucose-regulated protein, 78kDa)
**479685**	ATP6V0D1	ATPase, H+ transporting, lysosomal 38kDa, V0 subunit d1
**490679**	ARF6	ADP-ribosylation factor 6
**488269**	LOC488269	similar to Histone H1.4 (Histone H1b)
**476018**	DRAP1	DR1-associated protein 1 (negative cofactor 2 alpha)

Cluster 6

Enriched Terms:

Gene to GO BP Conditional test for over-representation GOBPID	Pvalue	OddsRatio	ExpCount	Count	Size	Term
**GO:0006414**	0	23,644	7	48	79	translational elongation
**GO:0009059**	0	7,008	16	57	211	macromolecule biosynthetic process
**GO:0000022**	0	14,465	3	20	37	mitotic spindle elongation
**GO:0044260**	0	3,342	71	112	857	cellular macromolecule metabolic process
**GO:0044249**	0	3,437	28	59	361	cellular biosynthetic process
**GO:0019538**	0	2,864	50	86	589	protein metabolic process
**GO:0071843**	0	5,065	8	27	94	cellular component biogenesis at cellular level
**GO:0006364**	0	7,713	3	15	38	rRNA processing
**GO:0007051**	0	4,144	8	24	95	spindle organization
**GO:0006396**	0	2,627	18	36	209	RNA processing
**GO:0007017**	0	2,835	13	28	149	microtubule-based process
**GO:0043487**	0	13,202	1	7	13	regulation of RNA stability
**GO:0042254**	0	8,336	2	8	20	ribosome biogenesis
**GO:0008152**	0	2,178	112	132	1326	metabolic process
**GO:0048255**	0	13,498	1	6	11	mRNA stabilization
**GO:0070925**	0	7,567	2	8	20	organelle assembly
**GO:0042273**	0	11,242	1	6	12	ribosomal large subunit biogenesis
**GO:0034660**	0	3,161	6	16	75	ncRNA metabolic process
**GO:0000028**	0,001	22,245	1	4	6	ribosomal small subunit assembly
**GO:0006417**	0,001	4,07	3	10	38	regulation of translation
**GO:0090304**	0,001	1,751	39	56	465	nucleic acid metabolic process
**GO:0010556**	0,001	2,103	15	27	180	regulation of macromolecule biosynthetic process
**GO:0022403**	0,002	2,13	14	25	164	cell cycle phase
**GO:0031326**	0,002	1,965	17	29	205	regulation of cellular biosynthetic process
**GO:0007010**	0,004	1,88	19	30	220	cytoskeleton organization
**GO:0010468**	0,004	1,821	21	33	250	regulation of gene expression
**GO:0071826**	0,004	3,392	3	9	39	ribonucleoprotein complex subunit organization
**GO:0000291**	0,007	Inf	0	2	2	nuclear-transcribed mRNA catabolic process, exonucleolytic
**GO:0006412**	0,007	2,63	5	11	83	translation
**GO:0006402**	0,008	5,062	1	5	16	mRNA catabolic process
**GO:0042157**	0,01	11,045	1	3	6	lipoprotein metabolic process

Genes in cluster:

Entrez Id	Symbol	description
**478509**	LOC478509	similar to ribosomal protein L34
**490572**	LOC490572	similar to myosin, light polypeptide 6, alkali, smooth muscle and non-muscle
**474830**	TUBB	tubulin, beta
**612751**	PABPC1	poly(A) binding protein, cytoplasmic 1
**476328**	LOC476328	similar to 60S ribosomal protein L29 (Cell surface heparin binding protein HIP)
**474672**	P4HA2	prolyl 4-hydroxylase, alpha polypeptide II

**403685**	RPS18	ribosomal protein S18
**609684**	LOC609684	similar to breast cancer membrane protein 101
**474877**	LOC474877	similar to 40S ribosomal protein S10
**481774**	LOC481774	similar to U1 small nuclear ribonucleoprotein C (U1 snRNP protein C) (U1C protein) (U1-C)
**607574**	CHCHD3	coiled-coil-helix-coiled-coil-helix domain containing 3
**474698**	LOC474698	similar to catenin (cadherin-associated protein), alpha 1, 102kDa
**474656**	LOC474656	similar to ribosomal protein L10a
**474504**	LOC474504	similar to 60S ribosomal protein L3 (L4)
**474887**	SRPK1	SRSF protein kinase 1
**482266**	FLNC	filamin C, gamma
**475203**	RBM28	RNA binding motif protein 28
**475130**	RPL8	ribosomal protein L8
**474524**	LOC474524	similar to CG9987-PA
**403855**	PON2	paraoxonase 2
**481543**	LOC481543	similar to ribosomal protein L31
**476366**	RPS5	ribosomal protein S5
**482437**	SLC2A1	solute carrier family 2 (facilitated glucose transporter), member 1
**476387**	RPS9	ribosomal protein S9
**479350**	LOC479350	similar to ribosomal protein L37
**403684**	RPS11	ribosomal protein S11
**442938**	FLT3LG	fms-related tyrosine kinase 3 ligand
**476422**	RPL18	ribosomal protein L18
**475889**	SYPL1	synaptophysin-like 1
**484627**	LOC484627	similar to H1 histone family, member X
**608089**	LOC608089	similar to Adipocyte plasma membrane-associated protein (BSCv protein)
**476780**	PICALM	phosphatidylinositol binding clathrin assembly protein
**476523**	MRPS25	mitochondrial ribosomal protein S25
**475381**	RPS8	ribosomal protein S8
**475410**	LOC475410	similar to 60S ribosomal protein L32
**476120**	DDX18	DEAD (Asp-Glu-Ala-Asp) box polypeptide 18
**476450**	RPS19	ribosomal protein S19
**477031**	LOC477031	similar to ribosomal protein L14
**478018**	PITRM1	pitrilysin metallopeptidase 1
**476804**	RPS3	ribosomal protein S3
**485628**	STT3B	STT3, subunit of the oligosaccharyltransferase complex, homolog B (S. cerevisiae)
**476463**	RPS16	ribosomal protein S16
**478021**	GTPBP4	GTP binding protein 4
**475922**	EIF4A3	eukaryotic translation initiation factor 4A3
**476466**	HNRNPL	heterogeneous nuclear ribonucleoprotein L
**477046**	RPL15	ribosomal protein L15
**483038**	CYP1B1	cytochrome P450, family 1, subfamily B, polypeptide 1
**475044**	LOC475044	similar to Heterogeneous nuclear ribonucleoprotein A1 (Helix-destabilizing protein) (Single-strand binding protein) (hnRNP core protein A1) (HDP)
**612871**	NOP56	NOP56 ribonucleoprotein homolog (yeast)
**475942**	PDHX	pyruvate dehydrogenase complex, component X
**476842**	LOC476842	similar to ribosomal protein L27a
**607420**	CAPRIN1	cell cycle associated protein 1
**477760**	BMS1	BMS1 homolog, ribosome assembly protein (yeast)
**478067**	NDUFAF2	NADH dehydrogenase (ubiquinone) 1 alpha subcomplex, assembly factor 2
**486246**	DDX55	DEAD (Asp-Glu-Ala-Asp) box polypeptide 55
**485845**	RALY	RNA binding protein, autoantigenic (hnRNP-associated with lethal yellow homolog (mouse))
**477197**	EIF2S2	eukaryotic translation initiation factor 2, subunit 2 beta, 38kDa
**403677**	RPS3A	ribosomal protein S3A
**477912**	RPL7	ribosomal protein L7
**479257**	LOC479257	similar to ribosomal protein S24 isoform 3

**442971**	CLU	clusterin
**477112**	EIF2A	eukaryotic translation initiation factor 2A, 65kDa
**612261**	MRPL53	mitochondrial ribosomal protein L53
**608138**	TUBA1C	tubulin, alpha 1c
**479159**	RPS23	ribosomal protein S23
**480235**	LOC480235	similar to ribosomal protein S13
**487034**	CPNE3	copine III
**403691**	RPL6	Ribosomal protein L6
**476976**	HNRPDL	heterogeneous nuclear ribonucleoprotein D-like
**486112**	STC1	stanniocalcin 1
**488443**	WDR75	WD repeat domain 75
**478701**	TUBB2A	tubulin, beta 2A
**477944**	LOC477944	similar to ubiquinol-cytochrome c reductase binding protein
**475805**	BCAS2	breast carcinoma amplified sequence 2
**475991**	RPLP2	ribosomal protein, large, P2
**478547**	RPL24	ribosomal protein L24
**475807**	CSDE1	cold shock domain containing E1, RNA-binding
**477801**	NPM3	nucleophosmin/nucleoplasmin 3
**479011**	YBX1	Y box binding protein 1
**477505**	GCN1L1	GCN1 general control of amino-acid synthesis 1-like 1 (yeast)
**477510**	SRSF9	serine/arginine-rich splicing factor 9
**486886**	SMC3	structural maintenance of chromosomes 3
**489300**	STT3A	STT3, subunit of the oligosaccharyltransferase complex, homolog A (S. cerevisiae)
**608445**	NCL	nucleolin
**476824**	LOC476824	similar to NADH-ubiquinone oxidoreductase 23 kDa subunit, mitochondrial precursor (Complex I-23KD) (CI-23KD) (TYKY subunit)
**478158**	PUM1	pumilio homolog 1 (Drosophila)
**477258**	STAU1	staufen, RNA binding protein, homolog 1 (Drosophila)
**478161**	YTHDF2	YTH domain family, member 2
**608455**	UQCRC1	ubiquinol-cytochrome c reductase core protein I
**610648**	RBM4	RNA binding motif protein 4
**479412**	RPS25	ribosomal protein S25
**607667**	NOP58	NOP58 ribonucleoprotein homolog (yeast)
**478876**	WDR12	WD repeat domain 12
**478973**	COPA	coatomer protein complex, subunit alpha
**478597**	RPL35A	ribosomal protein L35a
**478981**	NDUFS2	NADH dehydrogenase (ubiquinone) Fe-S protein 2, 49kDa (NADH-coenzyme Q reductase)
**610762**	RAB1B	RAB1B, member RAS oncogene family
**403681**	RPS17	ribosomal protein S17
**478662**	IGF2BP2	insulin-like growth factor 2 mRNA binding protein 2
**608074**	HNRNPA3	heterogeneous nuclear ribonucleoprotein A3
**478664**	DNAJB11	DnaJ (Hsp40) homolog, subfamily B, member 11
**403788**	ITGB3	integrin, beta 3 (platelet glycoprotein IIIa, antigen CD61)
**442942**	PTGS2	prostaglandin-endoperoxide synthase 2 (prostaglandin G/H synthase and cyclooxygenase)
**480751**	RPL30	ribosomal protein L30
**480494**	NMT1	N-myristoyltransferase 1
**479886**	RPS2	ribosomal protein S2
**479234**	HK1	hexokinase 1
**483748**	FAU	Finkel-Biskis-Reilly murine sarcoma virus (FBR-MuSV) ubiquitously expressed
**610596**	RPS29	ribosomal protein S29
**480503**	UBTF	upstream binding transcription factor, RNA polymerase I
**478828**	NCKAP1	NCK-associated protein 1
**478904**	RPL37A	ribosomal protein L37a
**476664**	UPF1	UPF1 regulator of nonsense transcripts homolog (yeast)
**403688**	RPL27	ribosomal protein L27

**489047**	DNAJC9	DnaJ (Hsp40) homolog, subfamily C, member 9
**611645**	NOP2	NOP2 nucleolar protein homolog (yeast)
**476637**	LOC476637	similar to ribosomal protein L18a
**478213**	TAS1R2	taste receptor, type 1, member 2
**479109**	RPL9	ribosomal protein L9
**480361**	EIF2S1	eukaryotic translation initiation factor 2, subunit 1 alpha, 35kDa
**478325**	MYO1E	myosin IE
**491064**	IGF2BP1	insulin-like growth factor 2 mRNA binding protein 1
**489409**	LOC489409	similar to secretory carrier membrane protein 3 isoform 1
**476525**	LOC476525	similar to 60S ribosomal protein L26 (Silica-induced gene 20 protein) (SIG-20)
**607081**	LOC607081	similar to non-POU domain containing, octamer-binding
**403893**	RPS4Y1	ribosomal protein S4, Y-linked 1
**478348**	LOC478348	similar to ribosomal protein L4
**480139**	ILF2	interleukin enhancer binding factor 2, 45kDa
**478356**	RPLP1	ribosomal protein, large, P1
**612929**	LARP1	La ribonucleoprotein domain family, member 1
**403678**	RPL7A	Ribosomal protein L7a
**484950**	ILF3	interleukin enhancer binding factor 3, 90kDa
**610121**	INTS2	integrator complex subunit 2
**610402**	HNF1B	HNF1 homeobox B
**490868**	CDC42BPB	CDC42 binding protein kinase beta (DMPK-like)
**480466**	LOC480466	similar to ELAV-like protein 1 (Hu-antigen R) (HuR)
**491153**	MYO1D	myosin ID
**492093**	SLC25A5	solute carrier family 25 (mitochondrial carrier; adenine nucleotide translocator), member 5
**485010**	RPS28	ribosomal protein S28
**611619**	RAB11B	RAB11B, member RAS oncogene family
**479347**	OXCT1	3-oxoacid CoA transferase 1
**479360**	NUP155	nucleoporin 155kDa
**490560**	AFG3L2	AFG3 ATPase family gene 3-like 2 (S. cerevisiae)
**479847**	RSL1D1	ribosomal L1 domain containing 1
**479283**	LOC479283	similar to Activated RNA polymerase II transcriptional coactivator p15 (Positive cofactor 4) (PC4) (p14)
**475077**	RPL17	ribosomal protein L17
**403912**	GNB1	guanine nucleotide binding protein (G protein), beta polypeptide 1
**479569**	MRPL20	mitochondrial ribosomal protein L20
**403914**	GNA11	guanine nucleotide binding protein (G protein), alpha 11 (Gq class)
**485070**	LMNB2	lamin B2
**490064**	TBL3	transducin (beta)-like 3
**479887**	NDUFB10	NADH dehydrogenase (ubiquinone) 1 beta subcomplex, 10, 22kDa
**476755**	CIRBP	cold inducible RNA binding protein
**481085**	RPL10	ribosomal protein L10
**479587**	RPL22	ribosomal protein L22
**485097**	PTBP1	polypyrimidine tract binding protein 1
**608025**	TUBB3	tubulin, beta 3
**479612**	RPL13	ribosomal protein L13
**479929**	DBT	dihydrolipoamide branched chain transacylase E2
**476346**	RPL12	ribosomal protein L12
**479947**	TMED5	transmembrane emp24 protein transport domain containing 5
**480729**	RPL35	ribosomal protein L35
**442858**	CDH1	cadherin 1, type 1, E-cadherin (epithelial)
**489745**	ESRP2	epithelial splicing regulatory protein 2
**475827**	RBM8A	RNA binding motif protein 8A
**476753**	RPS15	ribosomal protein S15
**474599**	RPS27A	ribosomal protein S27a
**476738**	RPL36	ribosomal protein L36

**478450**	HNRNPD	heterogeneous nuclear ribonucleoprotein D (AU-rich element RNA binding protein 1, 37kDa)
**478576**	LOC478576	similar to ribosomal protein S15a
