# Supplementary material for: Alteration of Protein Levels during Influenza Virus H1N1 Infection in Host Cells: A Proteomic Survey of Host and Virus Reveals Differential Dynamics
Source: PLoS One. 2014 Apr 9;9(4):e94257. doi: 10.1371/journal.pone.0094257 (PMC3981805; doi:10.1371/journal.pone.0094257)
Supplement: Methods S1 — Supporting methods. (DOC) [file pone.0094257.s008.doc]

**Supporting Information**

# Supporting Methods

**Time course of nucleoprotein expression in MDCK cells after influenza A/PR8/34 infection – Immunofluorescence microscopy.**

MDCK cells were seeded on glas slides in 6-well plates and incubated over night in growth medium. Influenza A/PR8/34 was diluted in infection medium (DMEM, 0.2 % BSA) and the cells were infected at MOI 100 for 1 hour at 37 °C before the medium was replaced with fresh infection medium. At the designated time points, the cells were fixed in 2 % paraformaldehyde and stained using monoclonal anti-NP antibodies (millipore) followed by secondary antibody staining (anti-mouse, Alexa568, life technologies). The cellular DNA was counterstained with DAPI and the cells were imaged using an Olympus FV-1000 confocal microscope.
